# Design Guidelines for Cationic Pillar[n]arenes that Prevent Biofilm Formation by Gram-Positive Pathogens

**DOI:** 10.1021/acsinfecdis.0c00662

**Published:** 2021-03-04

**Authors:** Dana Kaizerman-Kane, Maya Hadar, Roymon Joseph, Dana Logviniuk, Yossi Zafrani, Micha Fridman, Yoram Cohen

**Affiliations:** † School of Chemistry, Sackler Faculty of Exact Sciences, Tel Aviv University, Ramat Aviv, Tel Aviv 69978, Israel; § Department of Organic Chemistry, Israel Institute for Biological Research, Ness-Ziona 74000, Israel

**Keywords:** bacterial biofilm, antibiofilm agents, cationic pillararenes, SAR, Gram-positive

## Abstract

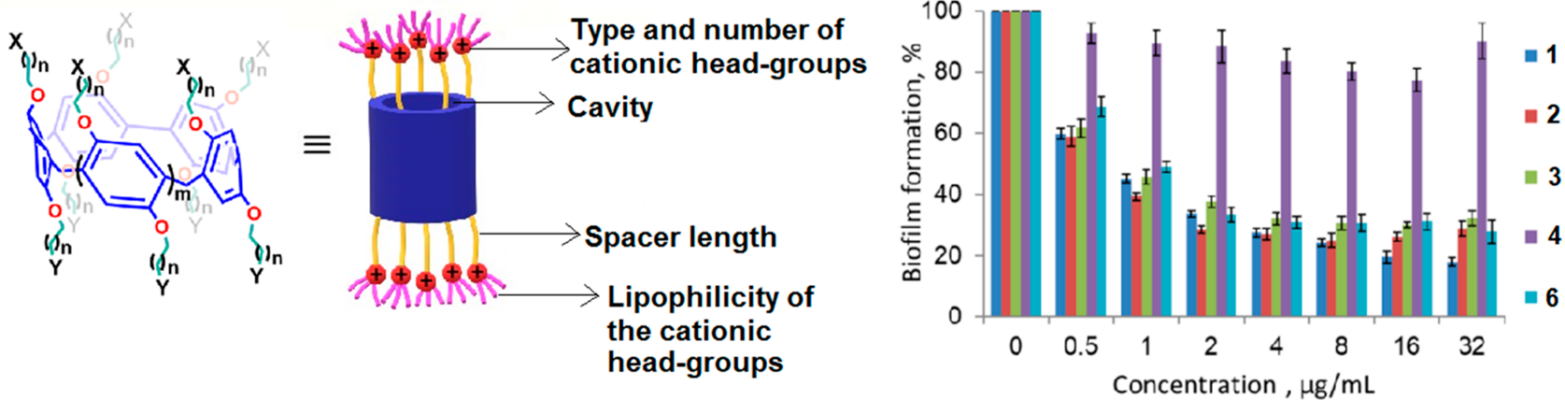

Bacterial biofilms are a major threat to human health, causing persistent infections that lead to millions of fatalities worldwide every year. Biofilms also cause billions of dollars of damage annually by interfering with industrial processes. Recently, cationic pillararenes were found to be potent inhibitors of biofilm formation in Gram-positive bacteria. To identify the structural features of pillararenes that result in antibiofilm activity, we evaluated the activity of 16 cationic pillar[5]arene derivatives including that of the first cationic water-soluble pillar[5]arene-based rotaxane. Twelve of the derivatives were potent inhibitors of biofilm formation by Gram-positive pathogens. Structure activity analyses of our pillararene derivatives indicated that positively charged head groups are critical for the observed antibiofilm activity. Although certain changes in the lipophilicity of the substituents on the positively charged head groups are tolerated, dramatic elevation in the hydrophobicity of the substituents or an increase in steric bulk on these positive charges abolishes the antibiofilm activity. An increase in the overall positive charge from 10 to 20 did not affect the activity significantly, but pillararenes with 5 positive charges and 5 long alkyl chains had reduced activity. Surprisingly, the cavity of the pillar[n]arene is not essential for the observed activity, although the macrocyclic structure of the pillar[n]arene core, which facilitates the clustering of the positive charges, appears important. Interestingly, the compounds found to be efficient inhibitors of biofilm formation were nonhemolytic at concentrations that are ∼100-fold of their MBIC_50_ (the minimal concentration of a compound at which at least 50% inhibition of biofilm formation was observed compared to untreated cells). The structure–activity relationship guidelines established here pave the way for a rational design of potent cationic pillar[n]arene-based antibiofilm agents.

Persistent and chronic infections are serious universal threats to humans, taking millions of lives each year. The National Institutes of Health (NIH) revealed that approximately 80% of recurrent microbial infections in the human body are associated with bacterial biofilms.^1−4^ Bacterial biofilms are microbial colonies, which are embedded in a self-produced matrix of extracellular polymeric substances (EPSs) that protect the microorganism from harsh environmental conditions, antibiotics, and the host immune system.^5−8^ Bacterial biofilms are very common modes of life that can form in most environments on earth and, therefore, may affect many aspects of modern healthcare and industrial processes.^1−8^ In the human body, for example, dental plaque is one of the most well-known and prevalent examples of a bacterial biofilm. These biofilms form on tooth surfaces, and bacterial metabolism in plaque causes tooth decay and gum disease.^9^ Microbial cells within biofilms are more resistant to antibiotics than planktonic cells.^10−12^ Biofilm-associated infections can occur on damaged tissues such as wounds and burns and during lung, cardiac valve, or urinary tract infections. In addition, bacterial biofilm can develop on biomedical implants and devices such as sutures, heart valves, catheters, contact lenses, and dental implants.^13−16^

In recent years, there has been a constant search for new antibiofilm agents, which will effectively inhibit biofilm formation for a long period. This can be achieved by developing antivirulent agents that inhibit biofilm formation, thus reducing the risk for the development of bacterial resistance to these agents. As reported previously, cationic amphiphiles,^17−21^ especially quaternary ammonium cations (QACs), are known to be one of the most potent families of antibacterial and antibiofilm agents.^19,20^ In recent years, there have been many attempts to develop cationic amphiphilic agents with improved antibiofilm activity^17−22^ and reduced toxicity to mammalian cells.^22−26^ For example, Böttcher et al. reported the synthesis of cationic amphiphilic compounds bearing guanidinium and bis-guanidinium groups, which prevent biofilm formation of *Bacillus subtilis* and *Staphylococcus aureus* strains.^22^ Jennings et al. synthesized a library of QACs that serve as simple antimicrobial peptides mimics.^23,24^ These compounds had antimicrobial activity against several Gram-positive and Gram-negative bacterial strains. Some of these compounds efficiently eradicate existing biofilms of *S. aureus* and *Enterococcus faecalis*, but many of them were found to be highly hemolytic.^23^ More recently, antimicrobial QACs that are much less hemolytic were reported.^26^ Haldar and co-workers prepared cationic amphiphiles composed of chains of variable lengths bearing bis-ammonium cations that inhibited and eradicated biofilms of *S. aureus* and *Escherichia coli* strains without bactericidal or acute mammalian cell toxicity.^27,28^ Some of the compounds were even tested under *in vivo* conditions.^28−30^ It should be noted, however, that these compounds are also potent antimicrobials; thus, their antibiofilm activity likely results from the eradication of bacteria rather than direct intervention in the biofilm formation processes. As discussed by Melander and co-workers, there is a need for antibiofilm agents that have no effect on bacterial cell growth as such agents should not result in drug resistance.^31^ Lately, several examples of emergence of bacterial resistance against QACs were reported.^25,26^

The pillar[n]arene family was introduced more than a decade ago, and since then, there has been a rising interest in these new macrocycles.^32,33^ Pillar[n]arenes possess a unique set of properties, such as a symmetric tubular structure that can be easily functionalized at both rims with various functional groups.^32,33^ Their ease of synthesis and functionalization increase their popularity and have made them a widely used family of macrocycles with applications spanning different fields from biology to material sciences.^34−42^ Pillar[n]arenes are used as drug delivery systems,^35^ as separating agents,^37,38^ as light harvesting systems,^36,41^ as ions channels mimics,^36,42^ and as a scaffold for new materials such as supramolecular gels^34^ and polymers.^39^ In recent years, we showed that pillararene derivatives can be used to complex xenon in water^43^ and to form rim-to-rim supramolecular organogels^44^ as well as hydrogen bond-based supramolecular boxes in water.^45^

Recently, we found that cationic pillar[n]arenes (*n* = 5, 6) are potent inhibitors of biofilm formation of clinically important Gram-positive pathogens (Figure 1).^46,47^ Interestingly, although bearing several QACs, these cationic pillar[n]arenes show no effect on bacterial cell viability and cause no damage to red blood cells (RBCs) and no acute toxicity to human cells in culture at concentrations that are orders of magnitude higher than their antibiofilm active concentrations.^46,47^

**Figure 1:**
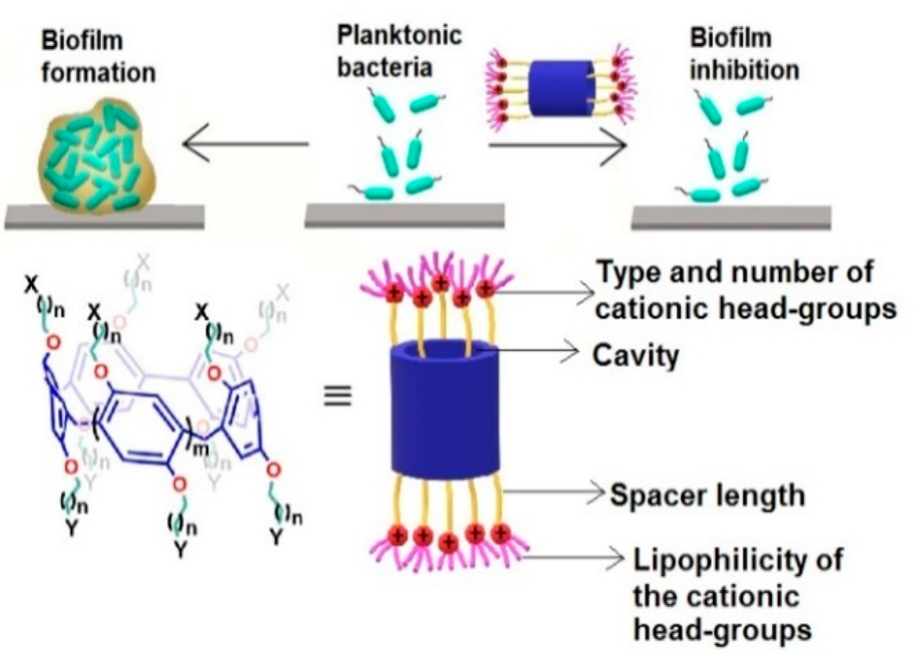
Biofilm formation and inhibition and the structural features tested during evaluation of the structure activity relationship of pillararene derivatives as inhibitors of biofilm formation.

Our previous studies suggested that the positive charges are the key for the observed antibiofilm activity.^46,47^ Very recently, Gao et al. demonstrated that a zwitterionic pillar[5]arene derivative has antimicrobial activity against the Gram-positive *S. aureus* (SH1000) and the Gram-negative *E. coli* (DH5a) strain.^48^ The zwitterionic pillar[5]arene derivative eradicated the pre-existing biofilm formed by an *E. coli* strain, albeit at high concentrations.^48^ In the present study, with the goal of establishing rules for the design of cationic pillararenes capable of inhibiting biofilm formation, we prepared and evaluated the antibiofilm activity of 16 cationic pillararene derivatives (15 new compounds and one reference). Therefore, it allowed us to establish a comprehensive structure activity relationship (SAR) of the pillararene derivatives that inhibit biofilm formation by clinically important Gram-positive pathogens.

## Results

### Synthesis of Cationic Pillararene Derivatives

To explore the effect of different structural characteristics of the cationic pillararenes on their antibiofilm activity, we designed and synthesized the cationic pillar[5,6]arene derivatives presented in Scheme 1. Compounds **1**–**14** were synthesized according to previously described procedures with some modifications.^46,47^ For synthetic procedures and characterization data, see Schemes S1–S3 and Figures S1–S51. Compound **15**, which is a water-soluble cationic pillar[5]arene-based rotaxane obtained from the previously synthesized cationic pillar[5]arene **1**, was prepared as shown in Scheme S4. Briefly, **15** was assembled by initially threading a dodecyl chain bearing one DABCO (1,4-diazabicyclo[2.2.2]octane) group and a bromide group (**15a**) into the cavity of pillar[5]arene **1** in water affording the pseudo rotaxane, which was then reacted with a second DABCO group to form the mechanically locked rotaxane **15**. The synthesis and the characterization of rotaxane **15** are presented in Scheme S4 and Figures S52–S55. To the best of our knowledge, this is the first example for the preparation of a polycationic pillar[n]arene-based rotaxane.^49^

**Scheme 1:**
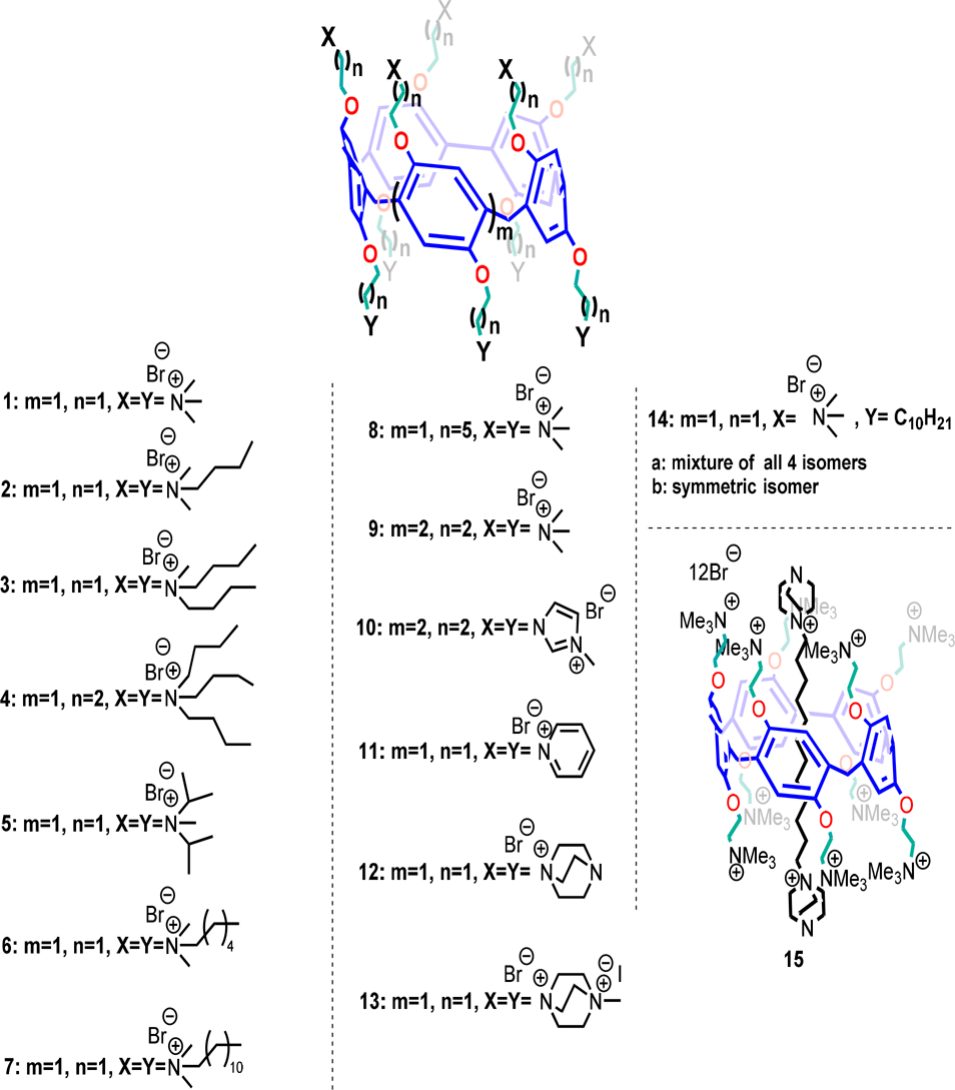
Cationic Pillar[5,6]arene Derivatives **1**–**15** Discussed in This Work

### Inhibition of Biofilm Formation

**Figure 2:**
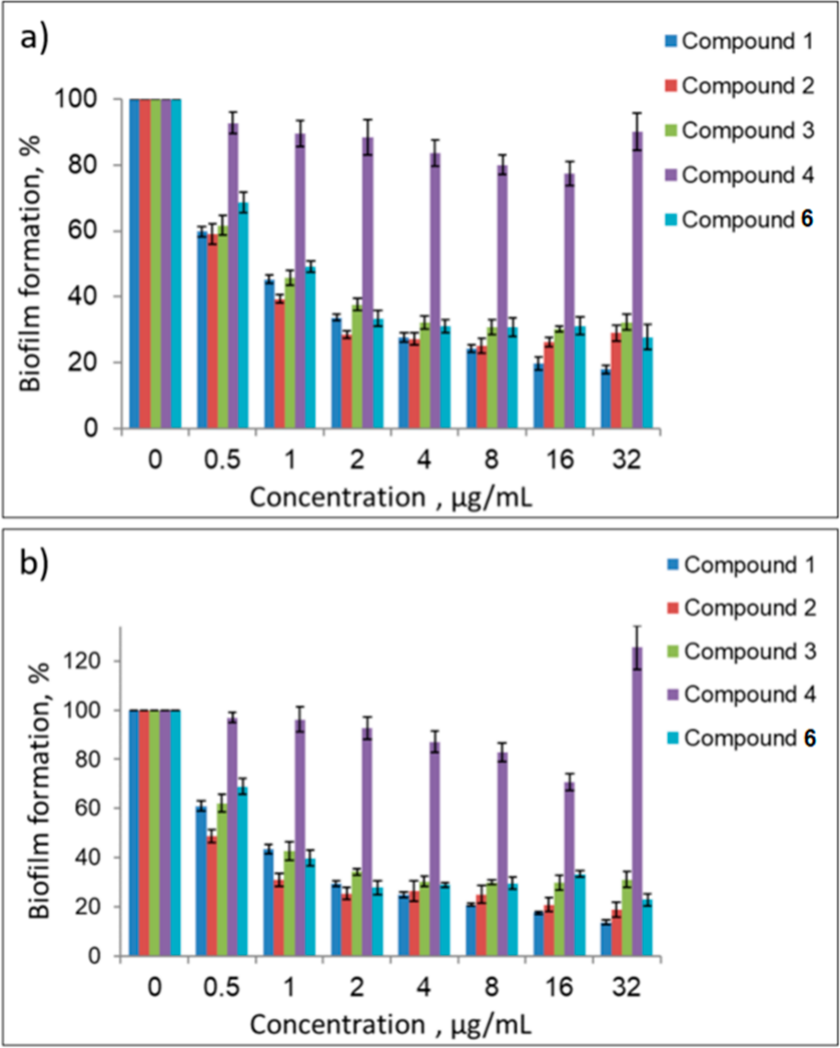
Biofilm formation by (a) *S. aureus* ATCC 33592 (MRSA) and (b) *E. faecalis* ATCC 29212 evaluated using the double dilution method (final OD_600_ = 0.1) in the presence of different concentrations of compounds **1**–**4** and **6**. Values are mean ± standard error of at least 3 independent experiments of 5 repetitions each.

To evaluate the ability of our cationic pillararenes to inhibit the formation of biofilm, we focused on two clinically important biofilm forming Gram-positive pathogens: methicillin-resistant *S. aureus* (ATCC 33592) and *E. faecalis* (ATCC 29212).^50−53^ We evaluated the entire dose response for all our compounds using the crystal violet protocol^46,47,54^ from which the MBIC_50_, i.e., the minimal concentration of a compound at which at least 50% inhibition of biofilm formation was observed compared to untreated cells, was computed. Images of the raw data are shown in Figures S56–S63. The quantitative analysis of the biofilm inhibition activity is presented in Figures 2 and S64–S67 ,and the extracted MBIC_50_ values are summarized in Table 1. Note that compounds **2**–**15** were evaluated for the first time, and compound **1**(46) was tested as a reference. With the exception of pillararenes **4**, **7**, **14a**, and **14b**, the cationic pillar[5,6]arene derivatives effectively inhibited the formation of biofilms by both *S. aureus* and *E. faecalis*. These results indicate that increasing the chain length of one or two of the substituents on the ammonium groups to butyl groups had a limited effect on the activity. However, when the hydrophobicity and size of three substituents of the cationic head groups were increased considerably (i.e., compounds **4** or **7**), the activity was fully abrogated. Compound **8**, with a longer spacer between the pillararene scaffold and the cationic head group, had an activity identical to **1**. Interestingly, alternation of the type of cationic head group (compare compounds **1**, **10**, **11**, and **12**) or an increase in the number of positively charged head groups (compare compounds **1** and **12** with compound **13**) had little effect on the inhibition of biofilm formation. Rotaxane **15** had similar to or slightly higher activity than **1**; both had MBIC_50_ values in the sub-micromolar range. Compound **14a**, which is a mixture of isomers, and compound **14b**, which is only the symmetric isomer, that have 5 positive charges, were about an order of magnitude less potent than the other compounds, which each have 10 or more positive charges.

**Table 1:** Biofilm Inhibitory Activity of Cationic Pillar[5,6]arene Derivatives and Predicted Octanol–Water Distribution Coefficients^a^

	MBIC_50_ values in μM (μg/mL)		
compound	*S. aureus* ATCC 33592	*E. faecalis* ATCC 29212	Log*D*
**1**	0.45 (1)	0.45 (1)	–32.55
**2**	0.37 (1)	0.19 (0.5)	–19.32
**3**	0.31 (1)	0.31 (1)	–6.08
**4**	>8.7 (>32)	>8.7 (>32)	7.16
**5**	0.61 (2)	0.61 (2)	–17.09
**6**	0.32 (1)	0.32 (1)	–10.42
**7**	>7.72 (>32)	>7.72 (>32)	16.25
**8**	0.35 (1)	0.35 (1)	–17.89
**9**	0.69 (2)	0.69 (2)	–38.35
**10**	0.63 (2)	0.63 (2)	–32.32
**11**	0.40 (1)	0.40 (1)	–24.71
**12**	0.71 (2)	0.36 (1)	–35.89
**13**	0.95 (4)	0.47 (2)	–77.42
**14a**	>14.94 (>32)	>14.94 (>32)	8.12
**14b**	14.94 (32)	7.47 (16)	8.12
**15**	0.35 (1)	0.18 (0.5)	–37.20

a Each MBIC_50_ value is a mean of at least three independent experiments, each including five replicates of each concentration.

### Hemolytic Activity

**Figure 3:**
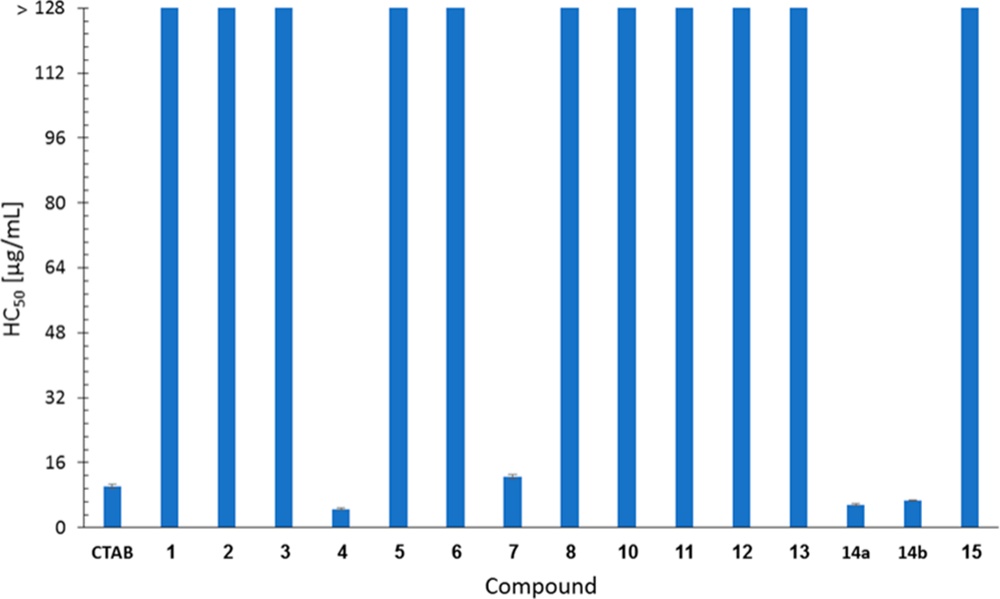
Hemolytic activities of cationic pillar[n]arenes **1**–**8** and **10**–**15**. Test compounds were added to rat RBCs suspended in PBS buffer; a range of concentrations was evaluated. After 1 h at 37 °C, the percentage of hemoglobin released relative to cells treated with Triton X100 (100% hemolysis) was quantified by measuring the absorbance at 550 nm. Each concentration was tested in triplicate, and the results are expressed as means ± standard error from two independent experiments.

The cationic amphiphilic nature of the pillararenes in this study suggests that these compounds could be potentially hemolytic. Therefore, the hemolytic effects of compounds **1**–**8** and **10**–**15** were evaluated in assays with rat RBCs. The positive controls were Triton X-100 and cetrimonium bromide (CTAB). The results are presented in Figure S68. The concentrations that caused 50% hemolysis of the RBCs (HC_50_ values) are summarized in Figure 3. Interestingly, all the active compounds tested, i.e., compounds **1**–**3**, **5**, **6**, **8**, **11**–**13**, and **15**, did not lyse red blood cells even at 256 μg/mL, the highest concentration tested, which is more than 100-fold higher than the MIBC_50_ values of these compounds. Compound **10** showed about 6% hemolysis at a concentration of 256 μg/mL. Notably, compounds **4**, **7**, **14a**, and **14b** were hemolytic with HC_50_ values similar to that of CTAB.

### Effect on Bacterial Growth

To confirm that the new derivatives do not affect bacterial growth, bacterial growth curve analysis was performed for 3 of the most potent compounds, i.e., compounds **2**, **12**, and **15**. The results are presented in Figures S70–S73. As expected and in line with previous observations,^46,47^ the compounds did not affect the bacterial growth even at the highest tested concentration, 64 μg/mL, which is significantly higher than the MBIC_50_.

## Discussion

The analysis of the antibiofilm activity of compounds **1**–**15** as well as compounds **16**–**21** reported previously (Table S1)^46,47^ revealed the structural features of cationic pillar[n]arenes that are important for the inhibition of biofilm formation by Gram-positive bacteria.

An increase in the hydrophobicity of the cationic pillar[n]arenes was accomplished by varying the aliphatic substituents on the ammonium head groups. Inhibition of biofilm formation by pillar[n]arenes **1**, **2**, **3**, **5**, **6**, and **16** was similar, whereas compounds **4** and **7** were inactive. These results indicate that an increase in the hydrophobicity had a small effect on the antibiofilm activity. The lack of activity of **4** was likely due to the inaccessibility of the positively charged head groups of the pillar[n]arenes as a result of the steric bulk caused by the aliphatic chains attached to the cationic center. Similarly, in cationic pillararene **7**, the C12-alkyl chains reduce the accessibility to the positively charged head groups. The predicted distribution coefficient (Log*D*) is a measure of hydrophobicity. Comparisons of the Log*D* values revealed that the four pillar[n]arenes that displayed poor inhibition of biofilm formation (**4**, **7**, **14a**, and **14b**) were also the most hydrophobic of the cationic pillar[n]arenes synthesized. The Log*D* values for **4**, **7**, **14a**, and **14b** are between 7.16 and 16.25 (Table 1); the rest of the cationic pillar[n]arenes were orders magnitude more hydrophilic (Log*D* value range of −6.08 to −77.42).

We also explored the importance of the length of the linker between the pillar[n]arene scaffold and the ammonium group. The similarities of inhibitory activities of cationic pillar[5]arenes **1**, **9**, **18**, and **8** suggest that changing the length of the spacer between the positively charged head groups and the pillararene scaffold from ethyl to propyl and to hexyl chain, respectively, did not significantly affect the inhibition of biofilm formation.

The chemical identity of the cationic head groups did not significantly affect the antibiofilm activity of the cationic pillar[n]arenes. For example, cationic pillar[5]arenes bearing ammonium and phosphonium and *N*-methyl imidazolium-, pyridinium- and DABCO-based cationic head groups (**1**, **20-21**, **10**, **11** and **12-13**, respectively) had MBIC_50_ values in the range of ∼0.4–1.55 μM.

Another feature addressed is the importance of the number of positive charges in determining the antibiofilm activity. An increase in the number of positive charges by 20% from 10 to 12 had a negligible effect on the antibiofilm activity (compound **17**).^46^ To test if a more dramatic increase in the number of positive charges affects antibiofilm activity, we generated a derivative with 20 positively charged head groups. *N*-Methylation of the DABCO head groups of cationic pillar[n]arene **12** with 10 positive charges gave derivative **13** with 20 positive charges. Interestingly, these two cationic pillar[n]arenes had a similar antibiofilm activity. These results suggest that, beyond a certain number of charges, the clustering of the charges on the pillararene scaffold rather than the number of charges per se, affects the antibiofilm activity. Earlier results indicated that the positive head groups should be clustered together and the monomeric units of the respective pillararenes, even at 5 times the concentrations used, were completely inactive.^47^

To challenge if reducing the number of positive head groups and placing them either on one or on both sides of the pillararene scaffold affects the antibiofilm activity, we tested the antibiofilm activity of **14a**, the statistical mixture of all pillar[5]arene isomers having five positive head groups, and compared it to the activity of **14b**, the symmetric isomer. Table 1 shows that both **14a** and **14b** were much less active. The fact that mixture **14a** was even less active than **14b** suggests that one cannot rule out that some of the decrease in the observed activity occurred because these systems have only 5 positive head groups. Importantly, one should also note that the Log*D* of **14a**,**b** is in fact very different from all compounds found to be potent inhibitors of biofilm formation. Thus, it may well be that the reduction in the potency of these materials arises from their high lipophilicity.

Finally, we explored the importance of one of the unique features of the pillar[n]arene scaffold, which is the cavity. We therefore synthesized the water-soluble cationic pillar[5]arene-based rotaxane **15**. Pillar[n]arenes can serve as good building blocks for rotaxanes and pseudorotaxanes due to their symmetrical rigid structure, π-rich cavity, and host–guest properties.^17^ In constructing rotaxane **15**, we used cationic pillar[5]arene **1** as the wheel and a dodecyl chain with two DABCO groups on both ends as the dumbbell. The blockage of the cavity of the pillar[5]arene did not reduce the ability of the pillar[n]arene to inhibit the formation of the biofilms. MBIC_50_ values of rotaxane **15** were of the same order of magnitude as those of pillar[5]arene **1** from which it was generated, although the values of the former were slightly lower.

The evaluation of the antibiofilm activity of the collection of cationic pillar[n]arenes **1**–**21** revealed several structure–activity relationship features important for potent inhibition of biofilm formation by the cationic pillar[n]arenes: (1) *accessibility of the positive charges* (significant shielding of the positive charges reduced the activity of the cationic pillararenes); (2) *the pillar[n]arene structure* (the cavity of the pillar[n]arene is not essential for the inhibition of biofilm formation; however, clustering of the positive charges, which is dictated by the pillar[n]arene structure, is key for antibiofilm activity); (3) *lipophilicity* (some enhancement of lipophilicity due to the alteration of the substituents on the positively charged head groups is tolerated); (4) *spacer* (the distance between the pillar[n]arene macrocycle and the positive head groups can be modified through alteration of the aliphatic chain spacer); (5) *cationic head group* (the type of the positive head group does not affect the inhibition of biofilm formation); (6) *net positive charge* (variation from 10 to 20 positive head groups does not affect the activity of the cationic pillar[n]arenes; however, a reduction of this number to 5 positive charges while placing the lipophilic group in other positions decreases the activity by an order of magnitude); (7) Log*D* values (compounds having positive Log*D* values were found to be inactive).

## Conclusion

In this study, we identified the structural determinants that affect the efficacy of cationic pillar[n]arenes in inhibiting biofilm formation by two important Gram-positive pathogens. Many of the tested compounds potently inhibited biofilm formation, and some were completely inactive. Importantly, we found that a plurality of accessible positive charges and not their nature are important determinants for the observed antibiofilm activity of these compounds. We showed that the multiplication of the number of positive charges from 10 to 20 did not increase the activity; however, a reduction in the number of positive head groups combined with an increase in the lipophilicity of the compound decreased the antibiofilm activity of the cationic pillararenes. Importantly, we provided evidence that the cavity of the pillararene that can serve as a host for small molecules and aliphatic chains is not essential for the inhibition of biofilm formation. However, the clustering of the positive charges on the pillararene skeleton is important. Interestingly, the compounds that are potent inhibitors of biofilm formation were also found to be nonhemolytic and to have no effect on bacterial cell growth. Therefore, it will be interesting to study if these compounds keep their biofilm inhibition activity for a longer period compared to other agents. In addition, it will be important to test if any synergistic effect can be observed when such cationic pillararene agents are administered along with known antibiotics in drug-resistant bacterial strains. These findings and the conclusions drawn thereof will guide the design of more potent and active pillararene-based materials for the inhibition of biofilms by Gram positive bacteria.

## Methods

### Materials

Starting materials were purchased from Sigma-Aldrich, Alfa Aesar, TCI, Cambridge Isotope Laboratories, and Bio-Lab Ltd. and used as received. Chemical reactions were monitored by TLC (Merck, silica gel 60 F254), and the compounds were purified by SiO_2_ flash chromatography (Merck Kieselgel 60). ^1^H and ^13^C NMR spectra were recorded on 400 and 500 MHz Bruker Avance NMR spectrometers. All compounds were prepared according to Schemes S1–S4 using a modified procedure,^46,47^ and their full NMR and HRMS characterization appear in Schemes S1–S4 and Figures S1–S55. The purity of the compounds was determined by chemical analysis or HPLC and was higher than 95% (see the Supporting Information).

### Biological Assays

#### Analysis of Biofilm Inhibition

The antibiofilm activity assay was performed as described previously^46,47,54^ with minor modifications. Briefly, the tested bacterial strains were grown from frozen stocks in brain heart infusion (BHI) medium overnight at 37 °C in 5% CO_2_. Then, 100 μL of serial 1:2 dilutions of each compound in Tryptic soy broth (TSB) + 1% glucose (32, 16, 8, 4, 2, 1, and 0.5 μg/mL) were prepared in flat-bottomed 96-well microplates (Costar, Corning). Control wells with no compounds and wells without bacteria containing each tested concentration of the compounds (blanks) were also prepared. An equal volume (100 μL) of bacterial suspensions in TSB + 1% glucose was added to each well to a final OD_600_ of 0.1. After incubation for 24 h at 37 °C in 5% CO_2_ under aerobic conditions, spent media and free-floating bacteria were removed by turning over the plates. The wells were vigorously rinsed at least four times with doubly distilled water (DDW).

#### Crystal Violet Assay

0.4% Crystal violet (200 μL) solution was added to each well. After 45 min, wells were vigorously rinsed three times with DDW to remove unbound dye. After adding 200 μL of 33% acetic acid to each well, the plate was shaken for 15 min to release the dye. Biofilm formation was quantified by measuring the difference between the absorbance of untreated and treated bacterial samples for each tested concentration of the compounds and the absorbance of the appropriate blank well at 600 nm (*A*_600_) using a Tecan plate reader. The MBIC_50_ was defined as the lowest concentration at which at least 50% reduction in biofilm formation was measured compared to untreated cells. Each concentration of compound was tested in five replicates, and at least three independent experiments were performed.

#### Rat Red Blood Cell Hemolysis Assay

The hemolysis was performed as previously described with minor modifications.^55^ Briefly, a sample of rat red blood cells (2% w/w in PBS) was incubated with each of the tested compounds (CTAB and compounds **1**–**8** and **10**–**15**) for 1 h at 37 °C in 5% CO_2_ using the double dilution method starting at a concentration of 256 μg/mL. The negative control was PBS, and the positive control was a 1% v/v solution of Triton X-100 (which induced 100% hemolysis). Following centrifugation (2000 rpm, 10 min, ambient temperature), the supernatant was removed and absorbance at 550 nm was measured using a microplate reader (Genios, TECAN). The graphs of the percentage of hemoglobin released vs the compounds’ concentrations, relative to the positive control (Triton X-100), were obtained from two independent experiments performed in triplicate.

#### Bacterial Growth Curve Analysis

The tested bacterial strains were first grown from the frozen stock in BHI broth for 24 h at 37 °C. Volumes of 100 μL of serial 1:2 dilutions (64, 32, 16, 8, 4, 2, and 1 μg/mL) of the selected compounds in TSB + 1% glucose were prepared in flat-bottomed 96-well microplates (Corning). Next, an equal volume (100 μL) of bacterial suspension in TSB + 1% glucose was added to each well to a final OD_600_ of 0.01. Control wells with no compounds and wells without bacteria (blanks) were also prepared. During a 24 h incubation at 37 °C, growth kinetics were monitored by recording the optical density at a wavelength of 600 nm (OD_600_) using a Tecan plate reader. Each concentration was tested in triplicate, and the results are shown as an average of two independent experiments.

## Supporting Information Available

Synthetic procedures and spectroscopic characterization, including ^1^H and ^13^C NMR spectra of all compounds used in the study as well as descriptions of the biological tests and the results obtained for compounds **1**–**15** (PDF)

## Abbreviations

CTAB: cetrimonium bromide
DABCO: 1,4-diazabicyclo-[2.2.2]octane
HC_50_: concentration that results in 50% lysis of RBCs
MBIC_50_: the minimal concentration of a compound at which at least 50% inhibition of biofilm formation was observed compared to untreated cells
NMR: nuclear magnetic resonance
RBCs: red blood cells
HPLC: high pressure liquid chromatography
