# Supplementary material for: Design Guidelines for Cationic Pillar[n]arenes that Prevent Biofilm Formation by Gram-Positive Pathogens
Source: ACS Infect Dis. 2021 Mar 4;7(3):579–85. doi: 10.1021/acsinfecdis.0c00662 (PMC8041275; doi:10.1021/acsinfecdis.0c00662)
Supplement: Supplementary file 1 — id0c00662_si_001.pdf [file id0c00662_si_001.pdf]

Supporting information

Design Guidelines for Cationic Pillar[n]arenes that Prevent Biofilm Formation by Gram-Positive Pathogens

Dana Kaizerman-Kane,[a]† Maya Hadar[a]†, Roymon Joseph[a, b], Dana Logviniuk[a], Yossi Zafrani[a,c], Micha Fridman[a] and Yoram Cohen*[a]

[a] School of Chemistry, Sackler Faculty of Exact Sciences, Tel Aviv University, Tel Aviv, 69978, Israel,

[b] Department of Chemistry, University of Calicut, Calicut 673635, Kerala, India,

[c] Department of Organic Chemistry, Israel Institute for Biological Research, Ness-Ziona 74000, Israel

*Corresponding Author: ycohen@tauex.tau.ac.il

Table of Contents

1. **General methods** **S2**
2. **Synthesis and characterization of cationic water soluble pillar[5,6]arenes** **S3**
3. **Results of selected examples from previous work.** **S41**
4. **Biological assays- Crystal violet assay, RBCs hemolysis and effect on bacterial Growth** **S42**
5. **Analytical HPLC chromatograms** **S57**
6. **References** **S57**

1. General methods

Starting materials were purchased from Sigma-Aldrich, Alfa Aesar, TCI, Cambridge Isotope Laboratories, and Bio-Lab Ltd and used as received. Chemical reactions were monitored by TLC (Merck, silica gel 60 F254) and the compounds were purified by SiO_2 flash chromatography (Merck Kieselgel 60). ^{1}H- and ^{13}C-NMR spectra were recorded on 400 and 500 MHz Bruker Avance NMR spectrometers. Chemical shifts (δ) are given in part per millions (ppm), and spin-spin coupling (J) in Hz. The chemical shifts are quoted relative to residual HDO signal (at δ 4.80 ppm for the ^{1}H-NMR) when the solvent is D_2O, to residual $CHCl_3$ signal (at δ 7.26 ppm for the ^{1}H-NMR and 77.2 ppm for the ^{13}C-NMR) when the solvent is $CDCl_3$, to residual DMSO signal (at δ 2.50 ppm for the ^{1}H-NMR and 39.5 ppm for the ^{13}C-NMR) when the solvent is DMSO-d_6, to residual CH_3CN signal (at δ 1.94 ppm for the ^{1}H-NMR and 1.32, 118.26 ppm for the ^{13}C-NMR) when the solvent is CD_3CN, or to residual MeOH signal (at δ 3.31 ppm for the ^{1}H-NMR and 49.0 ppm for the ^{13}C-NMR) when the solvent is methanol-d_4. Abbreviations for multiplicities used: s = singlet, d = doublet, t = triplet, q = quartet, quint = quintet, m = multiplet, br = broad signal. Determination of C, H, and N compositions were performed using a Perkin-Elmer 2400 series II Analyzer. High-resolution electrospray and APPI mass-spectra were recorded on a Waters Synapt instrument. Reverse-phase high pressure liquid chromatography (RP-HPLC): C18 5u, 250x4.6mm, eluent given in parentheses.

LogD values were calculated using MarvinSketch software (version 6.3.1) with default parameters. Electrolyte concentrations: 0.1 M Cl-, Na+, pH 7.4.

2. Synthesis and characterization of cationic water soluble pillar[5,6]arenes

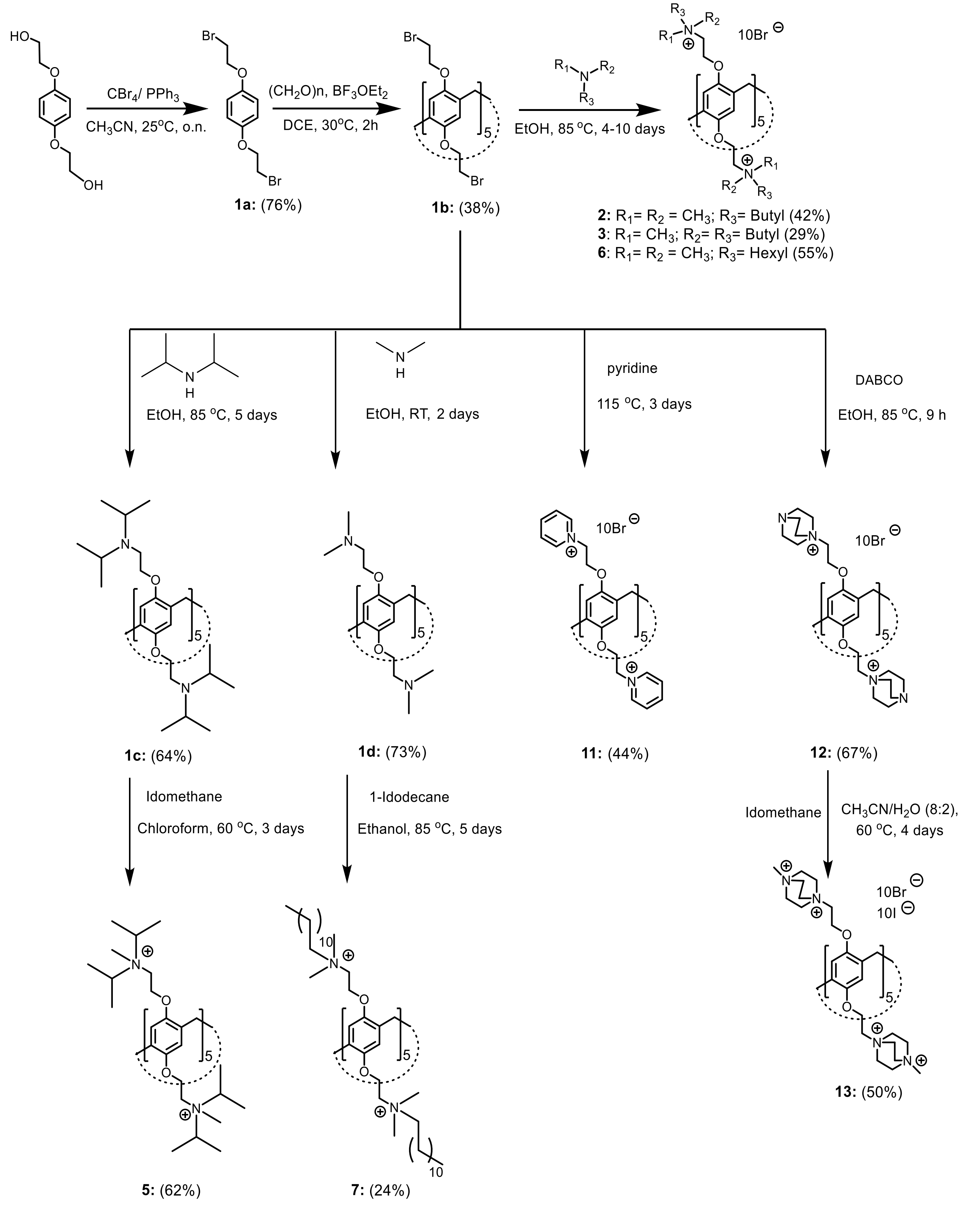

Scheme S1. Synthesis of compounds **2**, **3**, **5-7**, **11-13.**

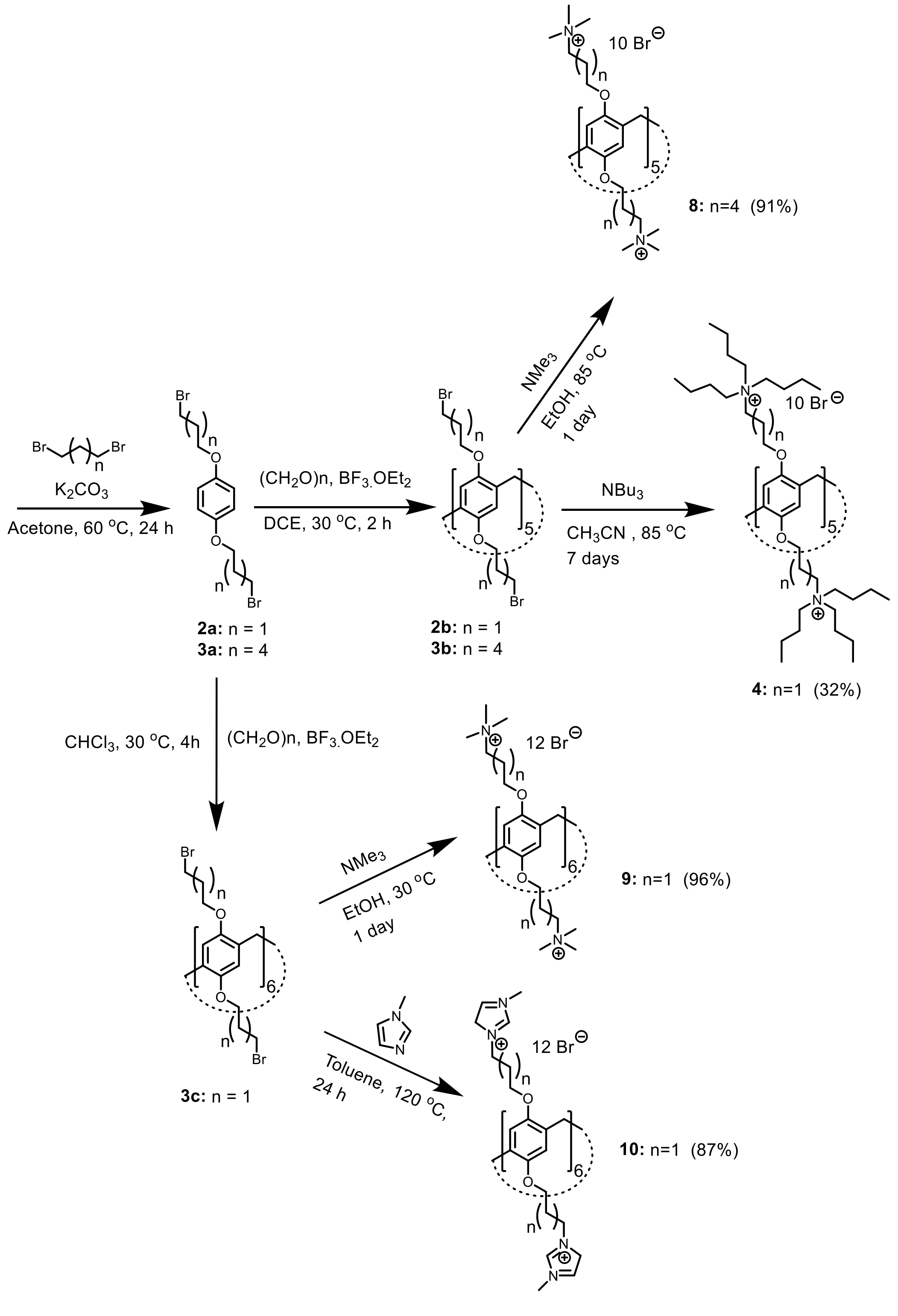

Scheme S2. Synthesis of compounds **4**, **8-10.**

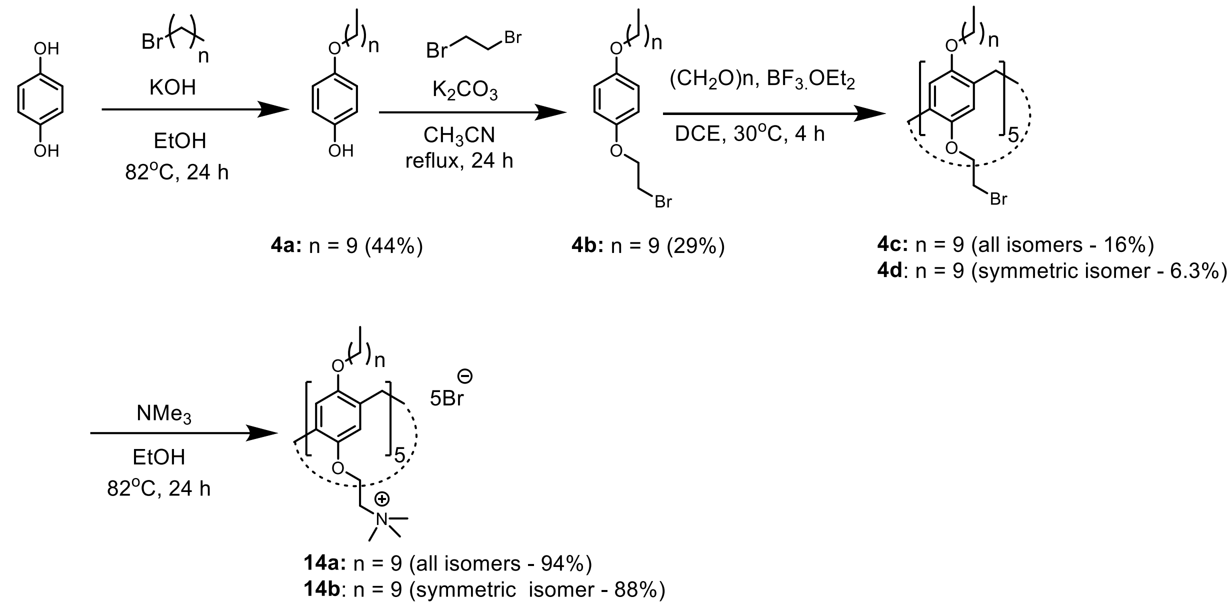

Scheme S3. Synthesis of compounds **14a, 14b.**

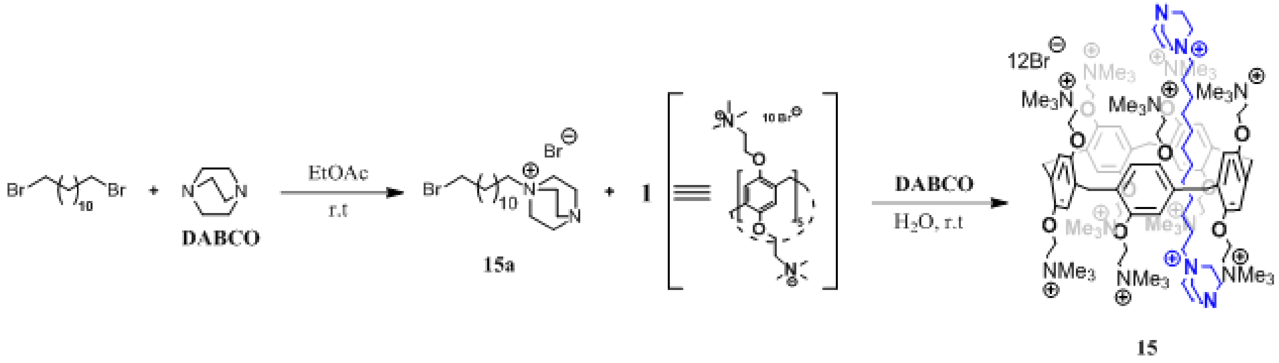

Scheme S4. Synthesis of compound **15.**

All compounds were thoroughly purified and analyzed by NMR. NMR data of the known compounds was consistent with literature. Full NMR and HRMS analyses for all new compounds are reported below.

Synthesis of 1b. Compound **1b** was synthesized and characterized according to the reported procedure[1]. ^{1}H NMR (400 MHz, $CDCl_3$): δ 6.91 (s, ArH, 10H), 4.23 (t, J = 5.7 Hz, $ArOCH_2CH_2Br$, 20H), 3.85 (s, $ArCH_2Ar$, 10H), 3.64 (t, J = 5.7 Hz, $ArOCH_2CH_2Br$, 20H) ppm. ^{13}C NMR (100 MHz, $CDCl_3$): δ 149.8, 129.2, 116.2, 69.1, 30.9, 29.5 ppm.

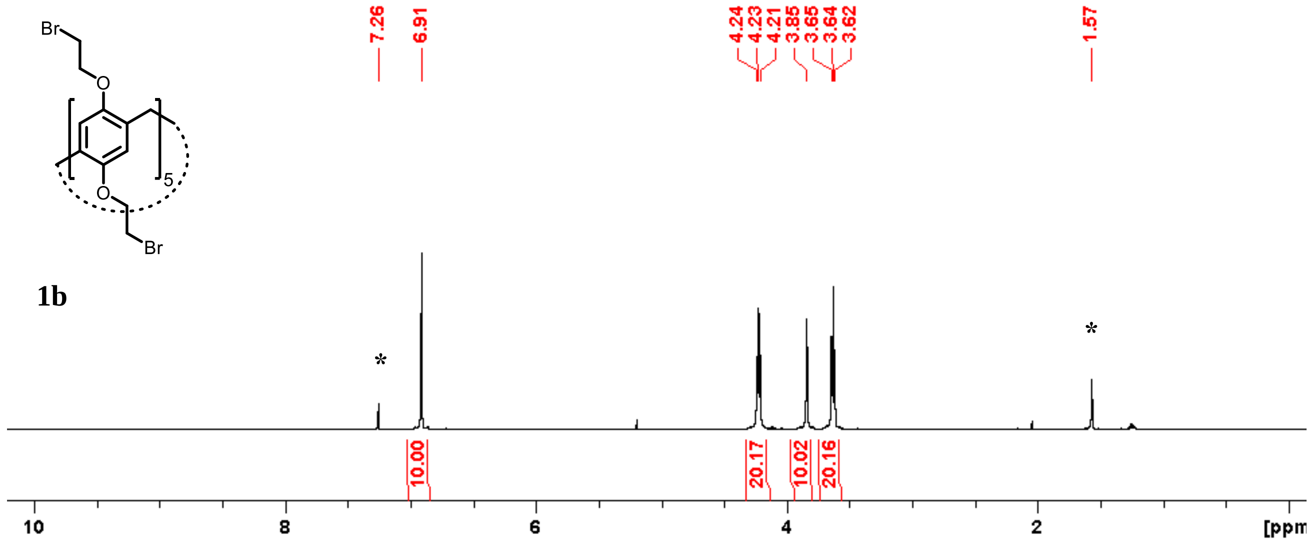

Figure S1. ^{1}H-NMR spectrum of **1b** in $CDCl_3$ (400 MHz). (*) Represents solvent peaks.

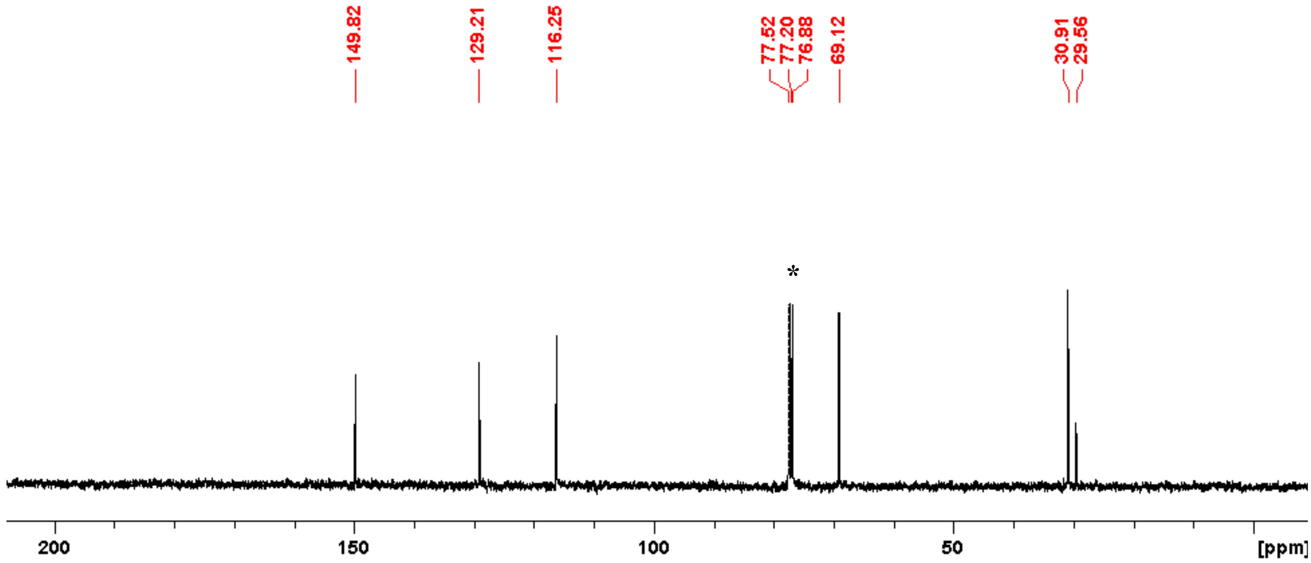

Figure S2. ^{13}C-NMR spectrum of **1b** in $CDCl_3$ (100 MHz). (*) Represents solvent peaks.

Synthesis of 1c. Diisopropylamine (5.8 mL, 41.4 mmol) was added to a solution of decabromoethylpillar[5]arene (**1b**) (580 mg, 0.35 mmol) in ethanol (5 mL) under vigorous stirring. The mixture was refluxed for 5 days. After cooling to 25°C, the solvent was evaporated to obtain a yellowish powder. A saturated $K_2CO_{3(aq)}$ (10 mL) was then added and the turbid mixture was stirred for 1h. The product was extracted with ethyl acetate (3 × 10ml), and the organic phase was evaporated to give yellow oil. The product was purified by column chromatography (silica gel; 10% NH_4OH in methanol: chloroform) to afford **1c** as a yellow oil (0.5 g, 64%). ^{1}H-NMR (400 MHz, $CDCl_3$): δ 6.87 (s, Ar*H*, 10H), 3.98 (m, ArO*CH*HCH$_2$, 10H), 3.76 (s, ArC*H*$_2$Ar, 10H), 3.72 (m, ArOCH*H*CH$_2$, 10H), 3.09 (qui, *J*=8 Hz, ArOCH$_2$*CH*$_2$N, 20H), 2.90 (m, N*CH*(CH$_3$)$_2$, 20H), 1.06 (d, *J*=4 Hz, NCH(*CH*$_3$)$_2$, 60H), 1.05 (d, *J*=4 Hz, NCH(*CH*$_3$)$_2$, 60H) ppm. ^{13}C-NMR (100 MHz, $CDCl_3$): δ 150.0, 128.5, 115.2, 70.3, 52.3, 49.4, 29.3, 21.2 ppm. HRMS: m/z Calcd. for $C_{115}H_{201}O_{10}N_{10}$ $[M+H]^+$ 1882.5527, found 1882.5533.

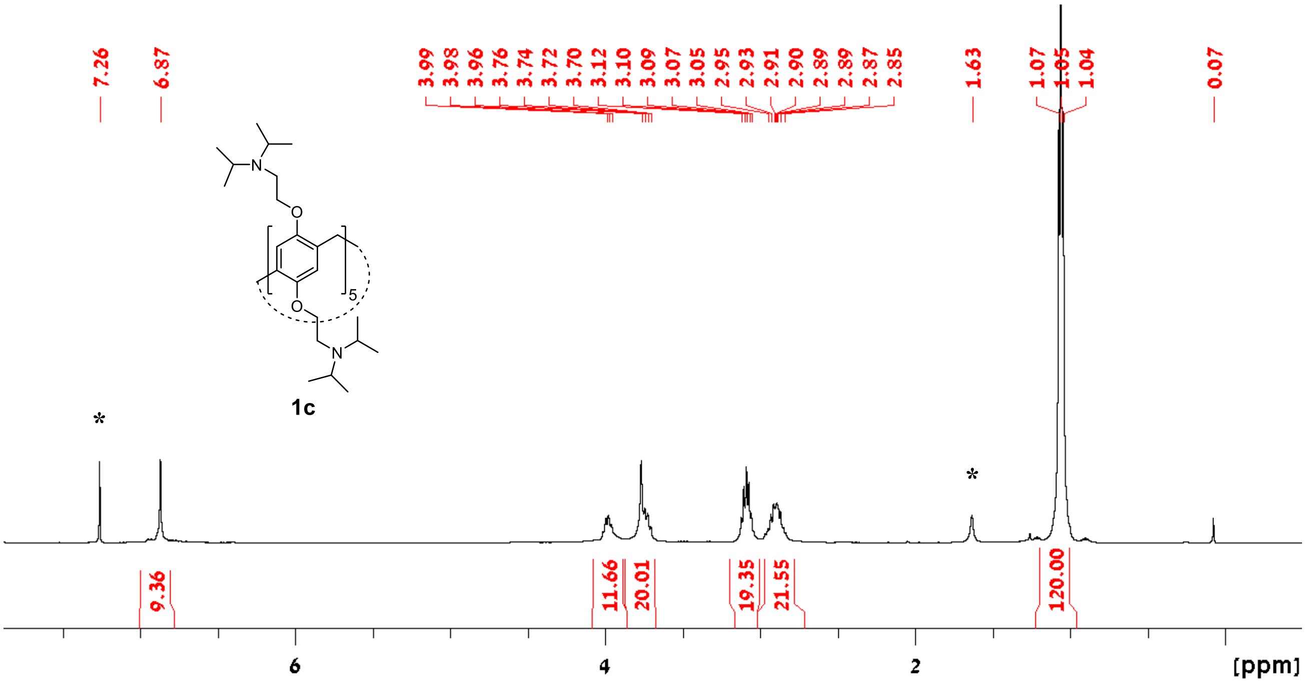

Figure S3. ^{1}H NMR spectrum of **1c** in $CDCl_3$ (400 MHz). (*) Represents solvent peaks.

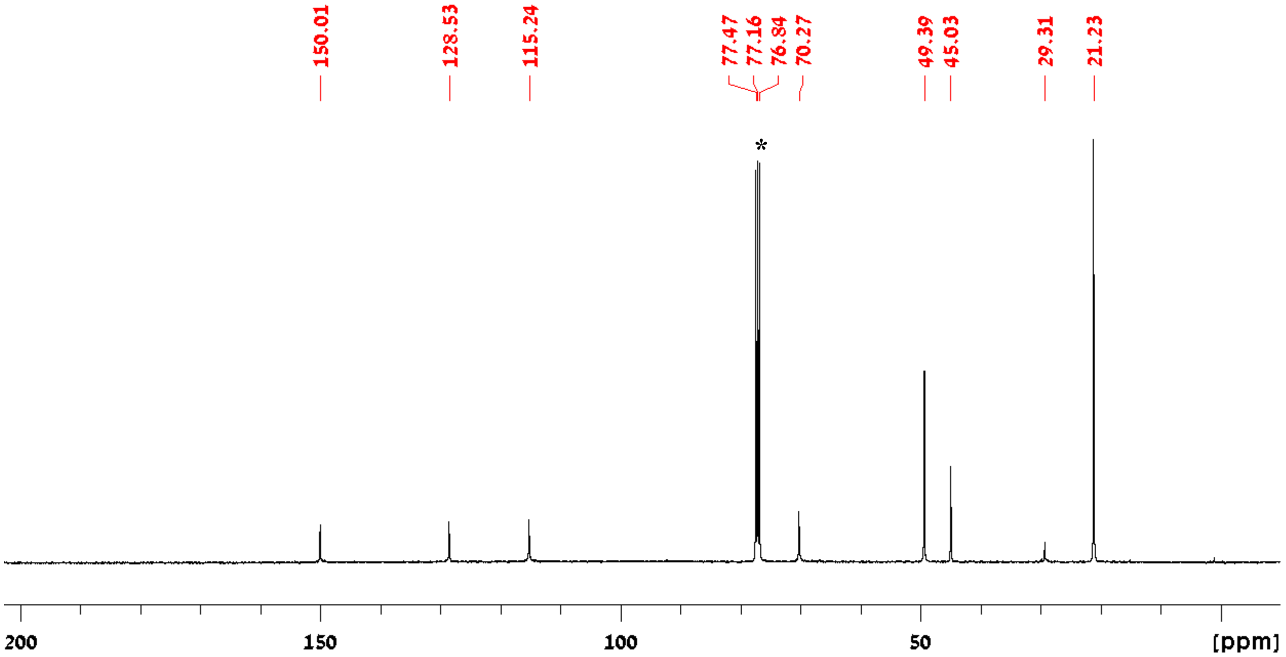

Figure S4. ^{13}C NMR spectrum of **1c** in $CDCl_3$ (100 MHz). (*) Represents solvent peaks.

Synthesis of 1d. Compound **1d** was synthesized and characterized according to the reported procedure.[8] ^{1}H-NMR (400 MHz, $CDCl_3$): δ 6.85 (s, ArH, 10H), 3.99 (brs, ArOCH_2CH$_2$N, 20H), 3.75 (s, ArCH_2Ar, 10H), 2.73 (t, J = 5.7 Hz ArOCH$_2\mathit{CH}_2$N, 20H), 2.34 (s, N(CH_3)$_2$, 60H) ppm.

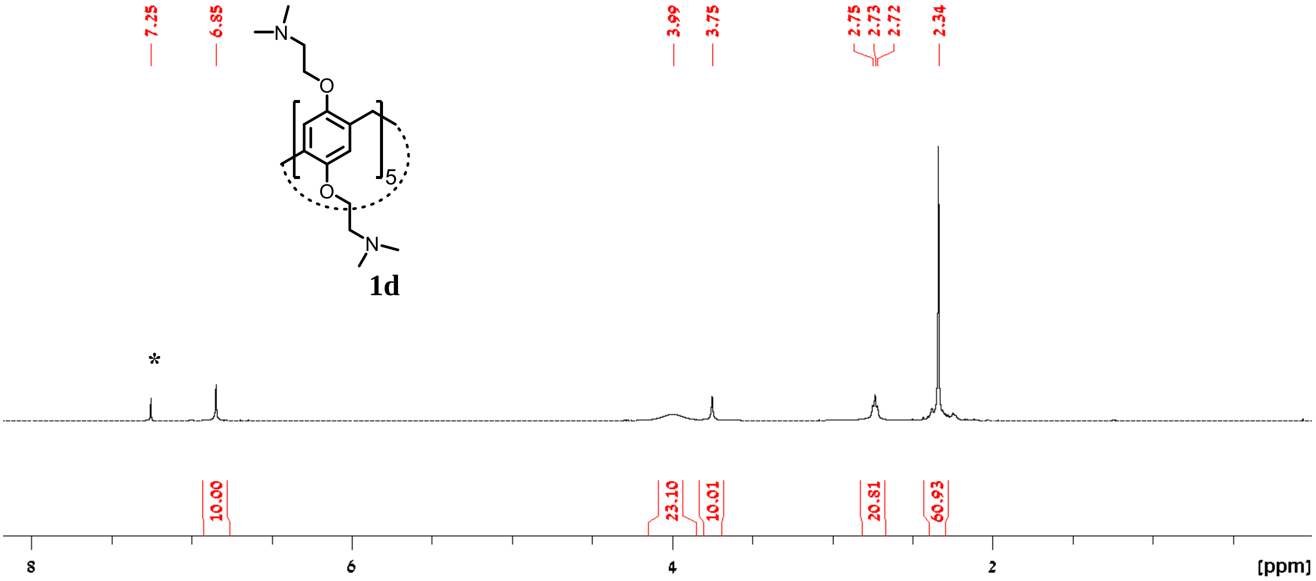

Figure S5. ^{1}H-NMR spectrum of **1d** in $CDCl_3$ (400 MHz). (*) Represents solvent peaks.

Synthesis of 2a. Compound **2a** was synthesized and characterized according to the reported procedure[2]. ^{1}H-NMR (400 MHz, $CDCl_3$): δ 6.84 (s, Ar*H*, 4H), 4.05 (t, *J* = 5.9 Hz, ArOC*H*$_2$CH$_2$CH$_2$Br, 4H), 3.60 (t, *J* = 6.7 Hz, ArOCH$_2$CH$_2$C*H*$_2$Br, 4H), 2.29 (m, ArOCH$_2$C*H*$_2$CH$_2$Br, 4H) ppm. ^{13}C-NMR (100 MHz, $CDCl_3$): δ 153.0, 115.5, 65.9, 32.5, 30.2 ppm.

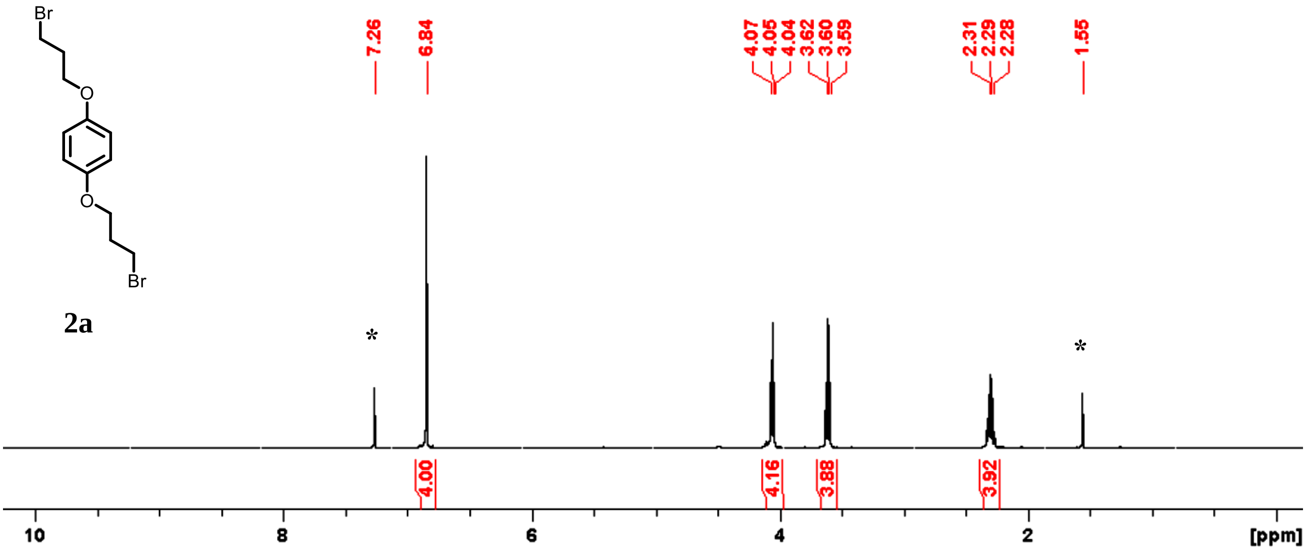

Figure S6. ^{1}H-NMR spectrum of **2a** in $CDCl_3$ (400 MHz). (*) Represents solvent peaks.

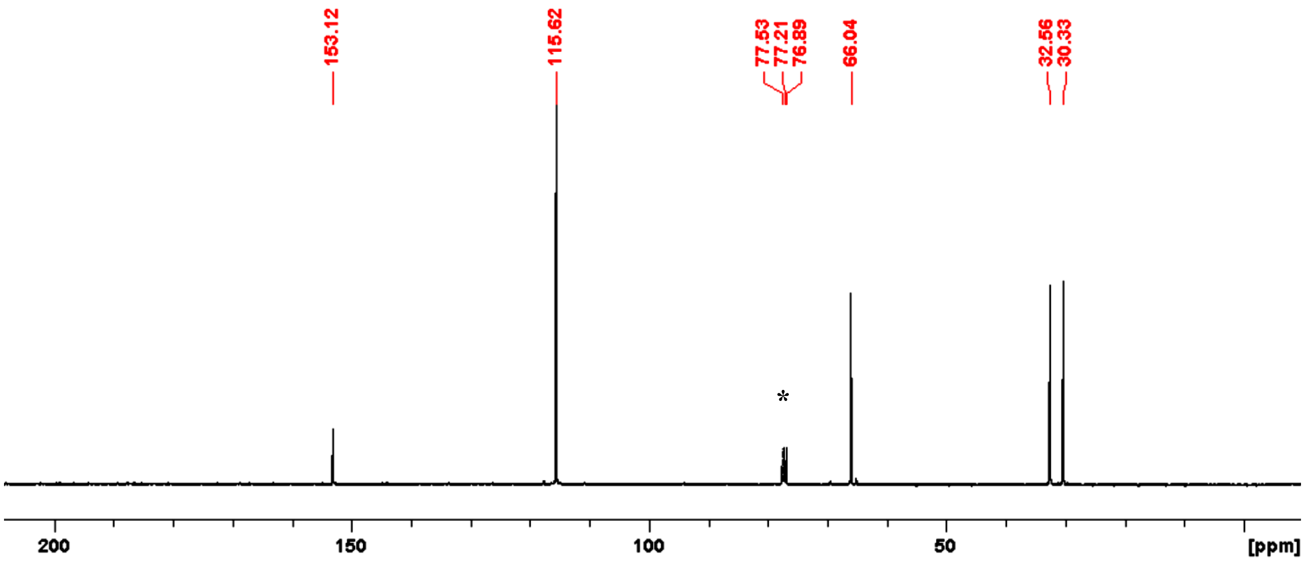

Figure S7. ^{13}C-NMR spectrum of **2a** in $CDCl_3$ (100 MHz). (*) Represents solvent peaks.

Synthesis of 3a. Compound **3a** was synthesized and characterized according to the reported procedure[3]. Yield (2.4 g, 18%). ^{1}H NMR (500 MHz, $CDCl_3$): δ 6.82 (s, Ar*H*, 4H), 3.91 (t, *J* = 5.0 Hz, ArOC*H*$_2$, 4H), 3.42 (t, *J* = 5.0 Hz, ArBrC*H*$_2$, 4H), 1.89 (quin, *J* = 7.0 Hz, $OCH_2CH_2(CH_2)_4Br$, 4H), 1.77 (quin, *J* = 6.8 Hz, $BrCH_2CH_2(CH_2)_4O$, 4H), 1.50 (m, $O(CH_2)_2CH_2CH_2(CH_2)_2Br$, 8H) ppm. ^{13}C-NMR (100 MHz, $CDCl_3$): δ 153.3, 115.5, 68.5, 34.0, 32.8, 29.3, 28.1, 25.4 ppm.

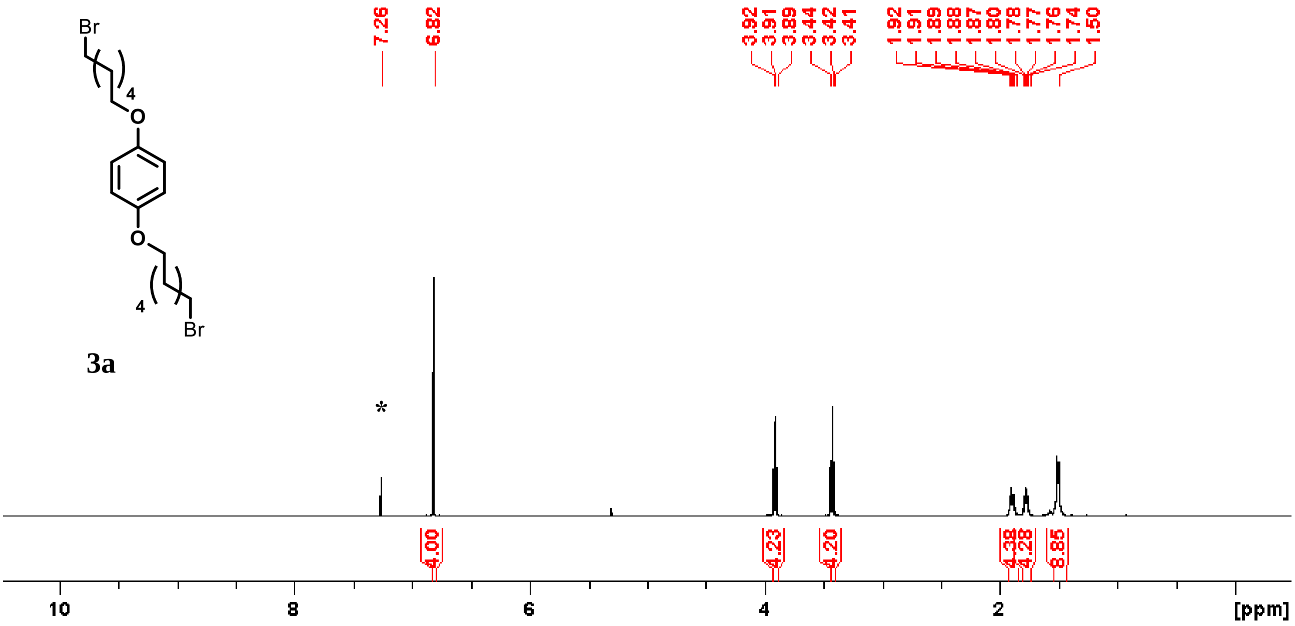

Figure S8. ^{1}H-NMR spectrum of **3a** in $CDCl_3$ (500 MHz). (*) Represents solvent peaks.

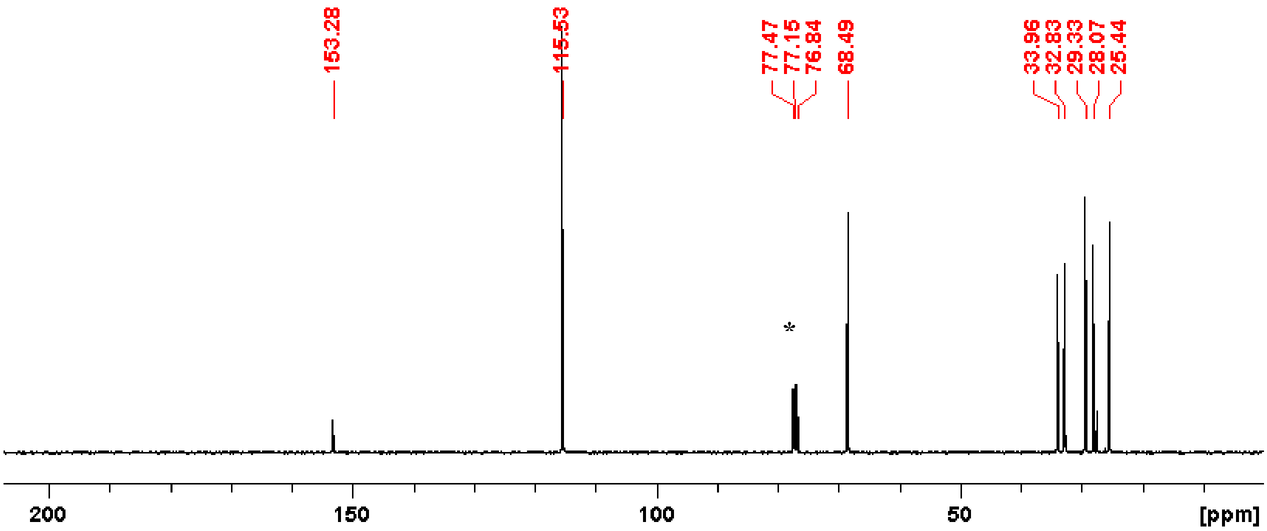

Figure S9. ^{13}C-NMR spectrum of **3a** in $CDCl_3$ (100 MHz). (*) Represents solvent peaks.

Synthesis of 2b. Compound **2b** was synthesized and characterized according to the reported procedure[2]. ^{1}H-NMR (400 MHz, $CDCl_3$): δ 6.74 (s, Ar*H*, 10H), 3.99 (t, *J* = 6.3 Hz, $ArOCH_2CH_2CH_2Br$, 20H), 3.75 (s, $ArCH_2Ar$, 10H), 3.52 (t, *J* = 6.5 Hz, $ArOCH_2CH_2CH_2Br$, 20H), 2.21 (m, $ArOCH_2CH_2CH_2Br$, 20H) ppm. ^{13}C NMR (100 MHz, $CDCl_3$): δ 149.8, 128.5, 115.3, 66.3, 32.7, 30.5, 29.9 ppm.

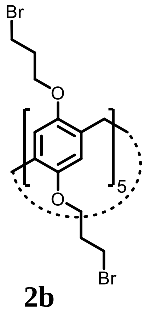

2b

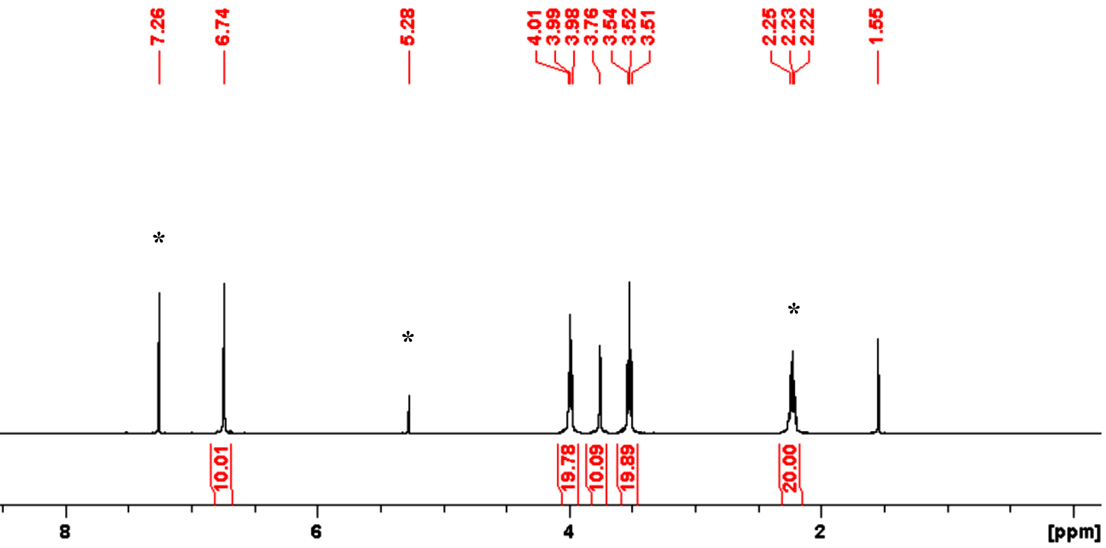

Figure S10. ^{1}H-NMR spectrum of **2b** in $CDCl_3$ (400 MHz). (*) Represents solvent peaks.

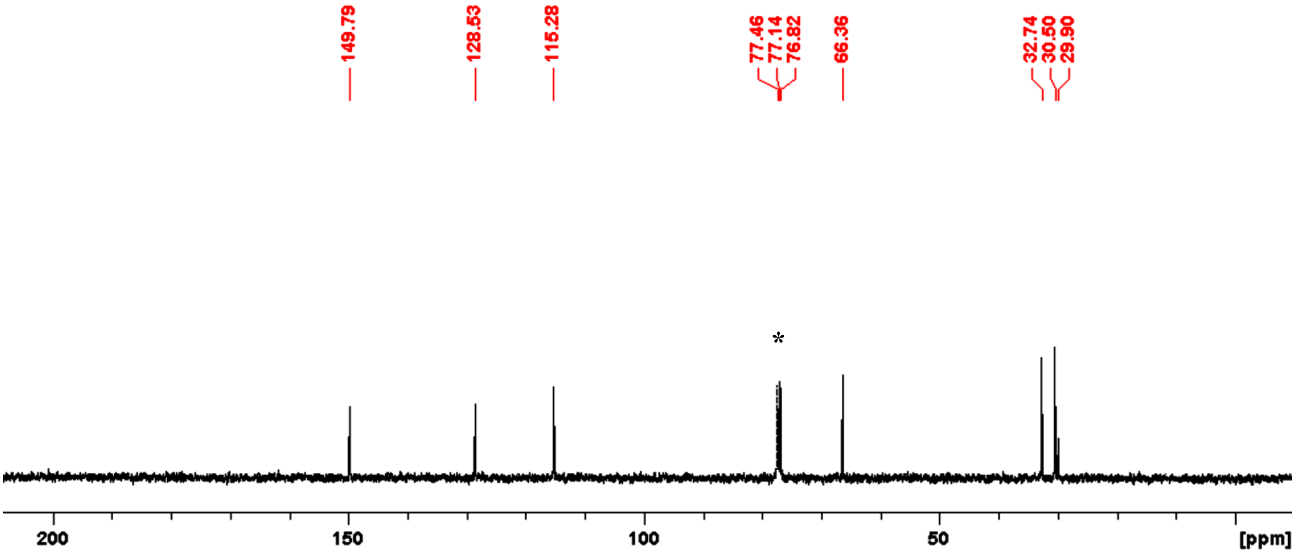

Figure S11. ^{13}C-NMR spectrum of **2b** in $CDCl_3$ (100 MHz). (*) Represents solvent peaks.

Synthesis of 3b. To a solution of **3a** (2.4 g, 5.5 mmol) in 1,2-dichloroethane (0.13 L) was added paraformaldehyde (0.225 g, 6.6 mmol) followed by addition of BF_3·OEt_2 (0.94 g, 6.6 mmol), while flask is in ice bath. The reaction mixture was kept at 25 °C for 3 h under argon atmosphere. Water (50 mL) was added, and mixture stirred for 30 min. The reaction mixture was washed with water (2 × 50 mL), brine (2 × 50 mL) and dried with sodium sulfate and concentrated *in vacuo*. The product was purified by chromatography (silica gel; petroleum ether:ethyl acetate) to afford **3b** as a white yellow solid (0.86 g, 35%). ^{1}H-NMR (400 MHz, $CDCl_3$): δ 6.84 (s, Ar*H*, 10H), 3.89 (br, ArOCH_2, 20H), 3.75 (s, ArCH_2Ar, 10H), 3.22 (br, BrCH_2CH$_2$(CH$_2$)$_4$, 20H), 1.79 (br, OCH$_2$CH_2(CH$_2$)$_4$, 20H), 1.65 (br, BrCH$_2$CH_2(CH$_2$)$_4$, 20H), 1.43 (br, O(CH$_2$)$_2$CH_2(CH$_2$)$_3$, 20H) , 1.33 (br, Br(CH$_2$)$_2$CH_2(CH$_2$)$_3$, 20H) ppm. ^{13}C-NMR (125 MHz, $CDCl_3$): δ 149.7, 128.2, 114.7, 68.1, 33.9, 32.6, 29.8, 29.5, 28.2, 25.5 ppm.

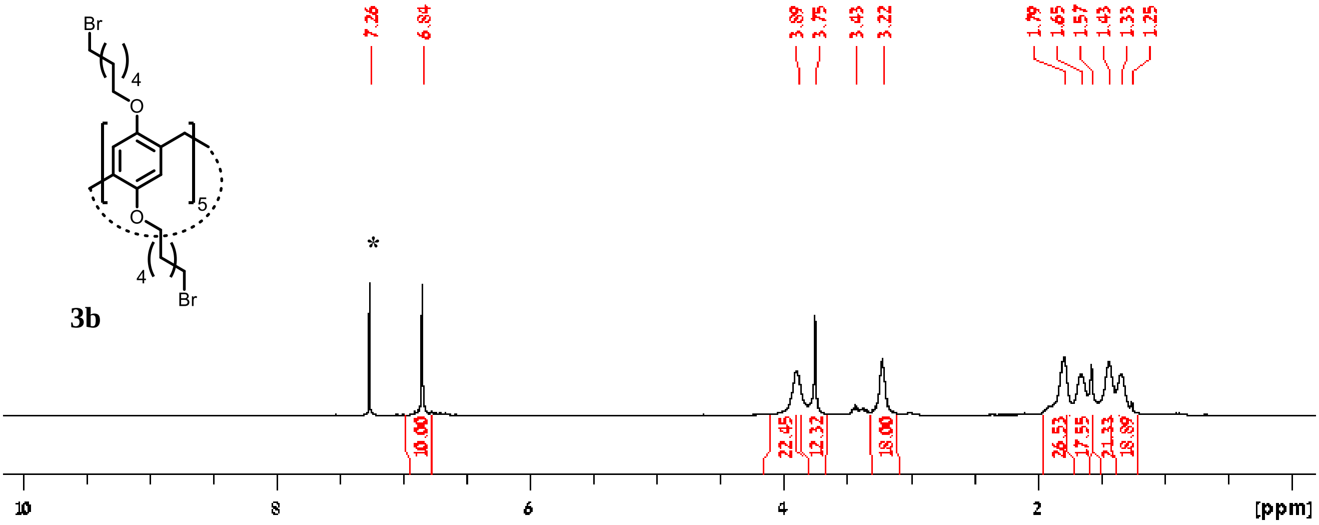

Figure S12. ^{1}H-NMR spectrum of **3b** in $CDCl_3$ (400 MHz). (*) Represents solvent peaks.

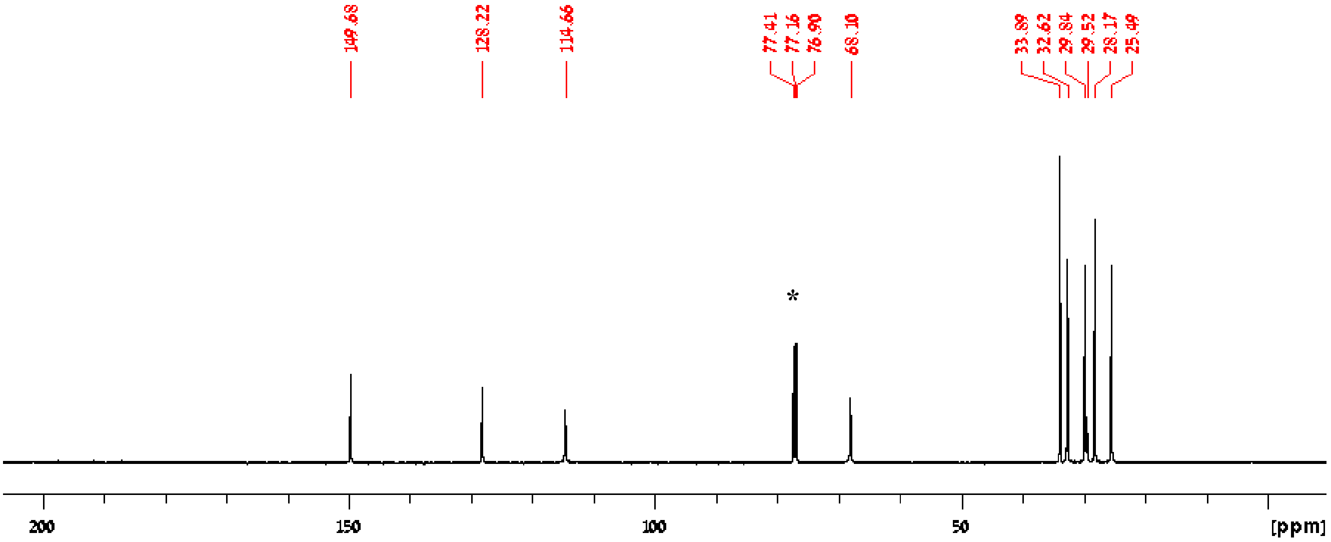

Figure S13. ^{13}C-NMR spectrum of **3b** in $CDCl_3$ (125 MHz). (*) Represents solvent peaks.

Synthesis of 3c[4]. To a solution of **2a** (5.5 g, 15.6 mmol) and paraformaldehyde (0.94 g, 31.2 mmol) in chloroform (275 mL) was added $BF_3{\cdot}OEt_2$ (4.43 g, 31.2 mmol). The reaction mixture was kept at 25 °C under argon atmosphere for 3 h. The reaction mixture was washed with water (2 × 100 mL) and brine (2 × 100 mL) and dried with sodium sulfate. The product was purified by column chromatography (silica gel; petroleum ether:dichloromethane) to afford **3c** as white solid (0.28 g, 1%).^{1}H-NMR (500 MHz, $CDCl_3$): δ 6.67 (s, Ar*H*, 12H), 3.92 (t, *J* = 5.0 Hz, $ArOCH_2(CH_2)_2Br$, 24H), 3.80 (s, $ArCH_2Ar$, 12H), 3.50 (t, *J* = 5.0 Hz, $ArO(CH_2)_2CH_2Br$, 24H), 2.22 (quint, *J* = 5.0 Hz, $ArOCH_2CH_2CH_2Br$, 24H) ppm. ^{13}C-NMR (100 MHz, $CDCl_3$): δ 150.4, 128.1, 115.1, 66.4, 33.8, 30.9, 30.4 ppm.

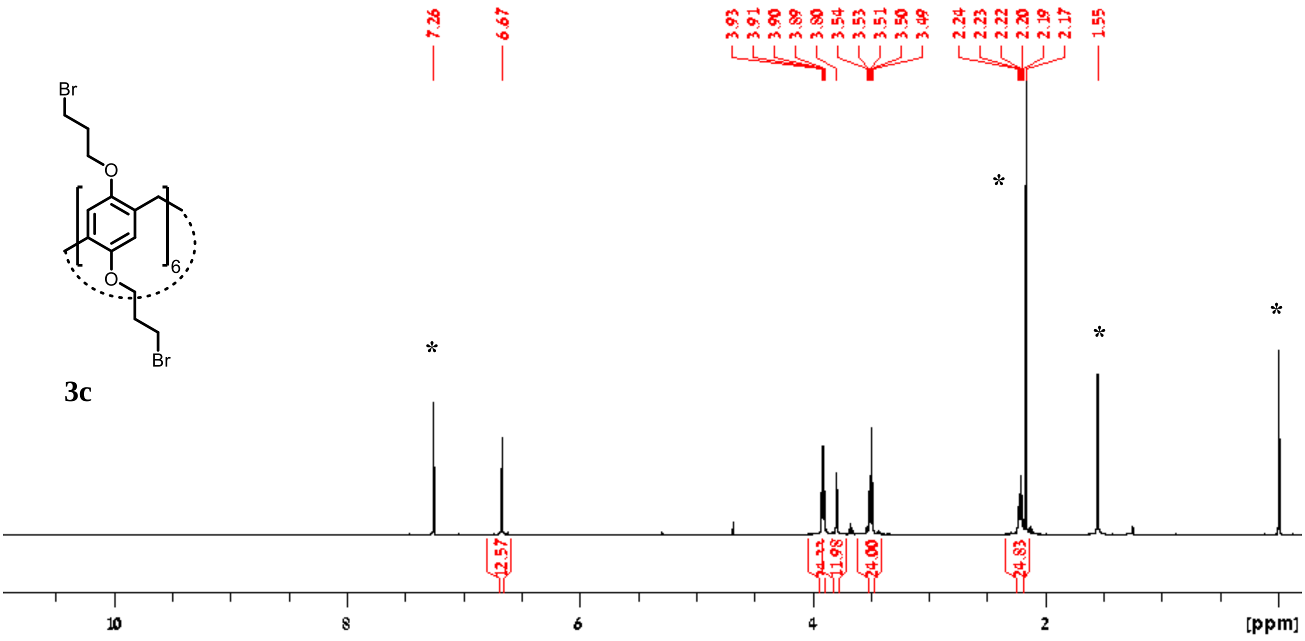

Figure S14. ^{1}H-NMR spectrum of **3c** in $CDCl_3$ (500 MHz). (*) Represents solvent peaks.

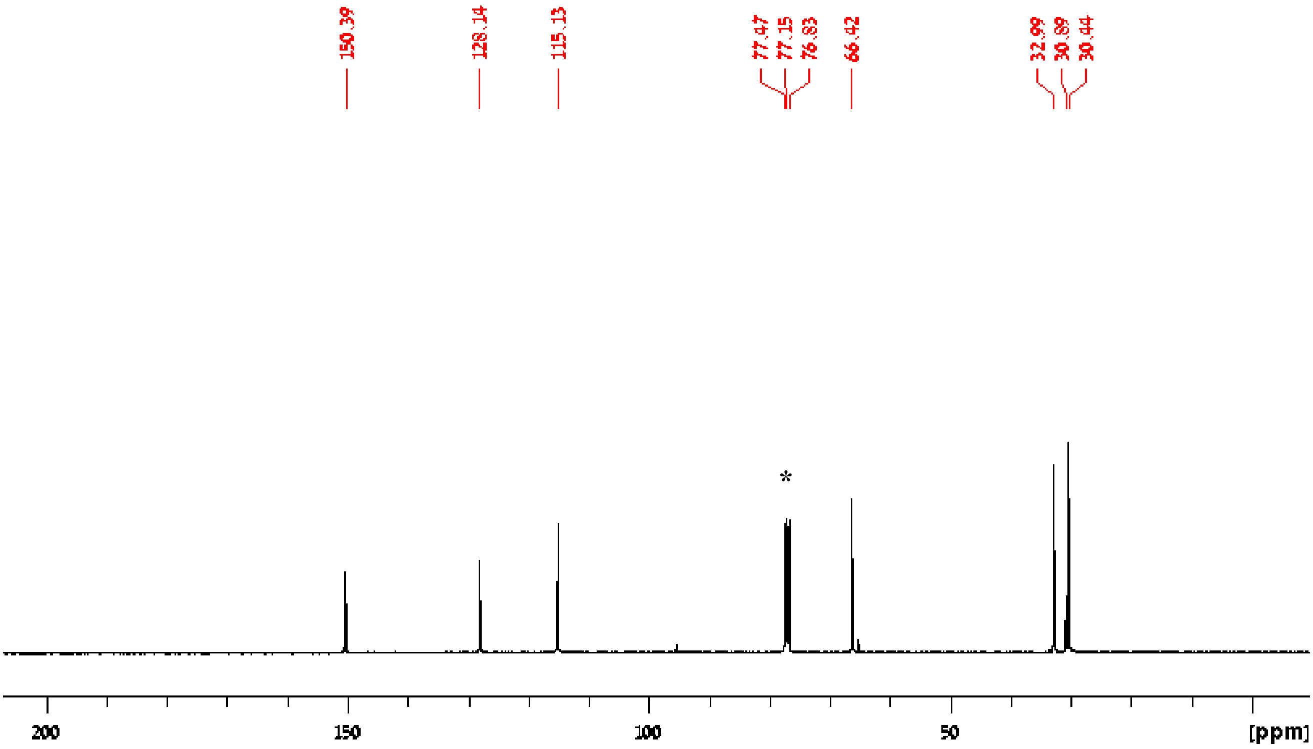

Figure S15. ^{13}C-NMR spectrum of **3c** in $CDCl_3$ (100 MHz). (*) Represents solvent peaks.

Synthesis of 2. N,N-dimethylbutylamine (1.35 mL, 9.6 mmol) was added to a solution of 1b (0.20 g, 0.12 mmol) in ethanol (3.5 mL). The resulting mixture was heated to 85oC in a pressure tube for 10 days. After cooling to 25°C, the product was precipitated by the addition of diethyl ether. The precipitate was filtered, and the solid was washed with diethyl ether and acetone. The solid was dissolved in water and concentrated to afford a white solid. Finally, the solid was dissolved in ethanol and crystallized by vapor diffusion of acetone, to afford a white solid (136.7 mg, 42.3%). 1H NMR (400 MHz, CD_3CN : D_2O (10:1)): δ 7.03 (s, Ar*H*, 10H), 4.63 & 4.37 (m, ArO$CH_2$$CH_2$N, 20H), 4.15 & 4.02 (m, ArO$CH_2$$CH_2$N, 20H), 3.82 (s, Ar$CH_2$Ar, 10H), 3.46 & 3.35 (m, N$CH_2$$CH_2$$CH_2$$CH_3$, 20H), 3.26 & 3.22 (s, NCH_3, 60H), 1.60 (br, N$CH_2$$CH_2$$CH_2$$CH_3$, 20H), 1.21 (q, J = 7.2 Hz, N$CH_2$$CH_2$$CH_2$$CH_3$, 20H), 0.86 (t, J = 7.3 Hz, N$CH_2$$CH_2$$CH_2$$CH_3$, 30H) ppm. ^{13}C NMR (100 MHz, CD_3CN + D_2O): δ 149.9, 129.9, 116.7, 66.0, 64.7, 63.7, 52.3, 52.2, 30.1, 25.2, 20.3, 14.0 ppm. MS: m/z Calcd. for $C_{115}H_{210}O_{10}N_{10}Br_{11}$ [M^+Br]- 2771.7126, found 2771.7188. Anal. calcd. for $C_{115}H_{210}Br_{10}N_{10}O_{10}\cdot$ 13.15H_2O: C, 47.16; H, 8.13; N, 4.78. Found: C, 46.89; H, 7.85; N, 4.72.

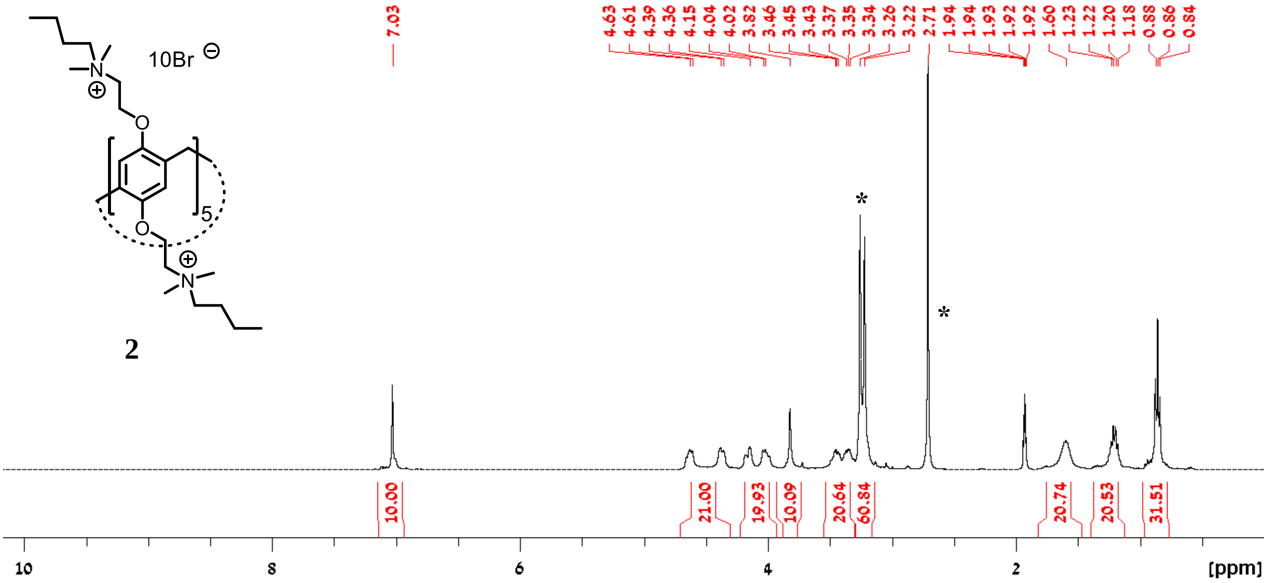

Figure S16. ^{1}H-NMR spectrum of **2** in CD_3CN:D_2O (10:1) solution (400 MHz). (*) Represents solvent peaks.

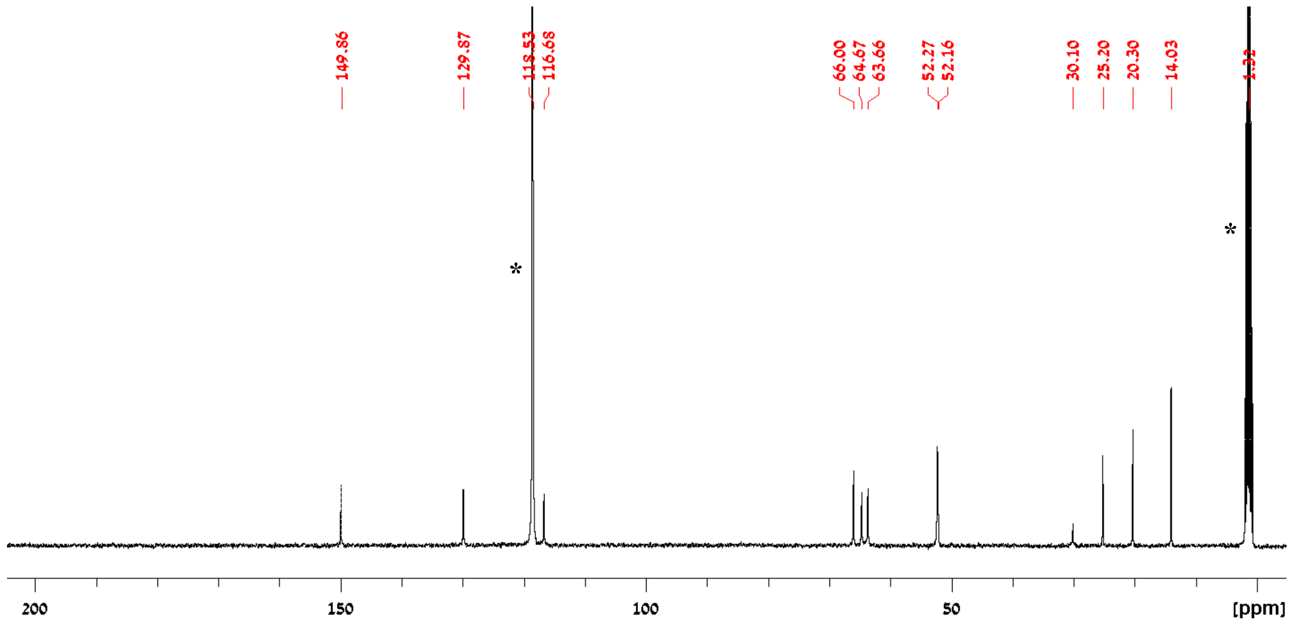

Figure S17. ^{13}C-NMR spectrum of **2** in $CD_3CN:D_2O$ (10:1) solution (100 MHz). (*) Represents solvent peaks.

Synthesis of 3. N-methyldibutylamine (1.37 mL, 7.14 mmol) was added to a solution of **1b** (0.15 g, 0.09 mmol) in ethanol (3.5 mL). The resulting mixture was heated to 90°C in a pressure tube for 4 days. After cooling to 25°C, the product was precipitated by the addition of diethyl ether. The precipitate was filtered, and the solid was washed with diethyl ether and acetone. Finally, the solid was dissolved in water and concentrated to afford a white solid. (81.5 mg, 29.3%). ^{1}H NMR (400 MHz, DMSO-d_6): δ 7.07 (s, Ar*H*, 10H), 4.72 - 4.10 (m, ArOCH_2CH$_2$N & ArOCH$_2$CH_2N, 40H), 3.76 (s, ArCH_2Ar, 10H), 3.52 (br, NCH_2CH$_2$CH$_2$CH$_3$, 40H), 3.25 (s, NCH_3, 30H), 1.69 (br, NCH$_2$CH_2CH$_2$CH$_3$, 40H), 1.29 (br, NCH$_2$CH$_2$CH_2CH$_3$, 40H), 0.90 (br, NCH$_2$CH$_2$CH$_2$CH_3, 60H). ^{13}C NMR (125 MHz, DMSO- d_6): δ 148.6, 128.0, 115.6, 62.1, 60.9, 60.9, 48.2, 28.6, 23.9, 23.6, 19.3, 13.6 ppm. MS: *m/z* Calcd. for $C_{145}H_{270}O_{10}N_{20}Br_{11}$ $[M+Br]^-$ 3185.1936, found 3185.1914. Anal. calcd. for $C_{145}H_{270}Br_{10}N_{10}O_{10}\cdot$ 5.25$H_2O\cdot$ 9.55HCl$\cdot$ 2.4HBr: C, 46.44; H, 7.86; N, 3.74. Found: C, 46.5; H, 7.81; N, 3.68.

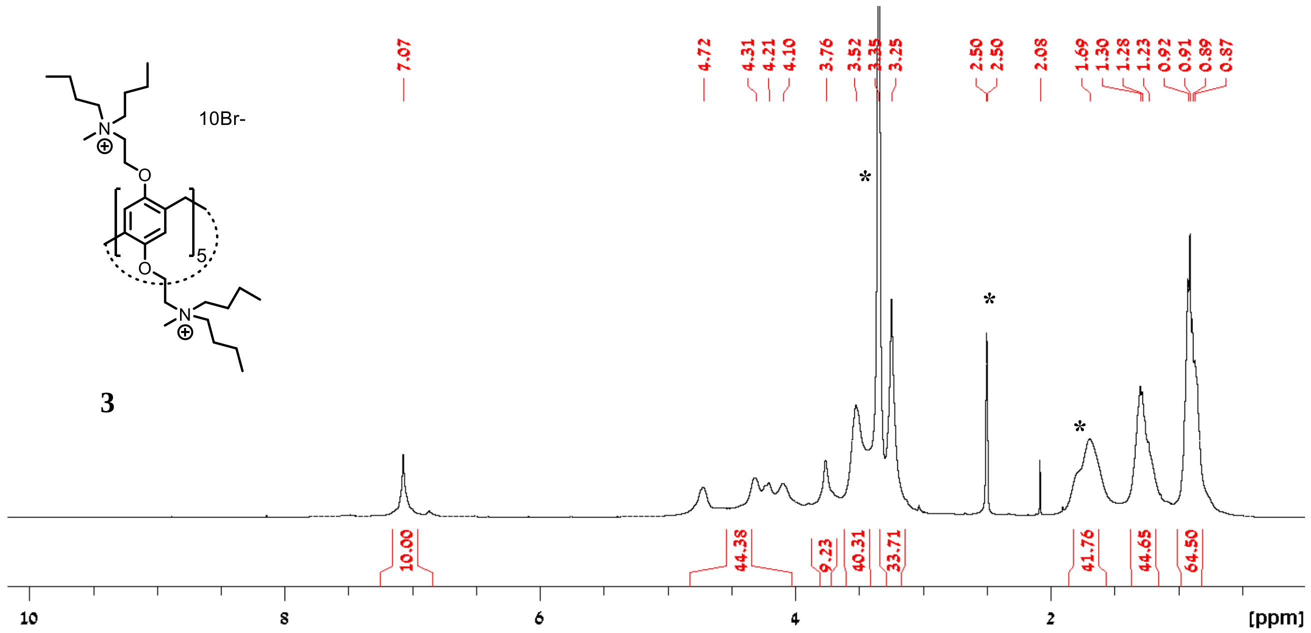

Figure S18. ^{1}H-NMR spectrum of **3** in DMSO-d_6 (400 MHz). (*) Represents solvent peaks.

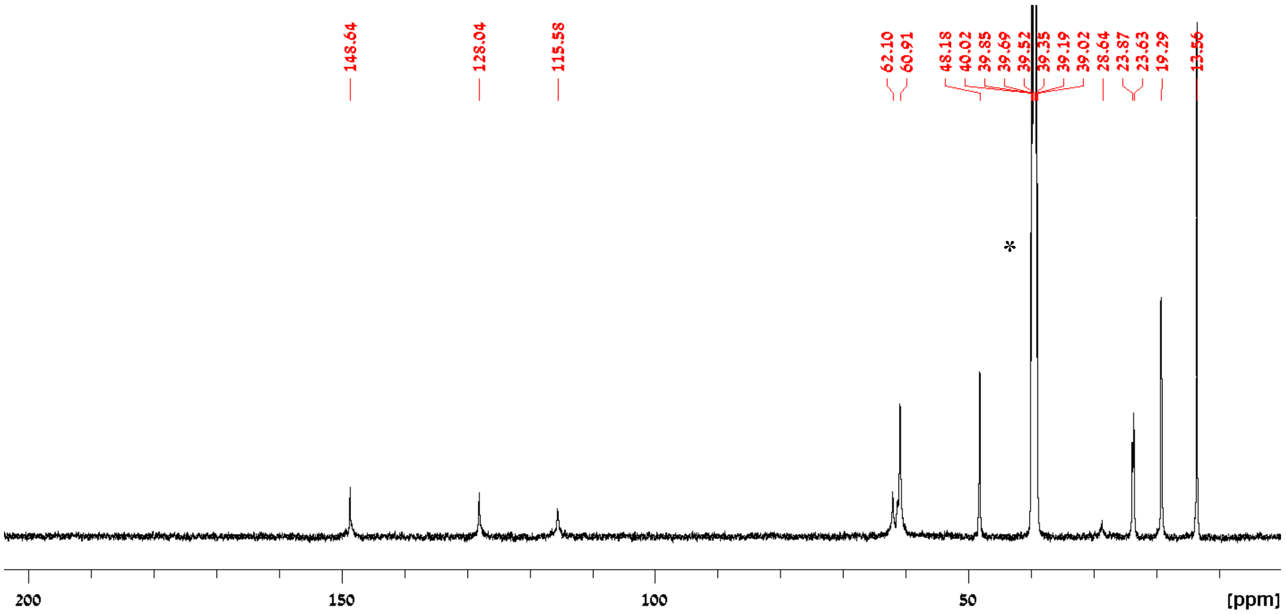

Figure S19. ^{13}C-NMR spectrum of **3** in DMSO-d_6 (100 MHz). (*) Represents solvent peaks.

Synthesis of 4. Tributylamine (2.5 mL, 17.3 mmol) was added to a solution of **2b** (0.20 g, 0.12 mmol) in acetonitrile (3 mL). The mixture was refluxed in a pressure tube for 7 days. After cooling to 25 °C, two layers obtained and separated. Diethyl-ether (10 mL) was added to acetonitrile solution and the precipitate formed was filtered and washed with diethyl ether and dried under vacuum to afford yellow solid (109 mg, 32%). ^{1}H-NMR (500 MHz, CD_3OD): δ 7.00 (s, Ar*H*, 10H), 4.25 (br, ArO*CH*$_2$CH$_2$, 20H), 3.88 (s, ArC*H*$_2$Ar, 10H), 3.60 (br, N*CH*$_2$CH$_2$, 20H), 3.36 (br, N*CH*$_2$(CH$_2$)$_2$CH$_3$,

60H), 2.29 (br, $NCH_2\mathit{CH_2}CH_2O$, 20H), 1.71 (br, $NCH_2\mathit{CH_2}CH_2CH_3$, 60H), 1.42 (br, $N(CH_2)_2\mathit{CH_2}CH_3$, 60H), 0.99 (br, $N(CH_2)_2CH_2\mathit{CH_3}$, 90H) ppm. . ^{13}C-NMR (125 MHz, CD_3OD-d_4): δ 150.6, 130.9, 116.5, 67.1, 59.9, 57.2, 30.6, 25.0, 23.76, 20.8, 14.1 ppm. Analytic HPLC chromatogram in Figure S74.

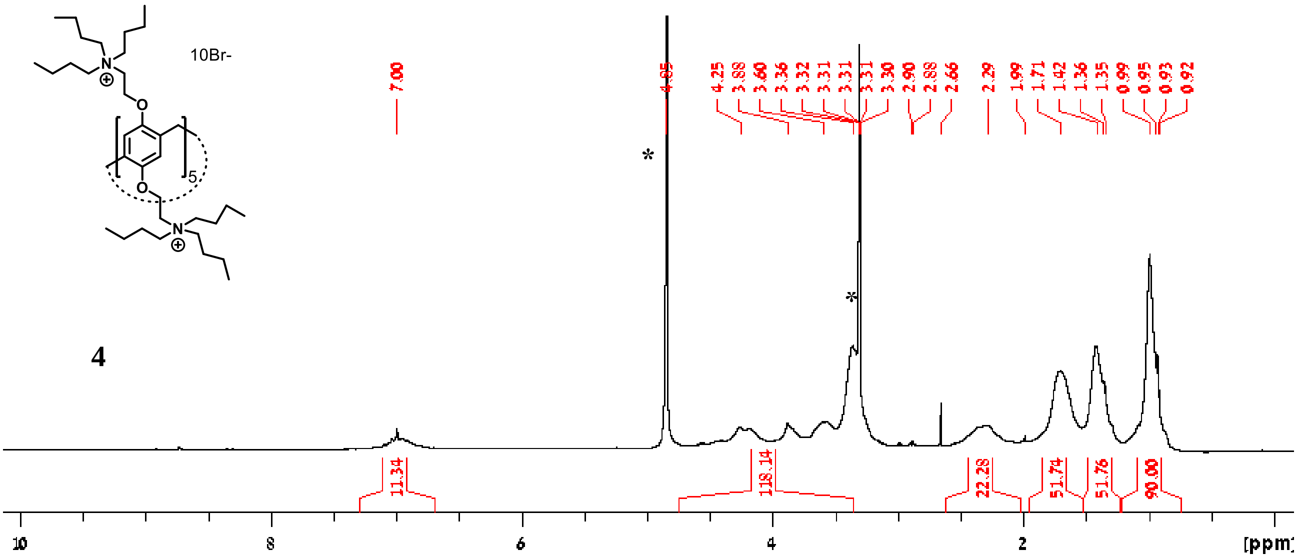

Figure S20. ^{1}H-NMR spectrum of **4** in methanol-d_4 (500 MHz). (*) Represents solvent peaks.

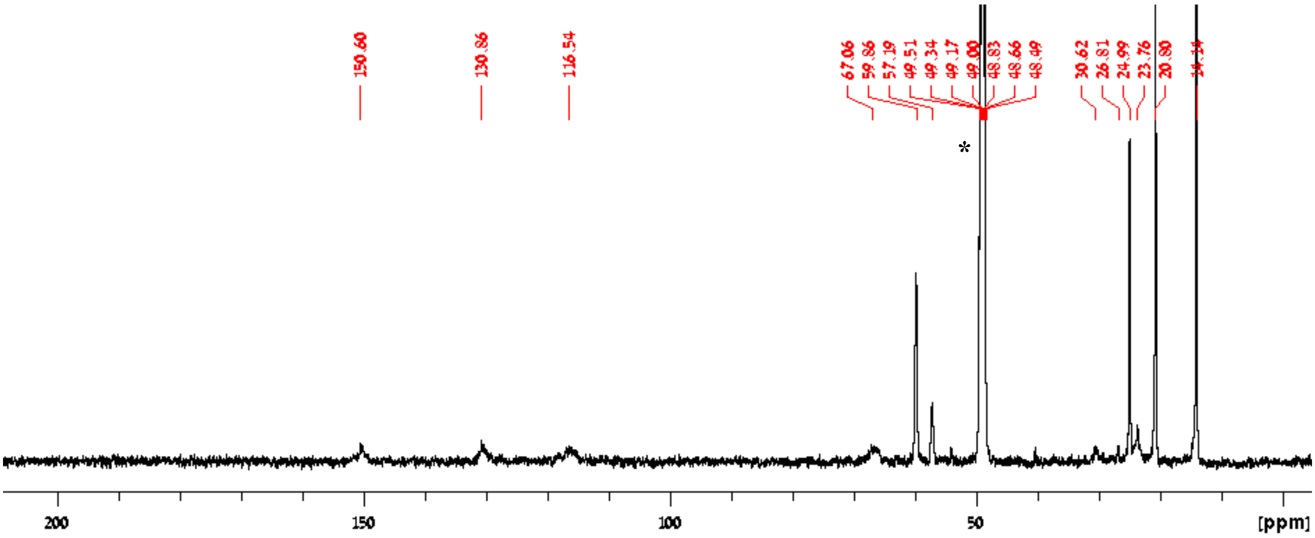

Figure S21. ^{13}C-NMR spectrum of **4** in methanol-d_4 (125 MHz). (*) Represents solvent peaks.

Synthesis of 5. Methyl iodide (0.5 mL, 7.96 mmol) was added to a solution of **1c** (500 mg, 0.27 mmol) in chloroform (5 mL) under vigorous stirring. The resulting mixture was heated to 60°C in a pressure tube for 3 days. After cooling to 25°C, and decanting the solvents, sticky orange oil was

obtained at the bottom of the flask. The oil was washed with chloroform for several times, then methanol (7 mL) was added to the flask and stirred for 30 min. The product was precipitated by the addition of diethyl ether (10 mL). The precipitate was filtered, washed with diethyl ether and dried under vacuum to afford **5** as a white powder (0.56 g, 62%). ^{1}H-NMR (500 MHz, DMSO-d_6): δ 6.89 (s, Ar*H*, 10H), 4.47 (m, ArO*CH*HCH$_2$, 10H), 4.35 (m, ArOCH*H*CH$_2$, 10H), 4.09 (m, NC*H*(CH$_3$)$_2$, 20H), 3.94 (m, ArOCH$_2$*CH$_2$*, 20H), 3.80 (s, ArC*H$_2$*Ar, 10H), 3.03 (s, NC*H$_3$*, 30H), 1.44 (m, NCH(*CH$_3$*)$_2$, 120H) ppm. ^{13}C-NMR (125 MHz, DMSO-d_6): δ 148.6, 128.4, 115.7, 64.1, 62.9, 55.8, 42.4, 28.8, 17.24 ppm. HRMS: m/z Calcd. for $C_{125}H_{230}N_{10}O_{10}$ I_{11} $[M+I]^-$ 3427.6985, found 3427.7289. Anal. calcd. for $C_{125}H_{230}N_{10}O_{10}I_{10}$ · 7.55 H_2O: C,43.67; H,7.19; N,4.07. Found: C,43.95; H,7.17; N,3.79.

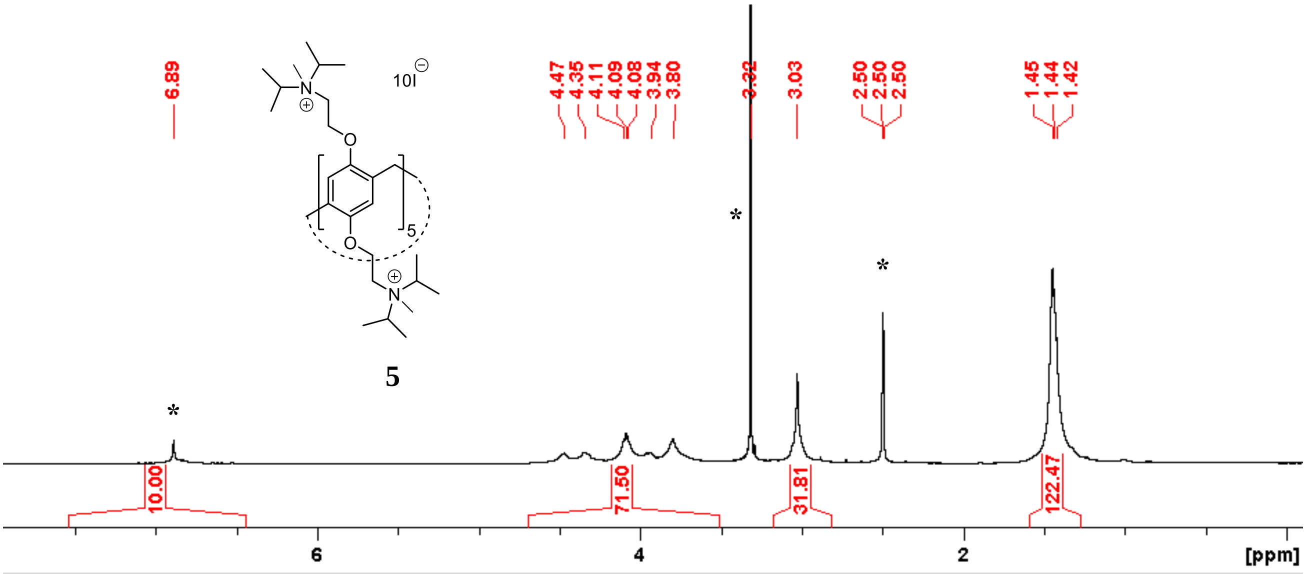

Figure S22. ^{1}H NMR spectrum of **5** in DMSO-d_6 (500 MHz). (*) Represents solvent peaks.

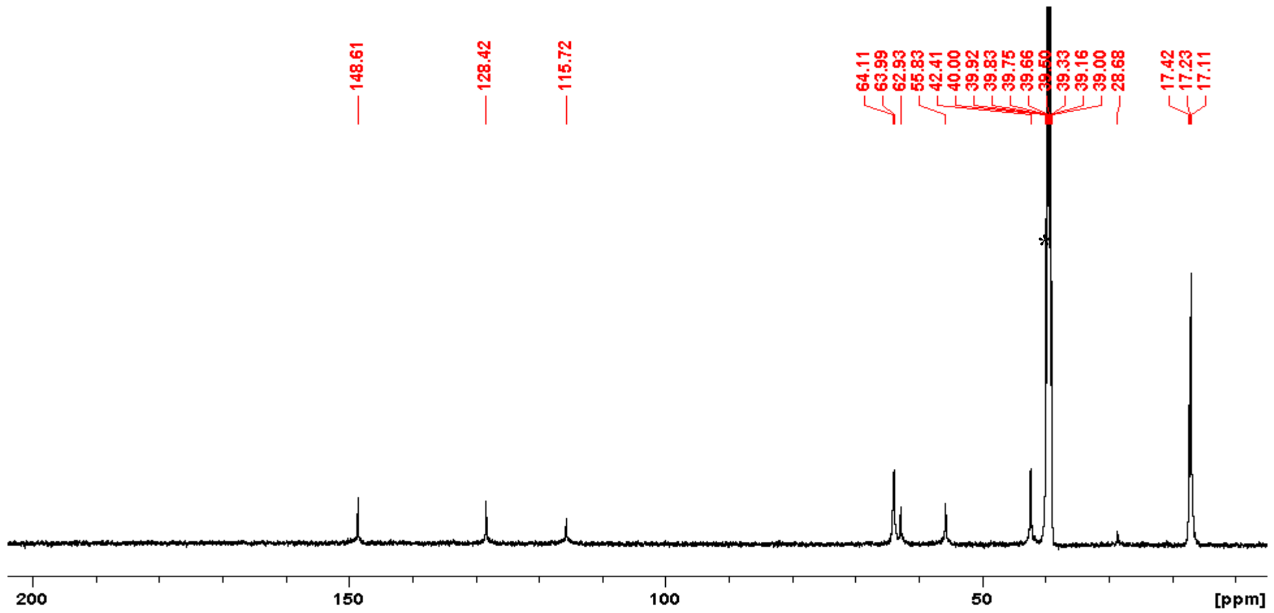

Figure S23. ^{13}C NMR spectrum of **5** in DMSO-d_6 (125 MHz). (*) Represents solvent peaks.

Synthesis of 6. N,N-dimethylhexylamine (1.24 mL, 7.12 mmol) was added to a solution of **1b** (0.15 g, 0.09 mmol) in ethanol (3 mL). The resulting mixture was heated to 85°C in a pressure tube for 4 days. After cooling to 25 °C, the product was precipitated by the addition of diethyl ether. The precipitate was filtered, and the solid was washed with diethyl ether and acetone. The solid was dissolved in water and concentrated to afford a white solid. Finally, the solid was dissolved in ethanol and crystallized by the addition of diethyl ether/acetone mixture, to afford a white solid (152 mg, 54.7%). ^{1}H NMR (400 MHz, D_2O): δ 7.07 (s, Ar*H*, 10H), 4.62 (br, ArOCH_2CH_2N, 20H), 3.95-3.90 (m, ArOCH_2CH_2N & ArCH_2Ar, 30H), 3.40-3.24 (m, NCH_3 & NC$H_2$$(CH_2)_4CH_3$, 80H), 1.56 (br, N$CH_2CH_2$$(CH_2)_3CH_3$, 20H), 1.28-1.07 (m, N$(CH_2)_2CH_2$$(CH_2)_2CH_3$, N$(CH_2)_3CH_2$$CH_2CH_3$ & N$(CH_2)_4$C$H_2$$CH_3$, 60H), 0.80 (t, J = 7.2 Hz, N$(CH_2)_5$CH_3, 30H) ppm. ^{13}C NMR (100 MHz, D_2O): δ 149.1, 129.6, 116.2, 65.4, 63.3, 62.3, 52.2, 51.8, 30.8, 29.7, 25.4, 22.4, 22.1, 13.6 ppm. MS: *m/z* Calcd. for $C_{135}H_{250}O_{10}N_{20}Br_{11}$ $[M+Br]^-$ 3052.0256, found 3052.0171. Anal. calcd. for $C_{135}H_{250}Br_{10}N_{10}O_{10}\cdot 12.8H_2O$: C, 50.62; H, 8.67; N, 4.37. Found: C, 50.48; H, 8.53; N, 4.39.

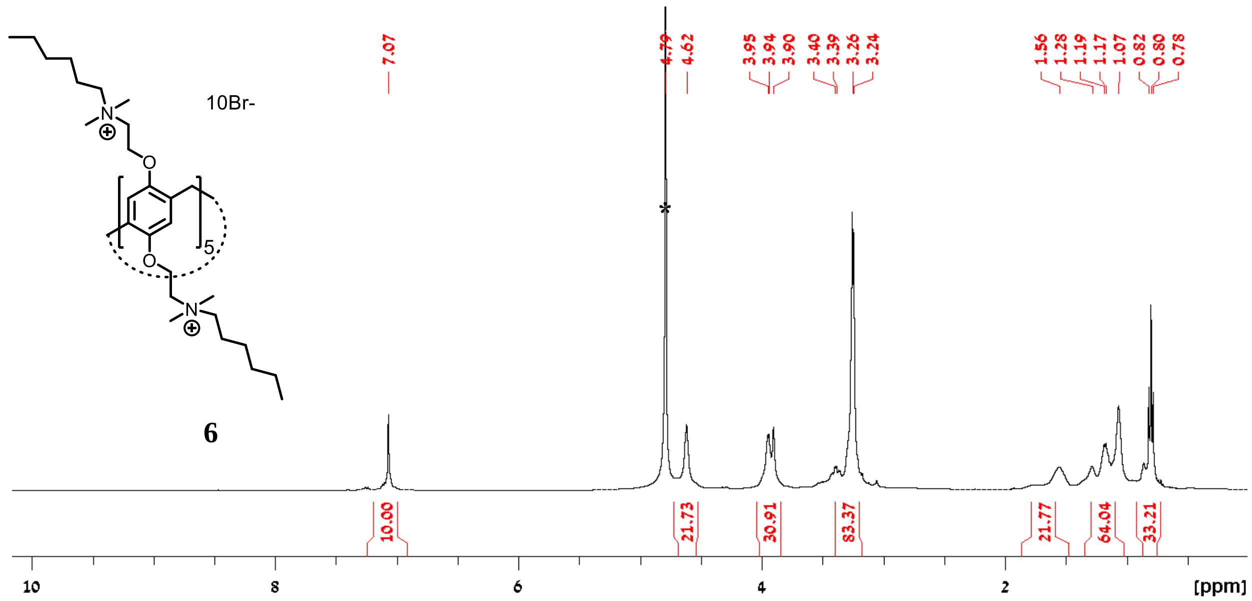

Figure S24. ^{1}H-NMR spectrum of **6** in D_2O (400 MHz). (*) Represents solvent peaks.

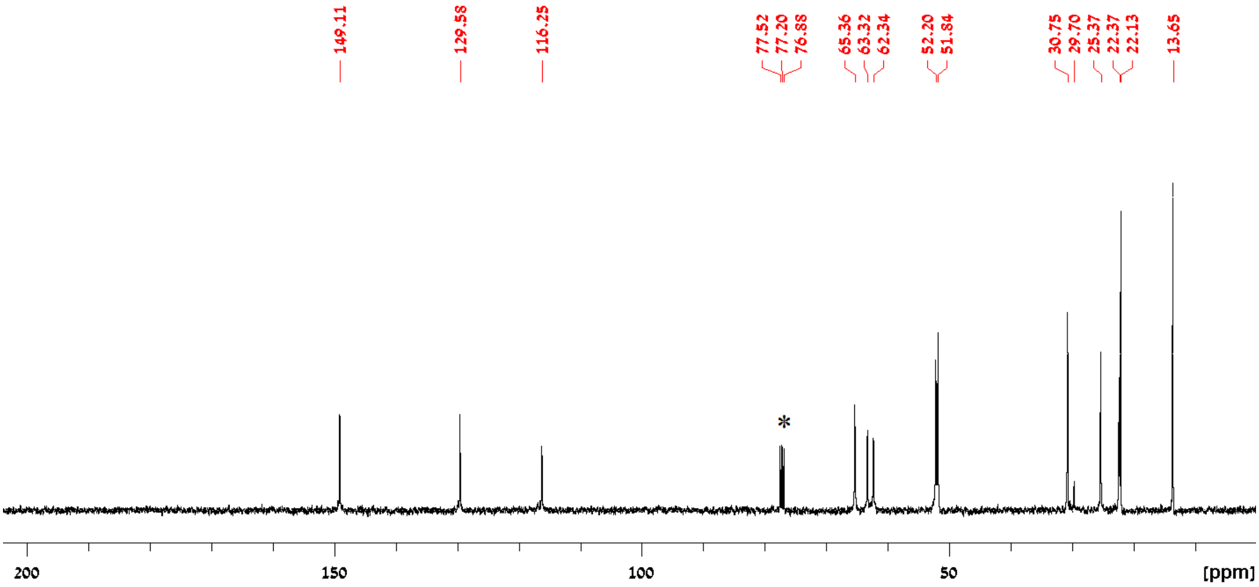

Figure S25. ^{13}C-NMR spectrum of **6** in D_2O and $CDCl_3$ as external reference (100 MHz). (*) Represents solvent peaks.

Synthesis of 7. 1-Iodododecane (0.75 mL, 3.04 mmol) was added to a solution of **1d** (0.1 g, 0.076 mmol) in ethanol (4 mL). The reaction mixture was heated to 85°C for 5 days in a pressure tube. After cooling to 25°C the solvent was removed. The obtained solid was dissolved in EtOH and precipitated by the addition of Et_2O while stirring. The solid was filtered and recrystallized from isopropanol to afford an orange solid (0.075g, 24%). ^{1}H-NMR (400 MHz, DMSO-d_6): δ 6.97 (s,

Ar*H*, 10H), 4.63 & 4.32 (m, ArO$\mathit{CH_2}$$CH_2$N, 20H), 4.08 & 3.98 (m, ArO$CH_2$$\mathit{CH_2}$N, 20H), 3.76 (brs, Ar$\mathit{CH_2}$Ar, 10H), 3.55 (m, N$\mathit{CH_2}$$(CH_2)_{10}$$CH_3$, 20H), 3.28 & 3.26 (s, N$(\mathit{CH_3})_2$, 60H), 1.79 (m, N$CH_2$$\mathit{CH_2}$$(CH_2)_9$$CH_3$, 20H), 1.24 (m, N$(CH_2)_2$$(\mathit{CH_2})_9$$CH_3$, 180H), 0.86 (t, J = 6.9 Hz, N$(CH_2)_{11}$$\mathit{CH_3}$, 30H) ppm. ^{13}C-NMR (100 MHz, DMSO-d_6): δ 148.6, 127.6, 115.2, 64.2, 63.4, 61.8, 50.8, 50.4, 30.8, 31.3, 31.3, 31.4, 29.1, 29.1, 29.1, 28.8, 28.8, 25.9, 22.1, 22.1, 14.0 ppm. HRMS: m/z Calcd. for $C_{195}H_{370}N_{10}O_{10}I_{11}$ $[M+I]^-$ 4408.8472, found 4408.8244. Anal. calcd. for $C_{195}H_{370}N_{10}O_{10}I_{10}$ ·1.5 H_2O ·9.75HCl ·3.1 HBr : C,47.63; H,7.91; N,2.85. Found: C,47.4; H,7.68; N,3.08.

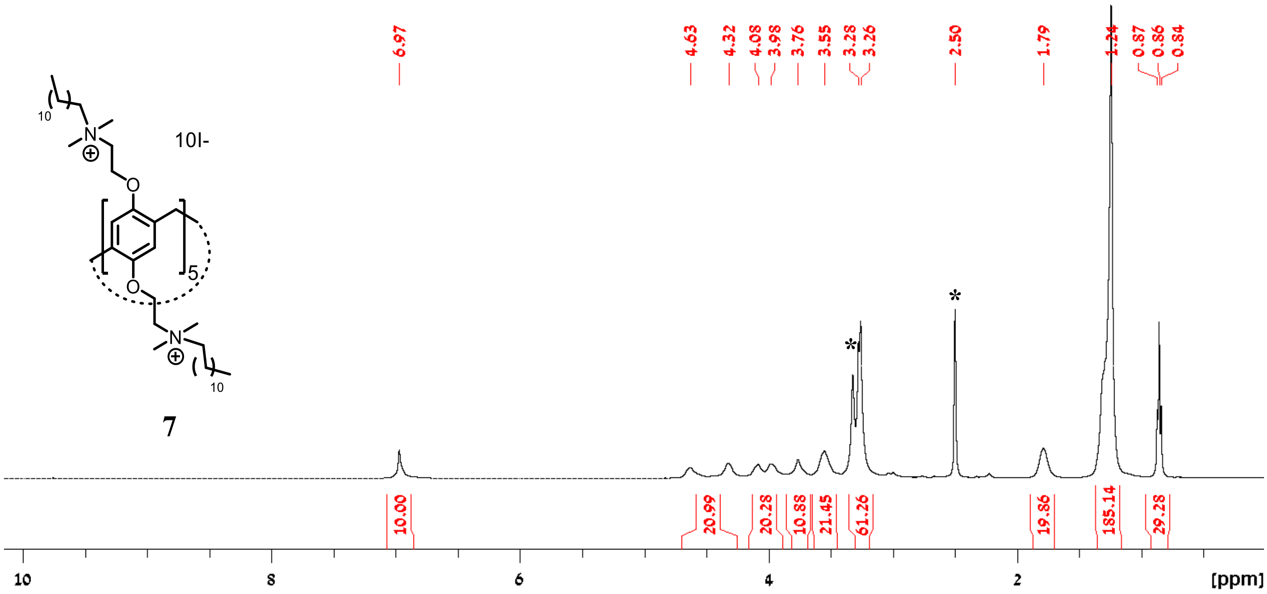

Figure S26. ^{1}H-NMR spectrum of **7** in DMSO-d_6 (400 MHz). (*) Represents solvent peaks.

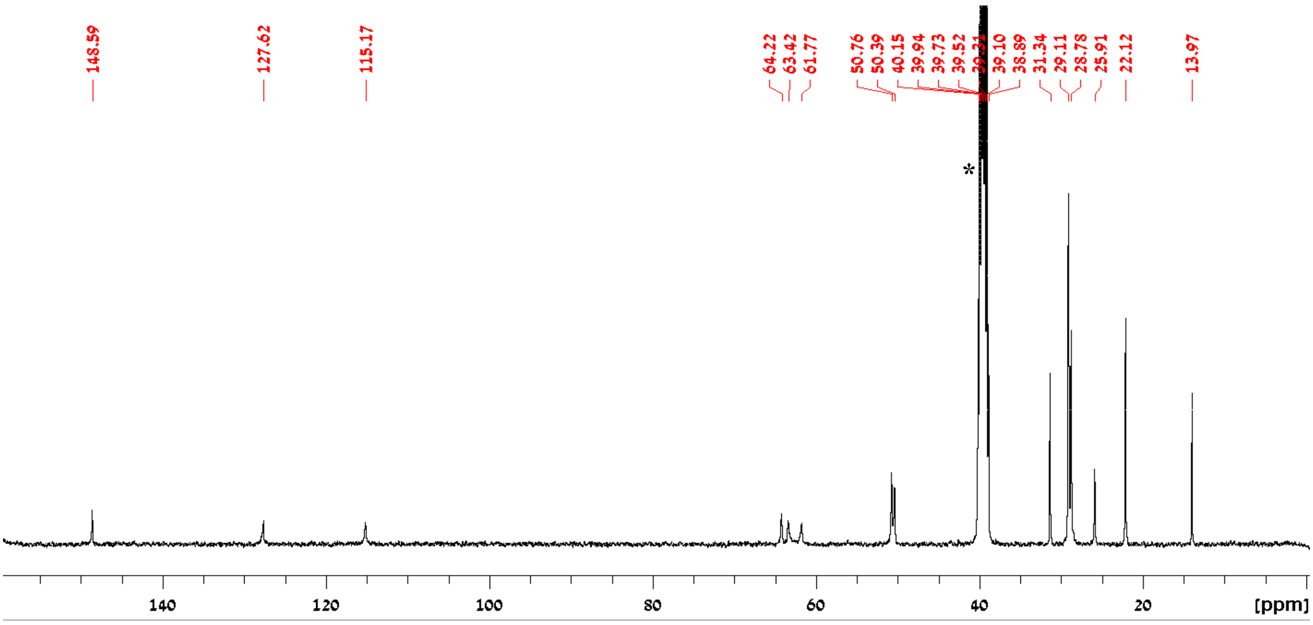

Figure S27. ^{13}C-NMR spectrum of **7** in DMSO-d_6 (100 MHz). (*) Represents solvent peaks.

Synthesis of 8. Trimethylamine (33% in ethanol, 0.85 mL, 3.56 mmol) was added to a solution of **3b** (0.20 g, 0.09 mmol) in ethanol (5.0 mL). The resulting mixture was refluxed in a pressure tube for 24 h. After cooling to 25 °C, diethyl-ether (10 mL) was added and the precipitate formed was filtered and washed with diethyl ether and dried under vacuum to afford **8** as white solid (0.23 g, 91%). ^{1}H NMR (400 MHz, DMSO-d$_6$): δ 6.82 (s, Ar*H*, 10H), 4.02 (s, ArC*H*$_2$Ar, 10H), 3.71 (br, ArO*CH*$_2$CH$_2$(CH$_2$)$_4$, 20H), 3.48 (br, N*CH*$_2$CH$_2$(CH$_2$)$_4$, 20H), 3.16 (s, CH$_2$N*CH*$_3$, 90H),1.75 (br, ArOCH$_2$*CH*$_2$(CH$_2$)$_4$, 20H), 1.75 (br, ArO(CH$_2$)$_4$*CH*$_2$CH$_2$, 20H), 1.59 (br, ArO(CH$_2$)$_3$*CH*$_2$(CH$_2$)$_2$, 20H), 1.37 (br, ArO(CH$_2$)$_2$*CH*$_2$(CH$_2$)$_3$, 20H) ppm. ^{13}C NMR (100 MHz, DMSO-d$_6$): δ 149.0, 128.0, 114.1, 67.8, 65.0, 52.1, 29.1, 28.8, 25.8, 25.4, 22.1 ppm. HRMS: m/z Calcd. for $C_{125}H_{230}O_{10}N_{10}Br_{11}$ [M+Br]- 2911.8621, found 2911.8691. Analytic HPLC chromatogram in Figure S74.

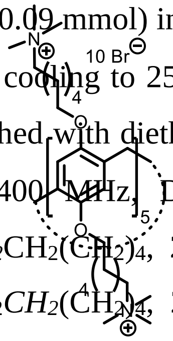

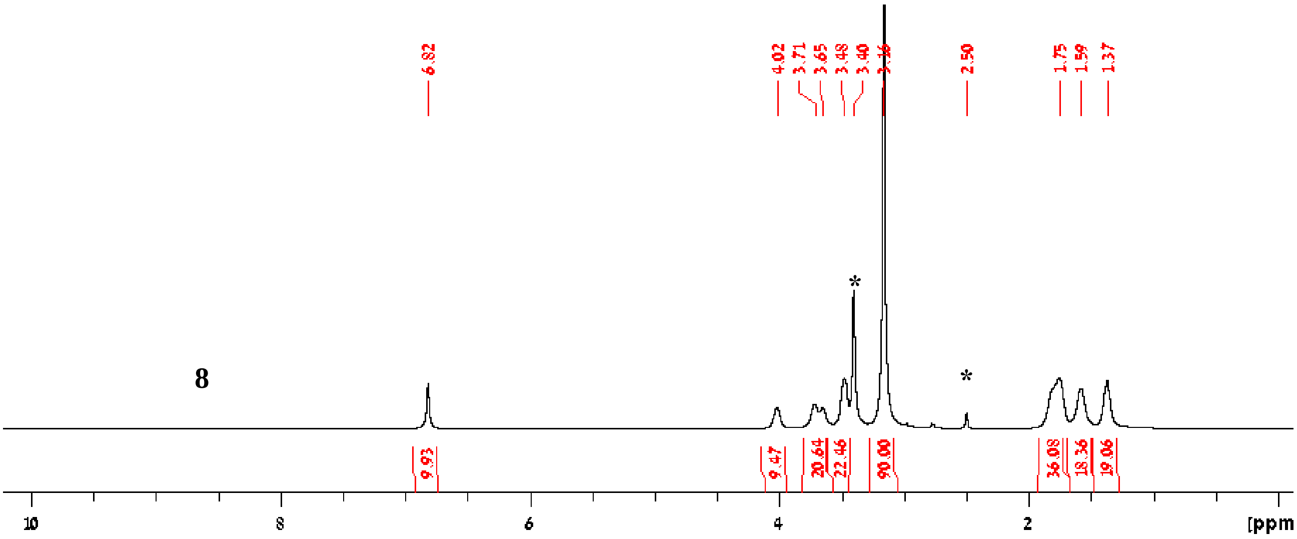

Figure S28. ^{1}H-NMR spectrum of **8** in DMSO-*d*$_6$ (400 MHz). (*) Represents solvent peaks.

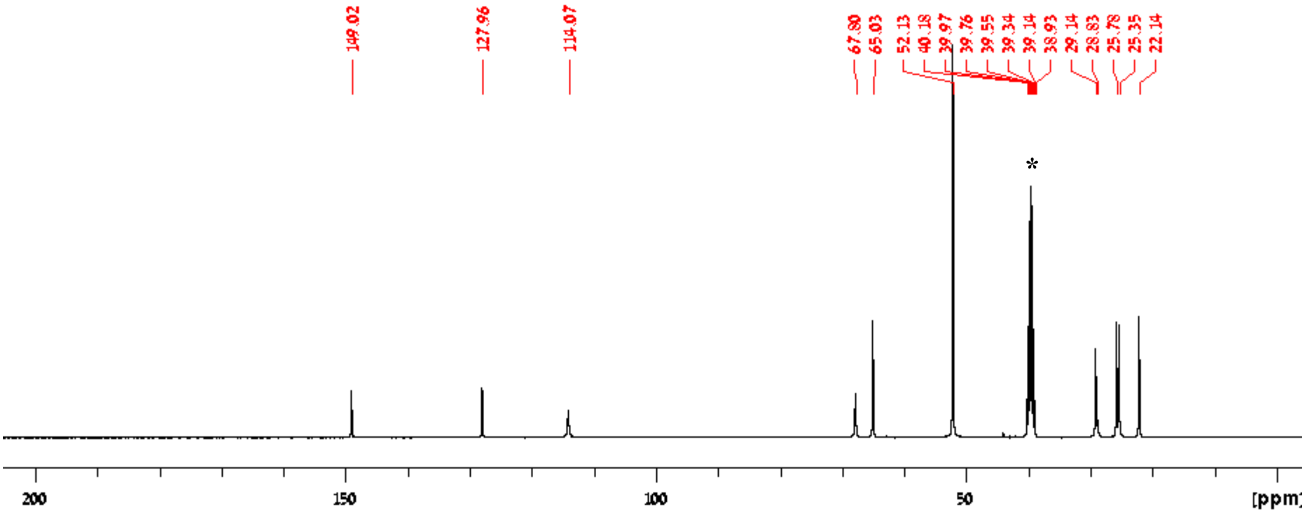

Figure S29. ^{13}C-NMR spectrum of **8** in DMSO-*d*$_6$ (100 MHz). (*) Represents solvent peaks.

Synthesis of 9. Trimethylamine (33% in ethanol, 0.43mL, 1.82 mmol) was added to a solution of **3c** (0.10 g, 0.046 mmol) in ethanol (5.0 mL). The mixture was refluxed in a pressure tube for 48 h. After cooling to 25 °C, stirring continued for 48 hr. more. The precipitate formed was filtrated and washed with water, then concentrated to obtain yellow powder (0.13 g, 96%). ^{1}H-NMR (500 MHz, D_2O): δ 6.90 (s, Ar*H*, 12H), 4.07 (t, *J* = 5.0 Hz, ArOC*H*$_2$(CH_2)$_2$Br, 24H), 3.93 (s, ArC*H*$_2$Ar, 12H), 3.37 (t, *J* = 10.0 Hz, ArO(CH_2)$_2$C*H*$_2$N, 24H), 3.07 (s, NC*H*$_3$, 108H),2.20 (br, ArOCH$_2$*CH*$_2$CH$_2$Br, 24H) ppm. ^{13}C-NMR (125 MHz, D_2O): 150.5, 129.5, 116.9, 66.6, 64.3, 53.4, 31.1, 23.4 ppm. Anal. calcd. for $C_{114}H_{204}Br_{12}N_{12}O_{12}$. 31.3 H_2O. 2.75 C_6H_2O: C,41.92; H,7.01; N,4.92. Found: C,42.30; H,7.40; N,4.54.

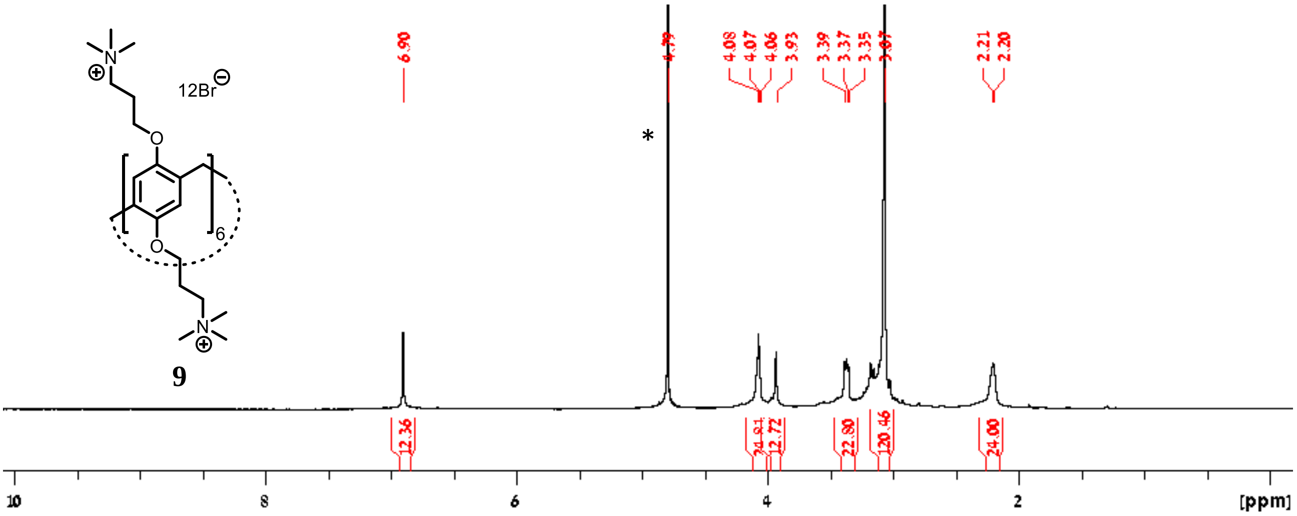

Figure S30. ^{1}H-NMR spectrum of **9** in D_2O (500 MHz). (*) Represents solvent peaks.

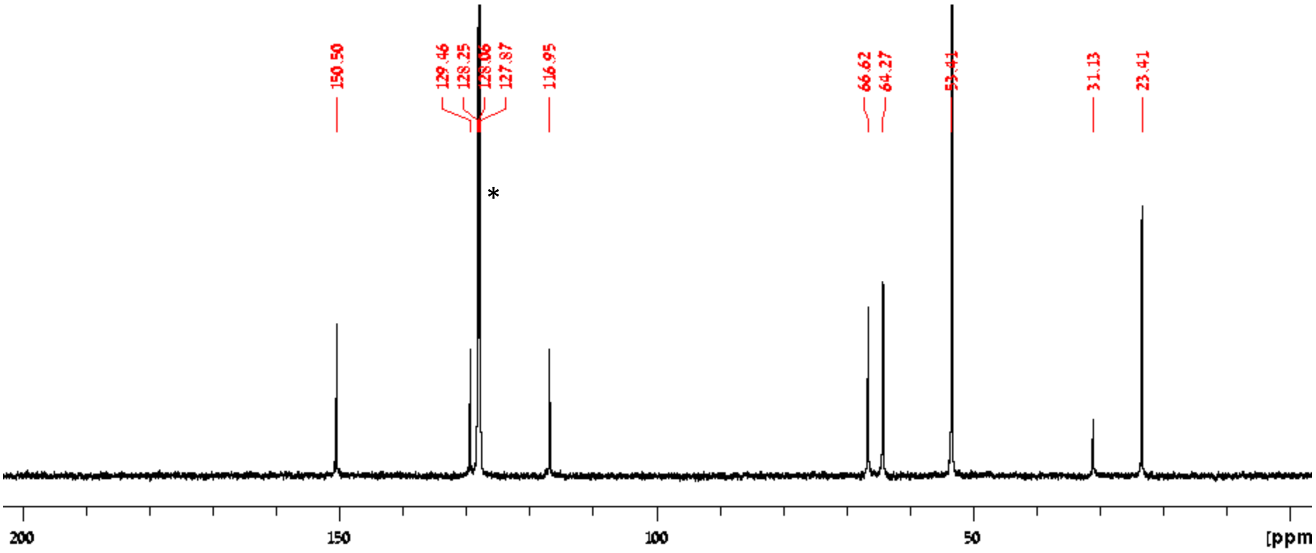

Figure S31. ^{13}C NMR spectrum of **9** in D_2O and C_6D_6 as external reference (125 MHz). (*) Represents solvent peaks.

Synthesis of 10[5]. A mixture of **3c** (0.1 g, 0.05 mmol) and *N*-methylimidazole (0.2 mL, 2.5 mmol) in toluene (4.0 mL) was kept at 120 °C for 7 days. After cooling to 25 °C, the product was precipitated by the addition of diethyl ether. The precipitate was filtered, and the solid was washed with diethyl ether, and the residue was dissolved in ethanol and concentrated by vacuum to afford a solid (0.13 g, 87%). ^{1}H-NMR (500 MHz, D_2O): δ 7.42 (s, $CH_3NC\textit{H}CHN$, 12H) , 7.38 (s, $CH_3NCHC\textit{H}N$, 12H), 6.75 (s, Ar*H*, 12H), 4.26 (br, $ArOC\textit{H}_2(CH_2)_2N$, 24H), 3.90 (s, $ArC\textit{H}_2Ar$, 12H), 3.81 (br, $ArO(CH_2)_2C\textit{H}_2N$, 24H),3.78 (s, $\textit{CH}_3NCHCHN$, 36H), 2.30 (br, $ArOCH_2\textit{CH}_2CH_2Br$, 24H) ppm. ^{13}C-NMR (125 MHz, $CDCl_3$): δ 150.3, 136.3, 129.0, 124.1, 122.8, 115.9, 65.9, 47.0, 36.2, 30.7, 29.8 ppm. Anal. calcd. for $C_{126}H_{168}Br_{12}N_{24}O_{12}$. 11.2 H_2O. 1.1 C_6H_2O: C,47.75; H,5.34; N,10.61. Found: C,45.37; H,5.40; N,9.42.

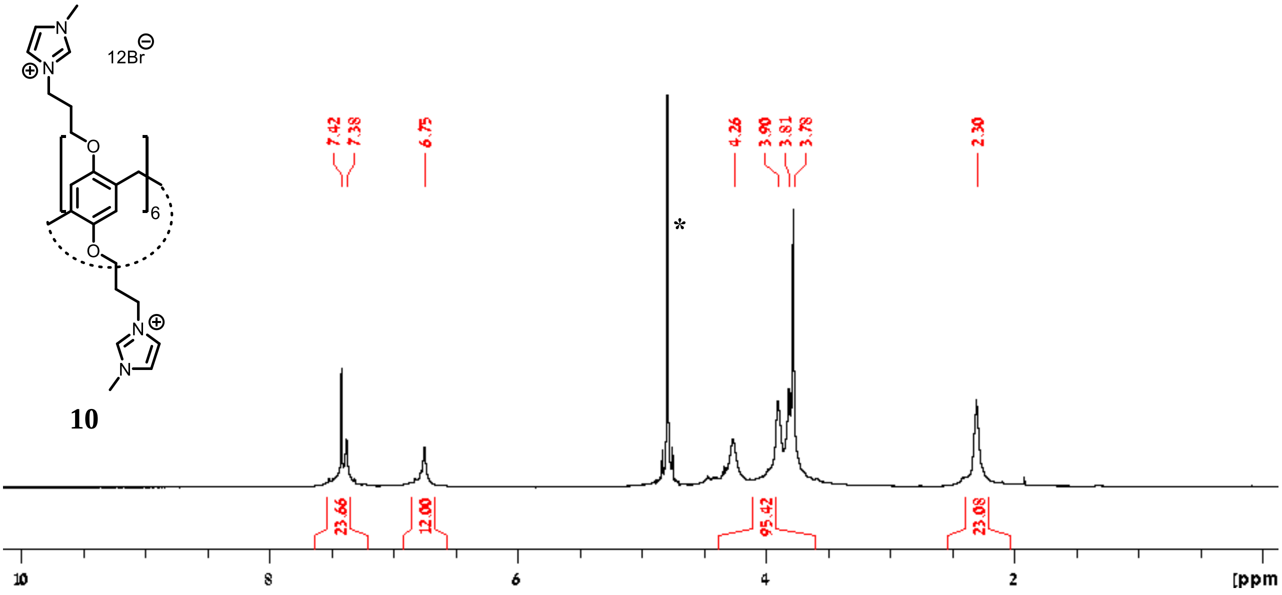

Figure S32. ^{1}H-NMR spectrum of **10** in D_2O (500 MHz). (*) Represents solvent peaks.

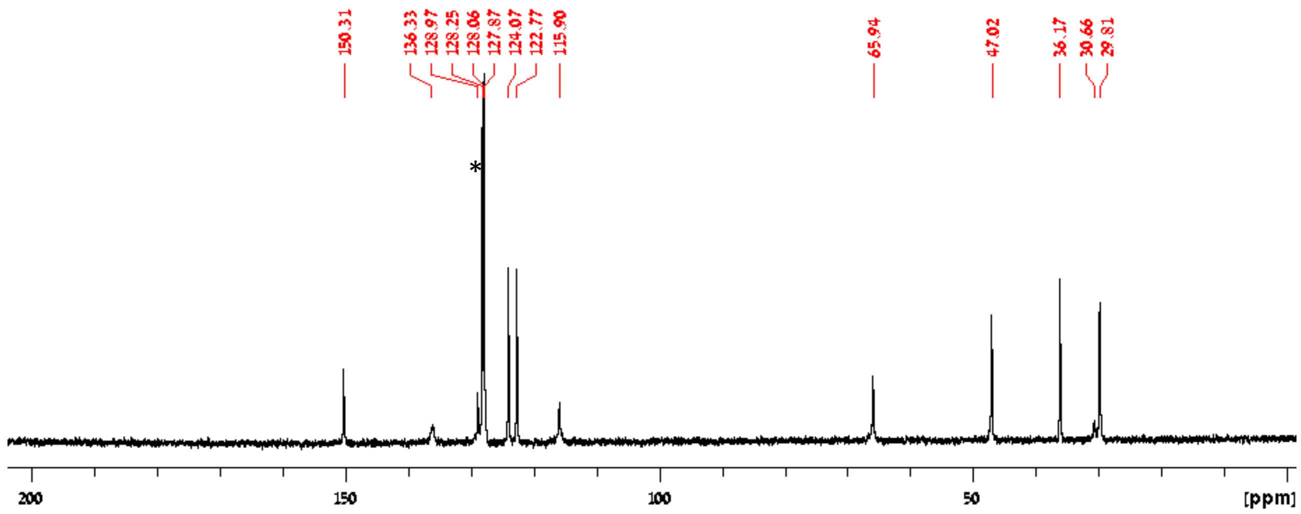

Figure S33. ^{13}C-NMR spectrum of **10** in D_2O and C_6D_6 as external reference (125 MHz). (*) Represents solvent peaks.

Synthesis of 11. 1b (0.2 g, 0.12 mmol) was added to dry pyridine (5 ml) and the solution was heated to 115°C for 3 days in a pressure tube. After cooling to 25 °C, a precipitate was observed, and after removing the solvent the remaining solid was washed with diethyl ether. The solid was dissolved in water and lyophilized to afford a light pink solid (134 mg, 43.8%). ^{1}H NMR (400 MHz, D_2O): δ 8.73 (d, J = 5.8 Hz, pyridine-H, 20H), 8.31 (t, J = 7.8 Hz, pyridine-H, 10H), 7.90 (t, J = 7.2 Hz, pyridine-H, 20H), 6.54 (s, ArH, 10H), 4.93 (t, J = 4.7 Hz, ArOCH_2CH$_2$N, 20H), 4.47 (t, J = 4.7 Hz, ArOCH$_2$CH_2N, 20H), 3.41 (s, ArCH_2Ar, 10H). ^{13}C NMR (100 MHz, D_2O): δ 149.5, 146.5, 145.0, 129.3, 128.5, 116.1, 67.5, 61.2, 29.2 ppm. HRMS: m/z Calcd. for $C_{105}H_{110}O_{10}N_{10}Br_{11}$ $[M+Br]^-$ 2544.9362, found 2544.9392. Analytic HPLC chromatogram in Figure S74.

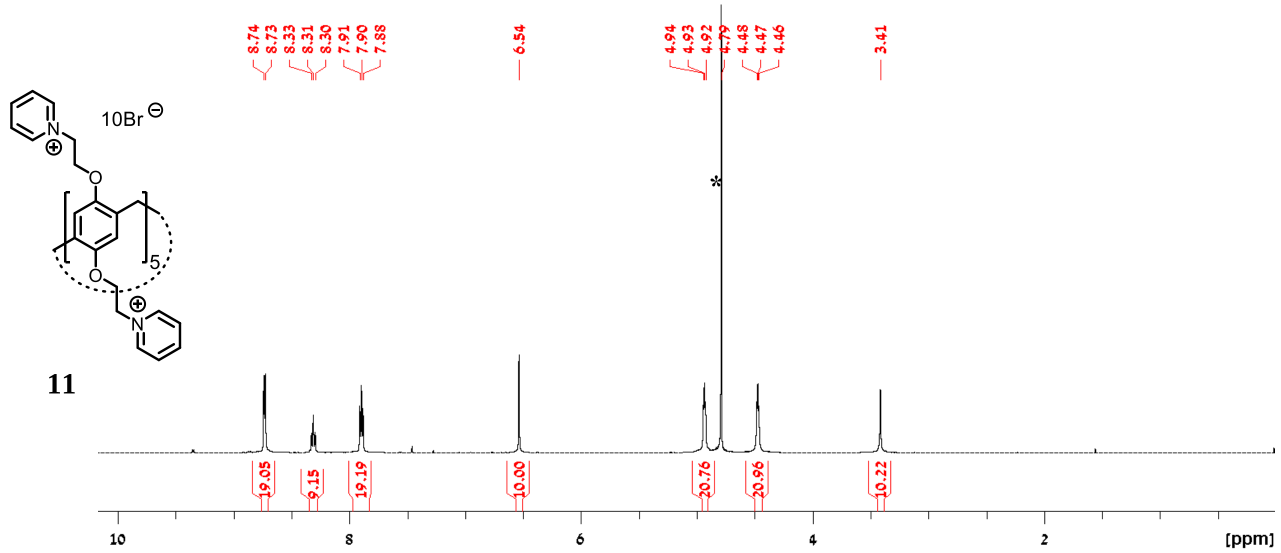

11

Figure S34. ^{1}H-NMR spectrum of **11** in D_2O (400 MHz). (*) Represents solvent peaks.

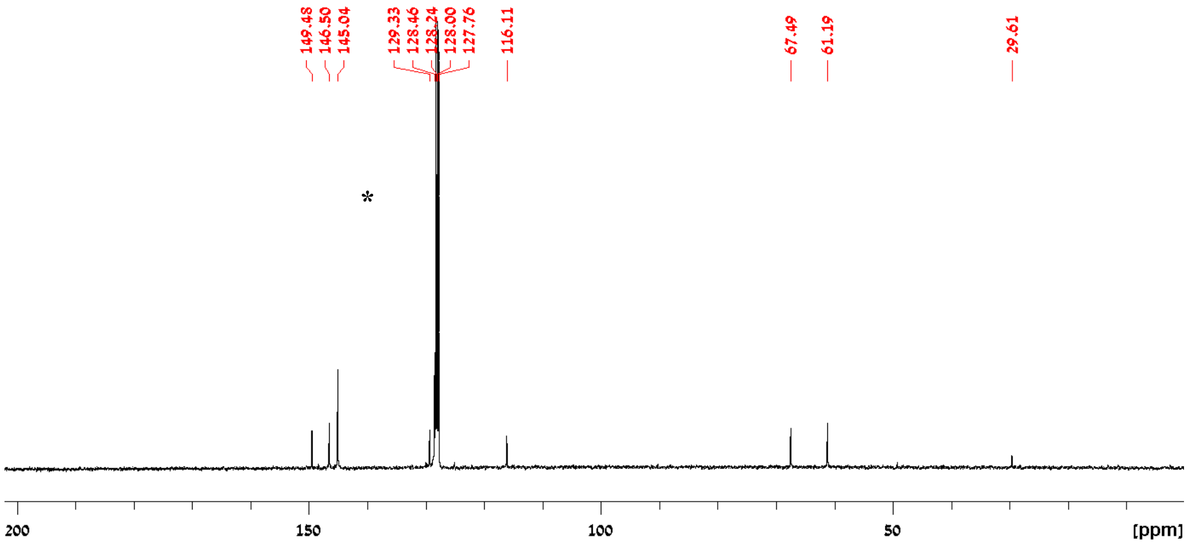

Figure S35. ^{13}C-NMR spectrum of **11** in D_2O and C_6D_6 as external reference (100 MHz). (*) Represents solvent peaks.

Synthesis of 12. 1b (0.40 g, 0.24 mmol) was added to a solution of 1,4-dizazbicyclo[2.2.2]octane (DABCO, 1.33 gr, 11.9 mmol) in ethanol (8 mL). The addition was made in portions of 100 mg every 30 minutes. The resulting mixture was heated to 85°C in a pressure tube for 9 hours. After cooling to 25 °C, the solvent was removed under vacuum and the residue was washed with chloroform and filtered. The solid was dissolved in methanol and precipitated by the addition of diethyl ether. The precipitate was filtered, washed with diethyl ether, dissolved in water and concentrated to afford a white solid. Finally, the solid was dissolved in water and crystallized by vapor diffusion of isopropanol, to afford a white solid (444.8 mg, 66.7%). ^{1}H NMR (400 MHz, D_2O): δ 6.94 (s, Ar*H*, 10H), 4.53 & 4.31 (m, $ArOCH_2CH_2N$, 20H), 3.93 (s, $ArCH_2Ar$, 10H), 3.65-3.59 (br, $ArOCH_2CH_2N^+$ & $N^+(CH_2)_3(CH_2)_3N$, 80 H), 3.26 (t, J = 7.0 Hz, $N^+(CH_2)_3(CH_2)_3N$, 60 H) ppm. ^{13}C NMR (100 MHz, D_2O): δ 149.2, 129.4, 115.9, 63.3, 61.9, 53.0, 44.2, 30.2 ppm. HRMS: *m/z* Calcd. for $C_{115}H_{180}O_{10}N_{20}Br_{11}$ $[M+Br]^-$ 2875.5147, found 2875.5337. Anal. calcd. for $C_{115}H_{180}Br_{10}N_{20}O_{10}.21.25H_2O$: C, 43.37; H, 7.04; N, 8.8. Found: C, 43.16; H, 6.82; N, 8.71.

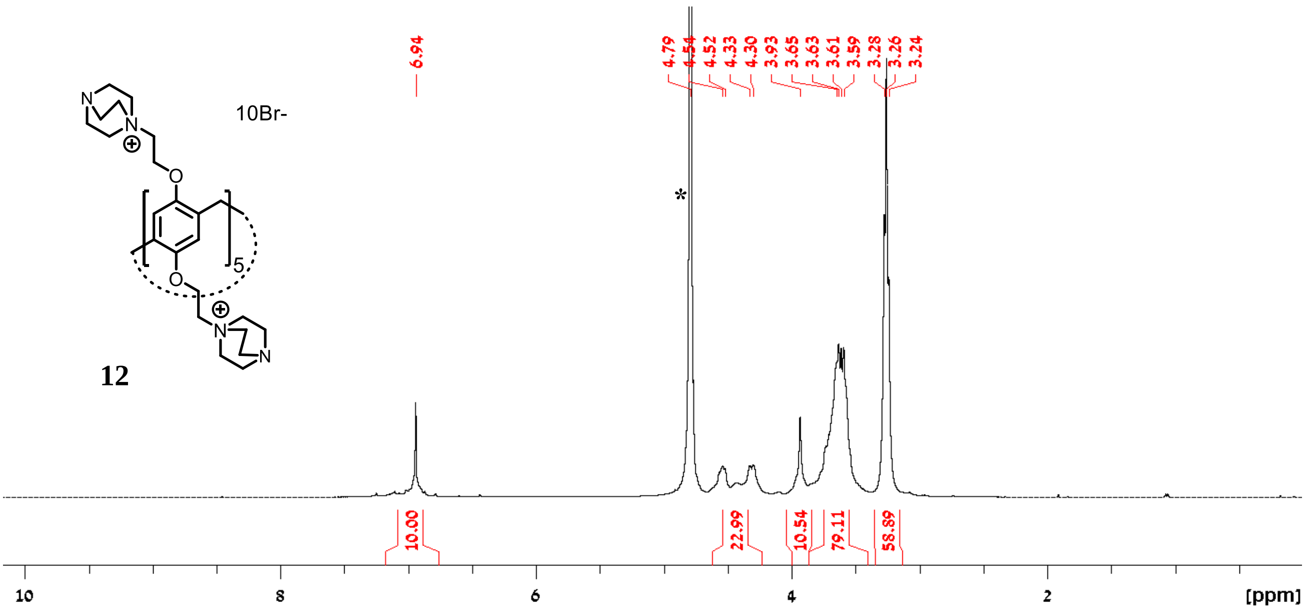

Figure S36. ^{1}H-NMR spectrum of **12** in D_2O (400 MHz). (*) Represents solvent peaks.

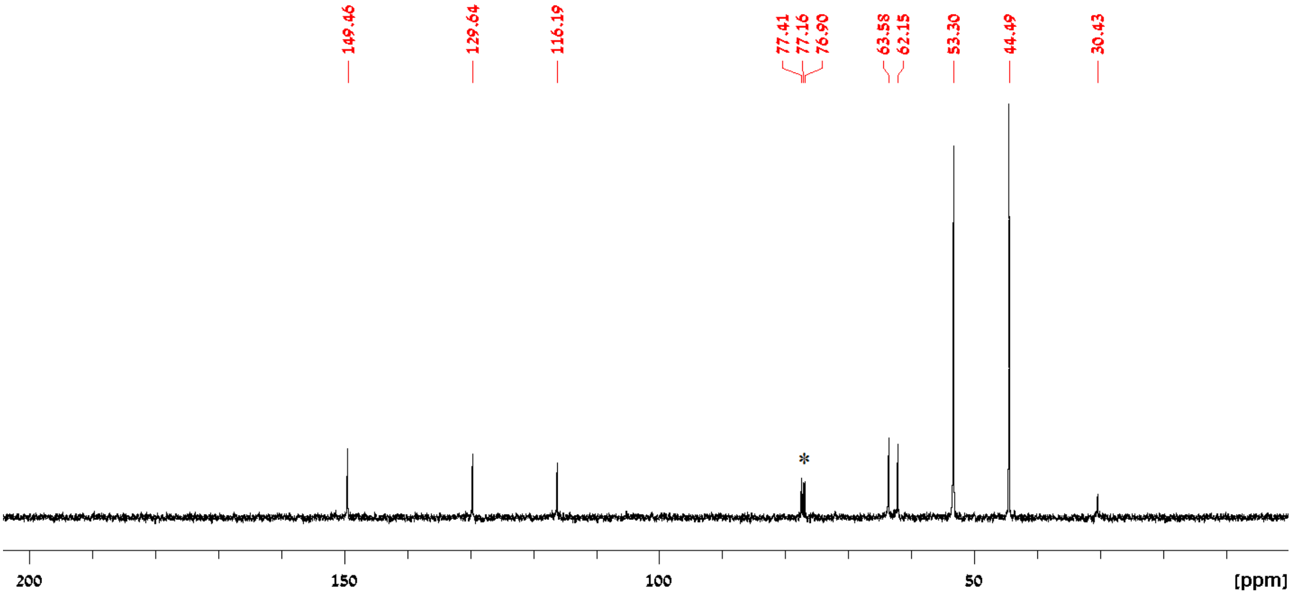

Figure S37. ^{13}C-NMR spectrum of **12** in D_2O and $CDCl_3$ as external reference (100 MHz). (*) Represents solvent peaks.

Synthesis of 13: Methyl iodide (0.44 mL, 7.1 mmol) was added to a solution of **12** (0.10 g, 0.04 mmol) in a water/acetonitrile mixture (2:8, 3.5 ml). The resulting mixture was heated to 60°c in a pressure tube for 4 days. After cooling to 25 °C, the product was precipitated by the addition of acetonitrile. The solvent was removed, and the residue was washed with another portion of acetonitrile. The resulting solid was dissolved in water and concentrated to afford a red-brown solid. Finally, the obtained solid was dissolved in water and crystallized by vapor diffusion of isopropanol, to afford a colorless solid (76 mg, 50%). ^{1}H NMR (400 MHz, D_2O): δ 7.08 (s, Ar*H*, 10H), 4.90 &

4.70 (m, ArOCH_2CH$_2$N, 20H), 4.50-4.39 (br, ArOCH$_2$CH_2N$^+$ & N$^+$(CH_2)$_3$(CH$_2$)$_3$N, 80 H), 4.18-4.17 (br, N$^+$(CH$_2$)$_3$(CH_2)$_3$N, 60 H), 3.96 (s, ArCH_2Ar, 10H), 3.46 (s, N$^+$(CH$_2$)$_3$(CH$_2$)$_3$NCH_3, 30H) ppm. ^{13}C NMR (100 MHz, D$_2$O): δ 148.5, 128.6, 115.8, 65.0, 61.9, 53.6, 52.9, 52.6, 30.0 ppm. Anal. calcd. for $C_{125}H_{210}Br_{10}I_{10}N_{20}O_{10}$.27.7$H_2O$: C, 31.81; H, 5.67; N, 5.93. Found: C, 31.52; H, 5.38; N, 5.71.

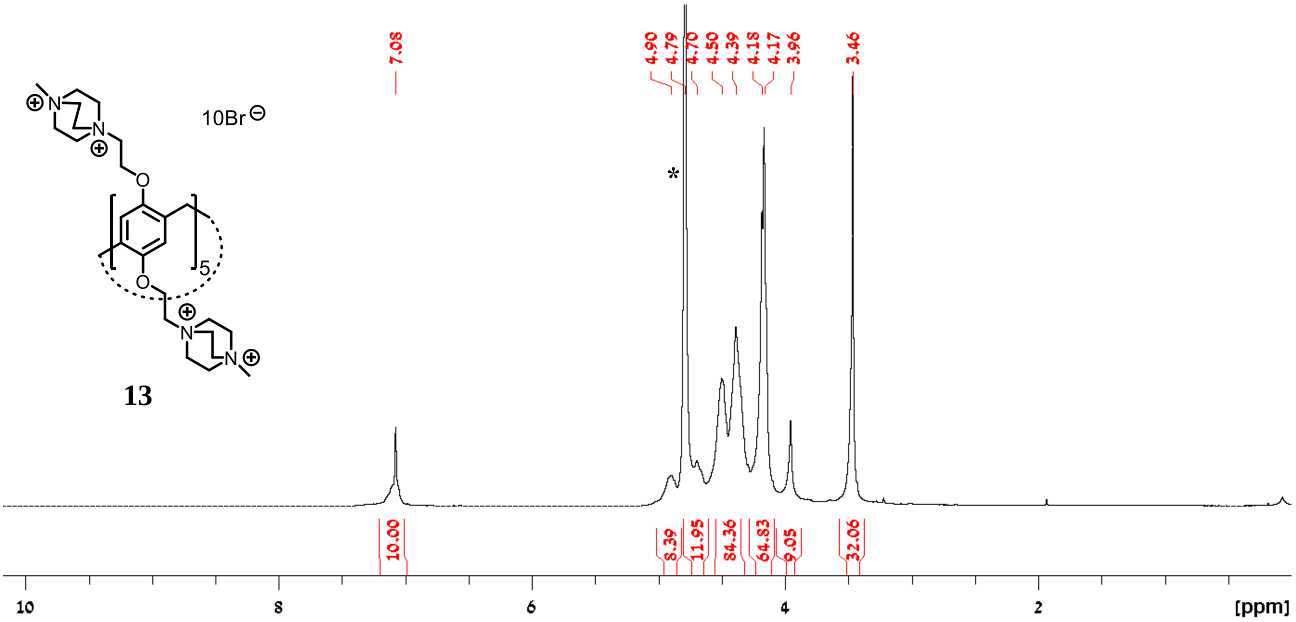

Figure S38. ^{1}H-NMR spectrum of **13** in D$_2$O (400 MHz). (*) Represents solvent peaks.

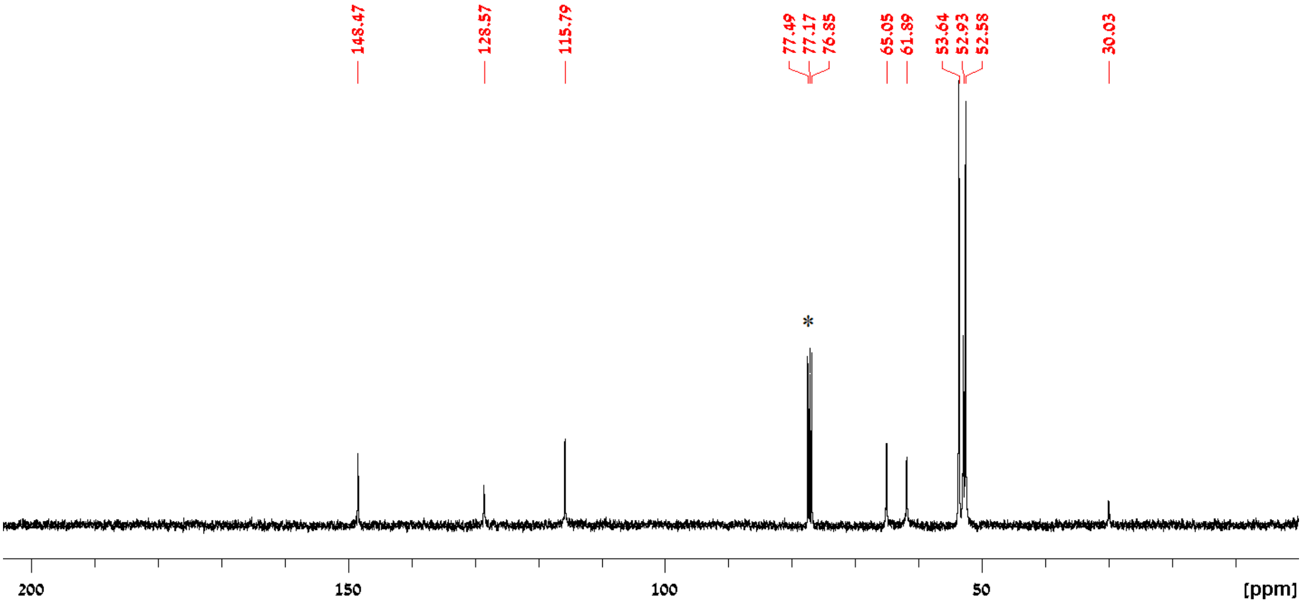

Figure S39. ^{13}C-NMR spectrum of **13** in D$_2$O and CDCl$_3$ as external reference (100 MHz). (*) Represents solvent peaks.

Synthesis of 4a. A mixture of hydroquinone (20 gr, 0.18 mol) and potassium Hydroxide (20.4 g, 0.36 mmol) was refluxed in ethanol (420 mL). A solution of 1-bromodecane (30mL, 0.14 mol) in 30mL ethanol was added dropwise and the reaction was kept at reflux for 24 hours. After cooling to 25°C, 1N HCl solution (250 mL) was added into the mixture and the product was extracted with DCM (3 × 75 mL). The combined organic layers were washed with brine, dried with Na_2SO_4 and the solvent was evaporated. The obtained product was purified by recrystallization from hexane to afford white crystals (20.1 gr, 44%). ^{1}H NMR (400 MHz, $CDCl_3$): δ 6.77 (m, Ar*H*, 4H), 3.91 (t, *J* = 6.6 Hz, $ArOCH_2(CH_2)_8CH_3$, 2H), 1.76 (quint, *J* = 7.0 Hz, $ArOCH_2CH_2(CH_2)_7CH_3$, 2H), 1.45 (m, $ArO(CH_2)_2CH_2(CH_2)_6CH_3$, 2H), 1.37-1.29 (m, $ArO(CH_2)_3(CH_2)_6CH_3$, 12H), 0.90 (t, *J* = 6.9 Hz, $ArO(CH_2)_9CH_3$, 3H) ppm. ^{13}C NMR (100 MHz, $CDCl_3$): δ 153.4, 149.4, 116.1, 115.7, 68.9, 32.0, 29.7, 29.7, 29.5, 29.4, 29.4, 26.1, 22.8, 14.2 ppm.

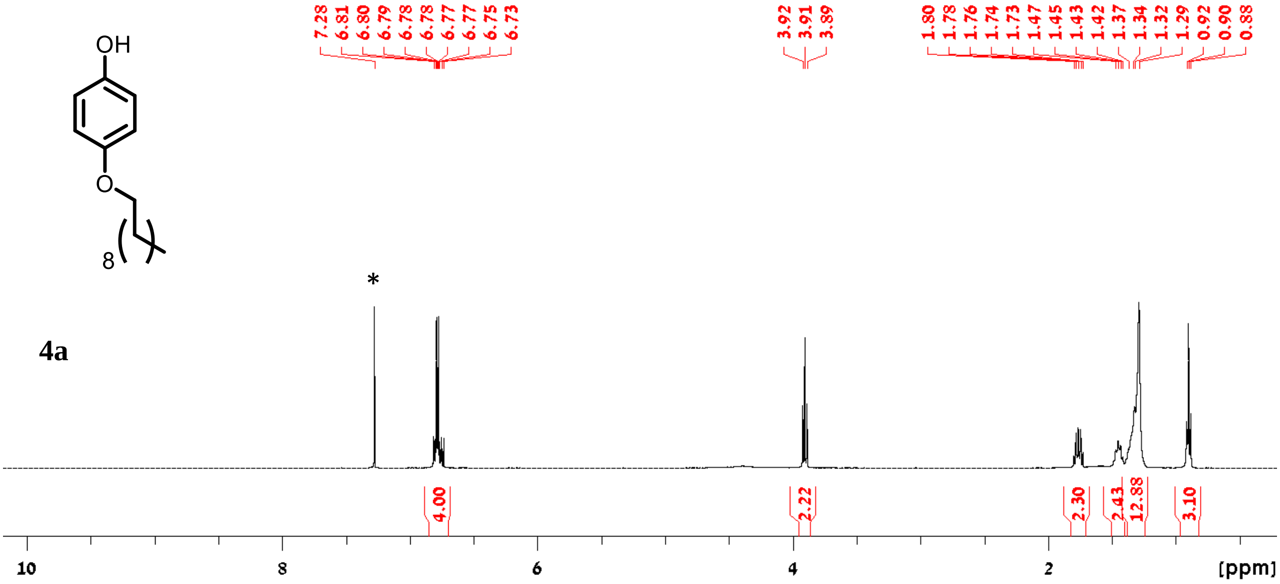

Figure S40. ^{1}H-NMR spectrum of **4a** in $CDCl_3$ (400 MHz). (*) Represents solvent peaks.

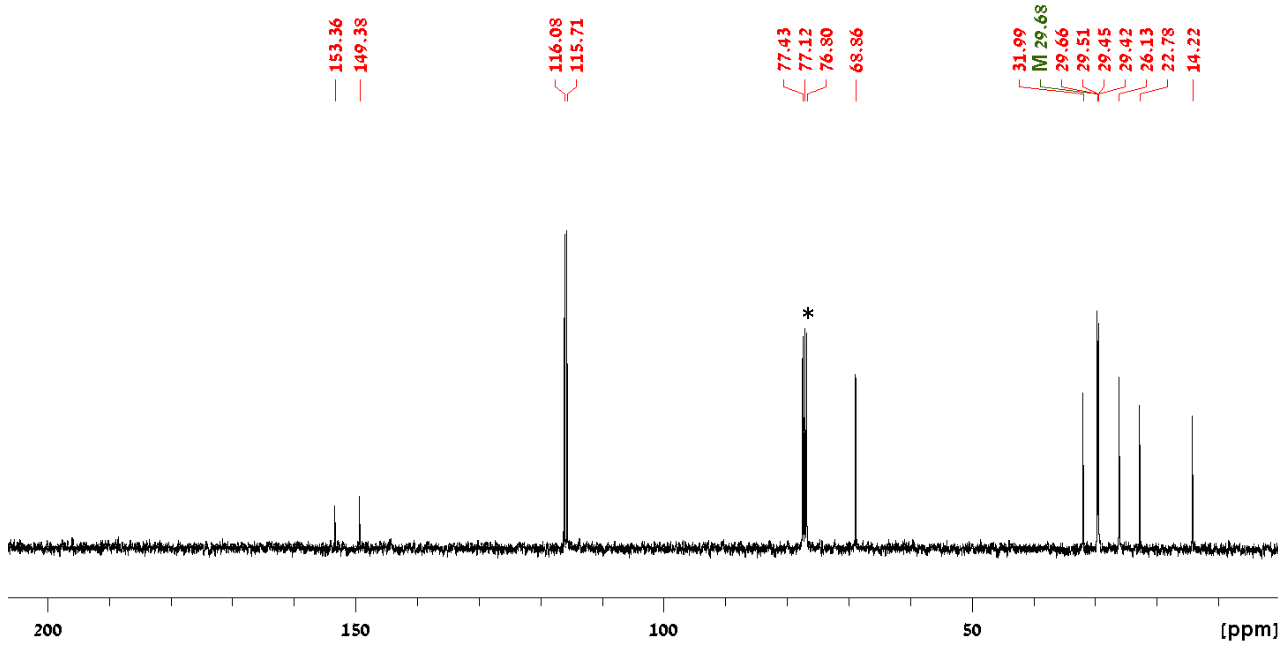

Figure S41. ^{13}C-NMR spectrum of **4a** in $CDCl_3$ (100 MHz). (*) Represents solvent peaks.

Synthesis of 4b. A mixture of 4-(decamethoxy)phenol (**4a**) (15.5 gr, 61.9 mmol), 1,2-dibromoethane (26.6 mL, 309.5 mmol) and potassium carbonate (21.39 gr, 154.8 mmol) was refluxed for 24 hours in acetonitrile (150 mL). After cooling to 25°C, the reaction mixture was filtered through celite and the solvent was evaporated under vacuum. the obtained product was purified by column chromatography (silica gel; hexane/EtOAc) to afford a white solid (6.5 gr, 29%). ^{1}H NMR (400 MHz, $CDCl_3$): δ 6.84 (m, Ar*H*, 4H), 4.24 (t, *J* = 6.4 Hz, $ArOCH_2CH_2Br$, 2H), 3.90 (t, *J* = 6.6 Hz, $ArOCH_2(CH_2)_8CH_3$, 2H), 3.61 (t, *J* = 6.2 Hz, $ArOCH_2CH_2Br$, 2H), 1.75 (quint, *J* = 7.0 Hz, $ArOCH_2CH_2(CH_2)_7CH_3$, 2H), 1.44 (m, $ArO(CH_2)_2CH_2(CH_2)_6CH_3$, 2H), 1.30-1.27 (m, $ArO(CH_2)_3(CH_2)_6CH_3$, 12H), 0.88 (t, *J* = 6.7 Hz, $ArO(CH_2)_9CH_3$, 3H) ppm. ^{13}C NMR (100 MHz, $CDCl_3$): δ 154.0, 152.1, 116.1, 115.5, 68.8, 68.7, 32.0, 29.7, 29.7, 29.5, 29.5, 29.5, 29.5, 26.1, 22.8, 14.2 ppm. HRMS: m/z Calcd. for $C_{18}H_{29}O_2Br$ $[M]^+$ 356.1351, found 356.1359.

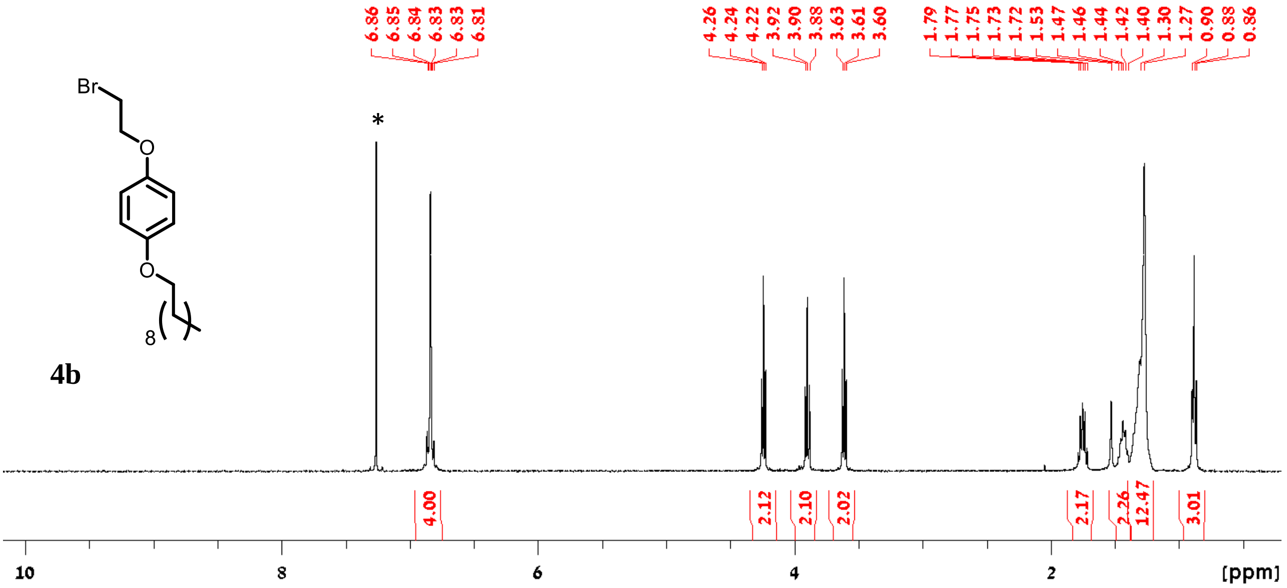

Figure S42. ^{1}H-NMR spectrum of **4b** in $CDCl_3$ (400 MHz). (*) Represents solvent peaks.

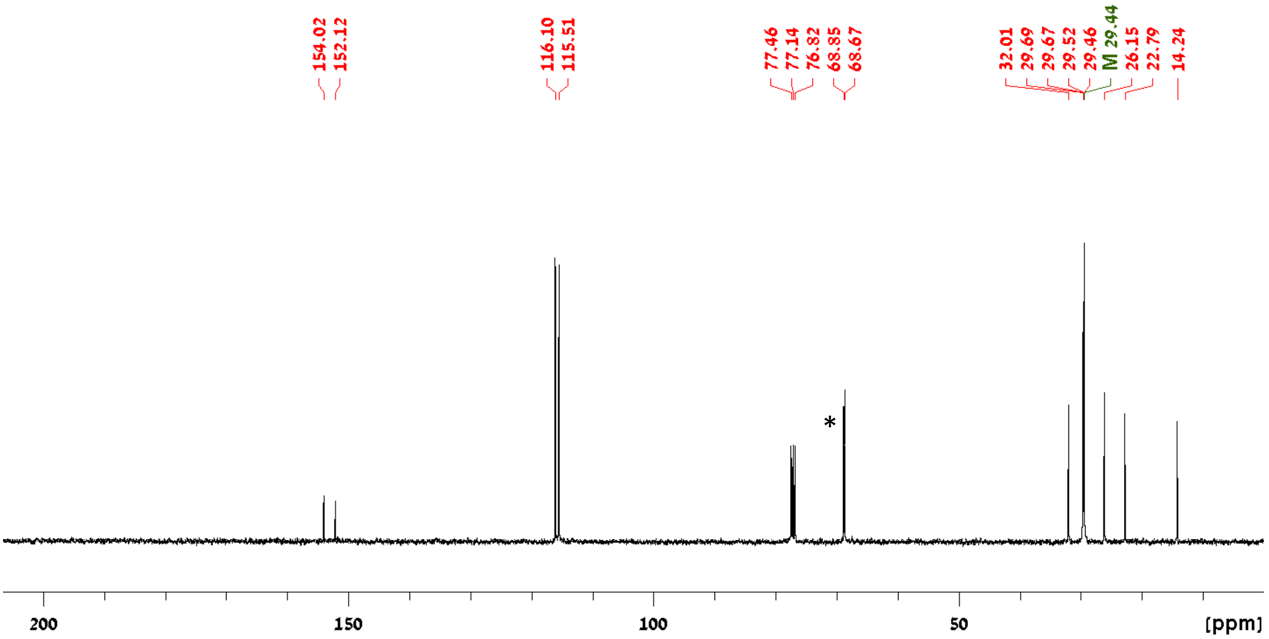

Figure S43. ^{13}C-NMR spectrum of **4b** in $CDCl_3$ (100MHz). (*) Represents solvent peaks.

Synthesis of 4c. To a solution of **4b** (1 gr, 2.8 mmol) and paraformaldehyde (250 mg, 8.3 mmol) in 1,2-dichloroethane (46 mL) was added $BF_3{\cdot}OEt_2$ (0.39 mL, 3.16 mmol). The reaction mixture was kept at RT for 4.5 hours under argon atmosphere. The reaction mixture was washed with water (2 × 100mL), brine (100mL) and dried with sodium sulfate and concentrated *in vacuo*. The product was purified by chromatography (silica gel; eluent: petroleum ether: dichloromethane) to afford a light yellow solid consisting of all the four isomers (0.83 g, 16%). ^{1}H-NMR (400 MHz, $CDCl_3$): δ 6.92-

6.78 (multiple s, Ar*H*, 10H), 4.18-4.10 (m, ArO*CH₂*CH₂Br, 10H), 3.91-3.88 (m, ArO*CH₂*(CH₂)₈CH₃, 10H), 3.81-3.76 (multiple s, Ar-*CH₂*-Ar, 10H), 3.61-3.55 (m, ArOCH₂*CH₂*Br, 10H), 1.83-1.80 (m, ArOCH₂*CH₂*(CH₂)₇CH₃, 10H), 1.55-1.52 (m, ArO(CH₂)₂*CH₂*(CH₂)₆CH₃, 10H), 1.37-1.15 (m, ArO(CH₂)₃*(CH₂)₆*CH₃, 60H), 0.88-0.84 (m, ArO(CH₂)₉*CH₃*, 15H) ppm. ^{13}C-NMR (125 MHz, $CDCl_3$): δ 150.7, 149.1, 149.0, 129.1, 128.9, 128.8, 128.7, 128.6, 128.5, 128.5, 116.3, 116.1, 115.9, 115.7, 115.2, 115.1, 115.0, 114.8, 114.7, 114.6, 69.2, 69.1, 68.9, 68.7, 68.4, 32.1, 30.9, 30.6, 30.3, 30.0, 29.9, 29.8, 29.6, 29.5, 29.5, 26.5, 26.4, 22.8, 14.3 ppm .

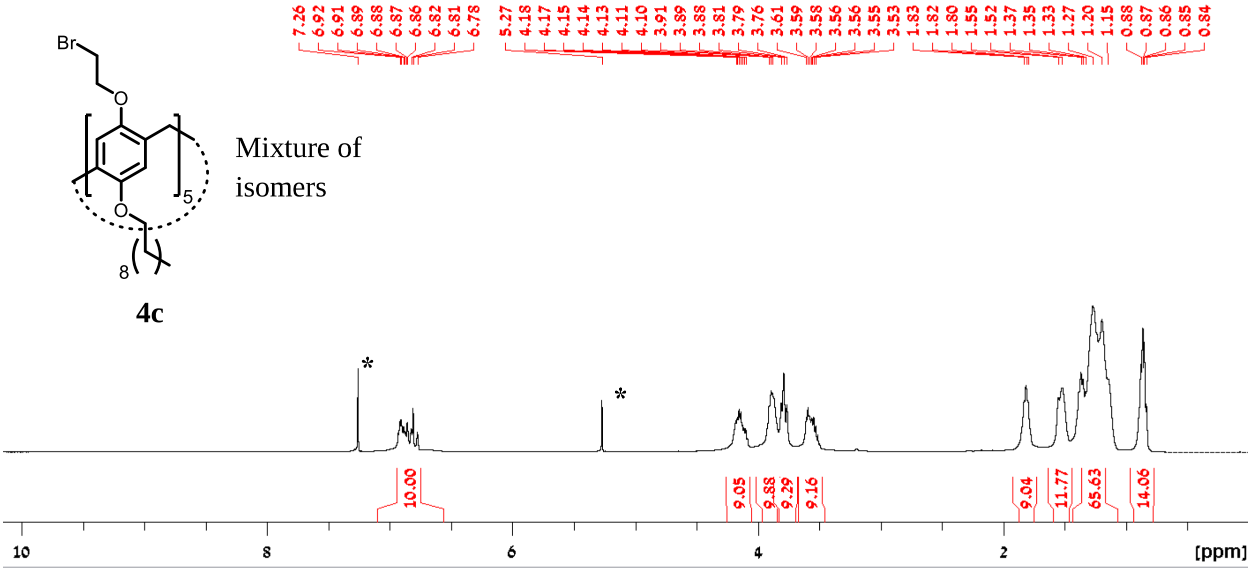

Figure S44. ¹H-NMR spectrum of **4c** in $CDCl_3$ (400 MHz). (*) Represents solvent peaks.

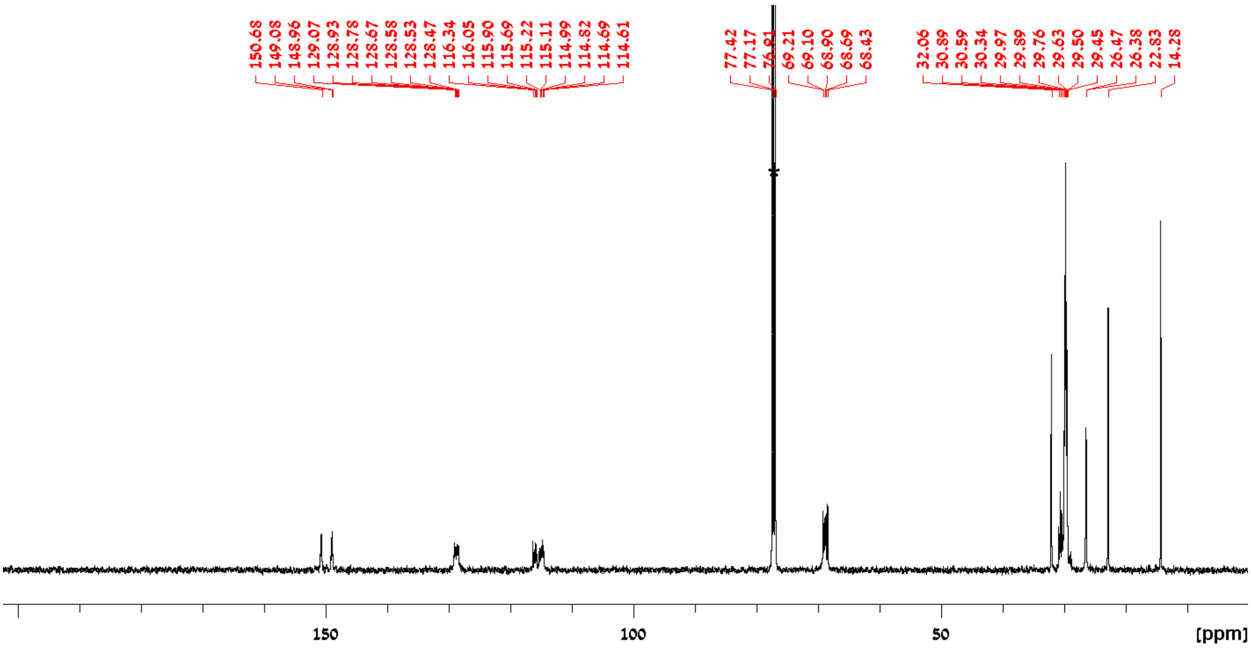

Figure S45. ^{13}C-NMR spectrum of **4c** in $CDCl_3$ (125MHz). (*) Represents solvent peaks.

Synthesis of 4d. Compound **4d** was prepared similarly to compound **4c** by reacting **4b** (6.5 gr, 18.2 mmol) with paraformaldehyde (1.63 gr, 54.3 mmol) and $BF_3{\cdot}OEt_2$ (2.51 mL, 20.1 mmol) in 1,2-dichloroethane (300 mL). The symmetrical isomer **4d** was isolated by chromatography (silica gel; eluent: petroleum ether: dichloromethane) (0.42 g, 6.3%). ^{1}H-NMR (500 MHz, $CDCl_3$): δ 6.93 (s, Ar*H*, 5H), 6.83 (s, Ar*H*, 5H), 4.19 (t, *J* = 5.6 Hz, ArO$\mathit{CH_2}$$CH_2$Br, 10H), 3.88 (t, *J* = 6.4 Hz, ArO$\mathit{CH_2}$$(CH_2)_8CH_3$, 10H), 3.80 (s, Ar-$\mathit{CH_2}$-Ar, 10H), 3.61 (t, *J* = 5.8 Hz, ArO$CH_2$$\mathit{CH_2}$Br, 10H), 1.83 (quint, *J* = 7.1 Hz, ArO$CH_2$$\mathit{CH_2}$$(CH_2)_7CH_3$, 10H), 1.54 (m, ArO$(CH_2)_2$$\mathit{CH_2}$$(CH_2)_6CH_3$, 10H), 1.41-1.17 (m, ArO$(CH_2)_3$$(\mathit{CH_2})_6$$CH_3$, 60H), 0.86 (t, *J* = 7.2 Hz, ArO$(CH_2)_9$$\mathit{CH_3}$, 15H) ppm. ^{13}C-NMR (125 MHz, $CDCl_3$): δ 150.7, 149.1, 129.0, 128.5, 116.3, 114.7, 69.2, 68.4, 32.0, 30.9, 30.0, 29.8, 29.8, 29.7, 29.5, 29.5, 26.5, 22.8, 14.3 ppm. Anal. calcd. for $C_{95}H_{145}Br_5O_{10}$: C, 61.79; H, 7.91. Found: C, 61.91; H, 7.77. HRMS: m/z Calcd. for $C_{95}H_{145}O_{10}BrNa$ $[M+ Na]^+$ 1865.6643, found 1865.6632.

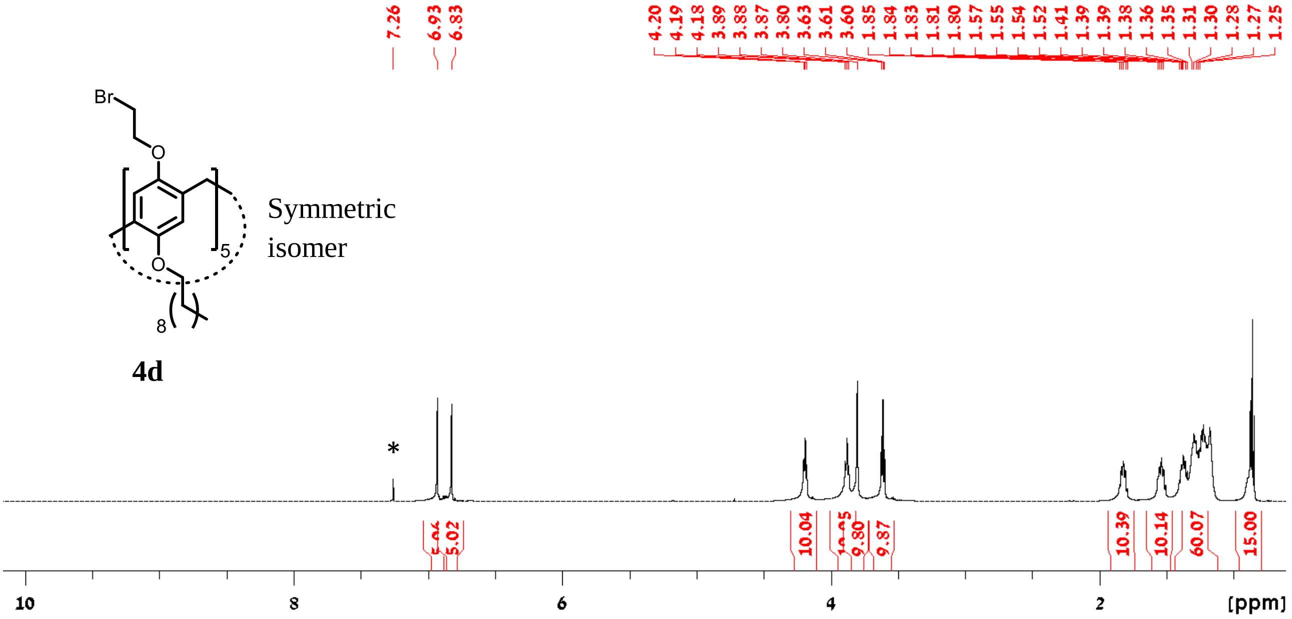

Figure S46. ^{1}H-NMR spectrum of **4d** in $CDCl_3$ (500 MHz). (*) Represents solvent peaks.

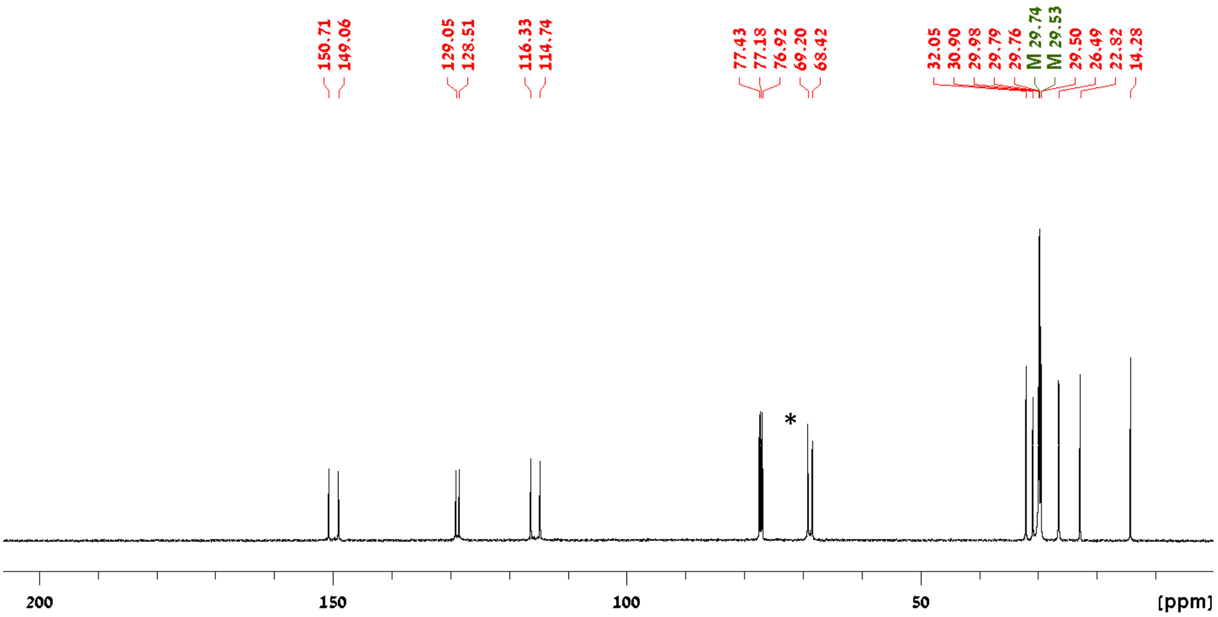

Figure S47. ^{13}C-NMR spectrum of **4d** in $CDCl_3$ (125MHz). (*) Represents solvent peaks.

Synthesis of 14a. Trimethylamine (33% in ethanol, 0.64 mL, 2.7 mmol) was added to a solution of **4c** (0.2 g, 0.11 mmol) in ethanol (8.0 mL). The reaction mixture was heated to 82°C for 5 days in a pressure tube. After cooling to 25°C the solvent was evaporated to obtain a yellow solid (0.2 g, 88%). ^{1}H-NMR (500 MHz, methanol-d_4): δ 7.06-7.51 (multiple s, Ar*H*, 10H), 4.59-4.44 (m, ArO$CH_2$$CH_2$N, 10H), 4.11-4.02 (m, ArO$CH_2$$CH_2$N, 10H), 3.86-3.75 (m, Ar-CH_2-Ar & ArO$CH_2$$(CH_2)_8CH_3$, 20H), 3.42-3.19 (multiple s, N$(CH_3)_3$, 45H), 1.84-1.77 (m, ArO$CH_2$$CH_2$$(CH_2)_7CH_3$, 10H), 1.55-1.15 (m, ArO$(CH_2)_2$$(CH_2)_7$$CH_3$, 70H), 0.91-0.86 (m, ArO$(CH_2)_9$$CH_3$, 15H). ^{13}C-NMR (100 MHz, methanol-d_4): 152.5, 149.9, 149.8, 148.8, 149.6, 149.5, 149.2, 130.4, 130.1, 130.0, 129.8, 117.2, 116.8, 116.5, 70.5, 70.3, 70.0, 69.8, 66.8, 64.9, 64.5, 64.1, 54.9, 54.8, 33.2, 31.0, 30.9, 30.7, 30.6, 27.8, 27.7, 27.5, 27.4, 27.2, 23.8, 14.5 ppm. HRMS: m/z Calcd. for $C_{110}H_{190}N_5O_{10}Br_6$ $[M+Br]^-$ 2216.9592, found 2216.9585.

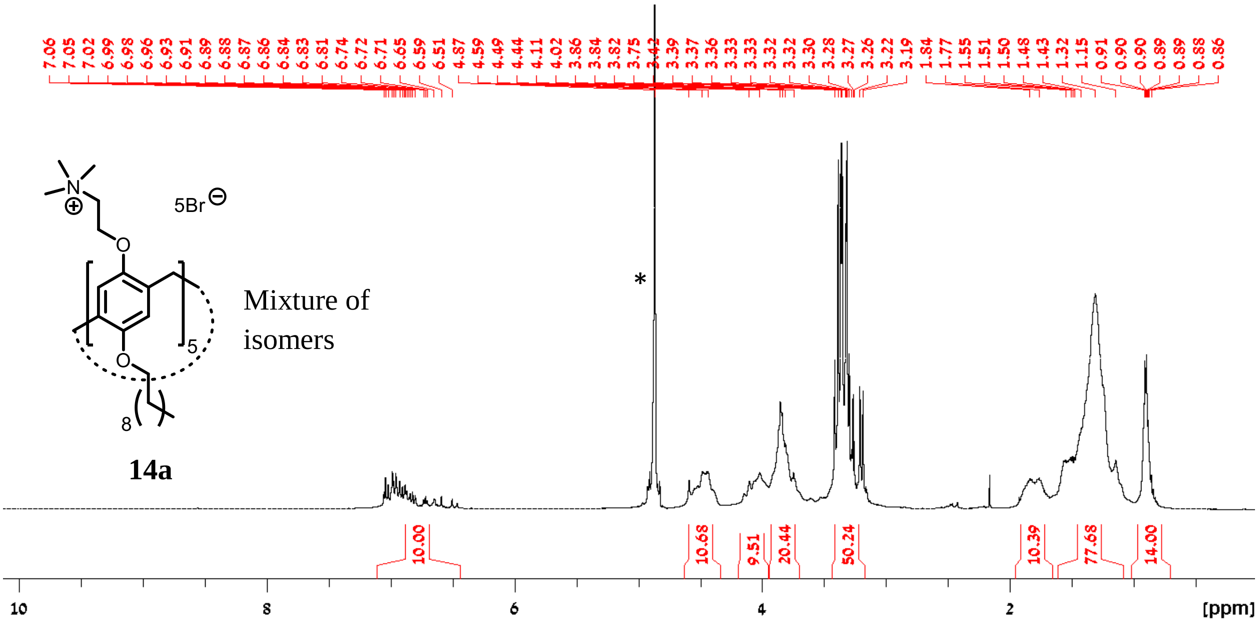

Figure S48. ^{1}H-NMR spectrum of **14a** in methanol-d_4 (500 MHz). (*) Represents solvent peaks.

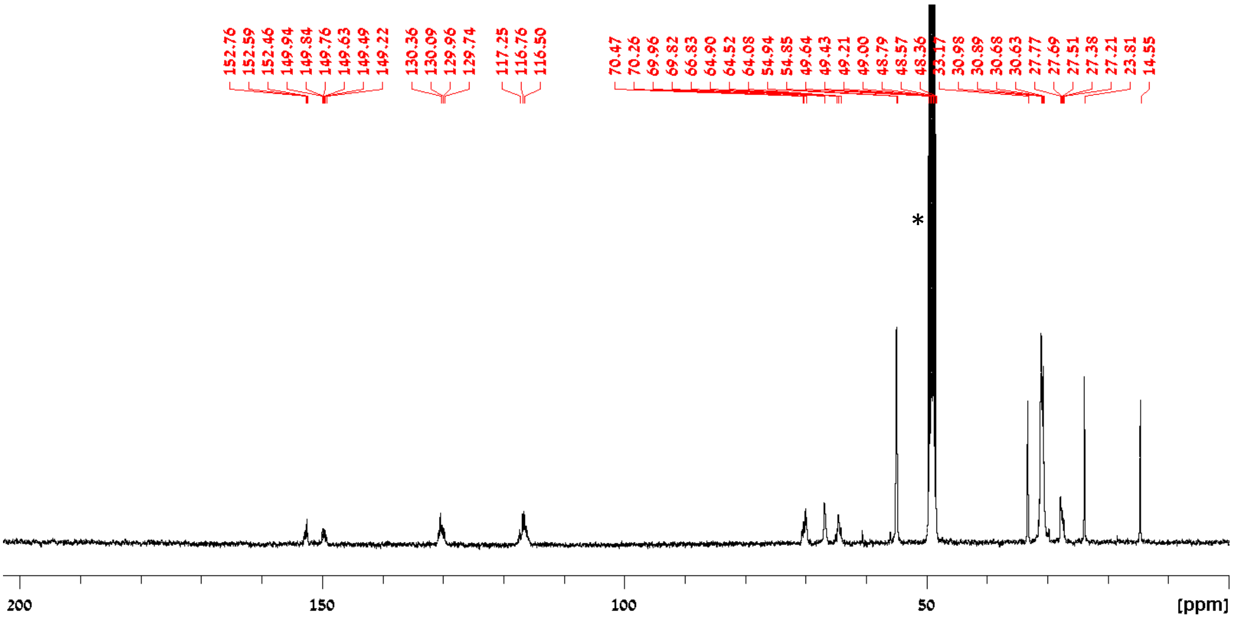

Figure S49. ^{13}C-NMR spectrum of **14a** in methanol-d_4 (100 MHz). (*) Represents solvent peaks.

Synthesis of 14b. Compound **14b** was prepared similarly to compound **14a** by reacting **4d** (0.12 g, 0.06 mmol) with Trimethylamine (33% in ethanol, 0.34 mL, 1.9 mmol) in ethanol (5.0 mL) for 24 hours. After cooling to 25°C the solvent was evaporated and the obtained solid was dissolved in water (5 mL) and then filtered. The filtrate was evaporated and the solid was recrystallized from ethanol to afford a white solid white solid (0.13 g, 94%). ^{1}H-NMR (400 MHz, methanol-d_4): δ 6.93 (s, Ar*H*, 5H), 6.81 (s, Ar*H*, 5H), 4.47 (br, ArO*CH*$_2$CH$_2$N, 10H), 4.04 (br, ArOCH$_2$*CH*$_2$N, 10H), 3.84 (s, Ar-*CH*$_2$-Ar, 10H), 3.69 (br, ArO*CH*$_2$(CH$_2$)$_8$CH$_3$, 10H), 3.35 (s, N(*CH*$_3$)$_3$, 45H) 1.75 (br,

ArOCH$_2$*CH$_2$*(CH$_2$)$_7$CH$_3$, 10H), 1.52 (br, ArO(CH$_2$)$_2$*CH$_2$*(CH$_2$)$_6$CH$_3$, 10H), 1.41-1.28 (m, ArO(CH$_2$)$_3$(*CH$_2$*)$_6$CH$_3$, 60H), 0.89 (t, J = 7.0 Hz, ArO(CH$_2$)$_9$*CH$_3$*, 15H) ppm. ^{13}C-NMR (100 MHz, methanol-d$_4$): δ 151.1, 148.7, 129.0, 128.6, 115.2, 115.1, 68.5, 65.8, 63.1, 53.6, 31.9, 29.9, 29.8, 29.8, 29.7, 29.5, 26.6, 22.6, 13.3 ppm. HRMS: m/z Calcd. for $C_{110}H_{190}N_5O_{10}Br_4$ [M-Br]$^+$ 2062.1284, found 2062.1239. Anal. calcd. for $C_{110}H_{190}Br_5N_5O_{10}$.7.05$H_2O$: C, 58.22; H, 9.07; N, 3.09. Found: C, 57.88; H, 8.72; N, 2.96.

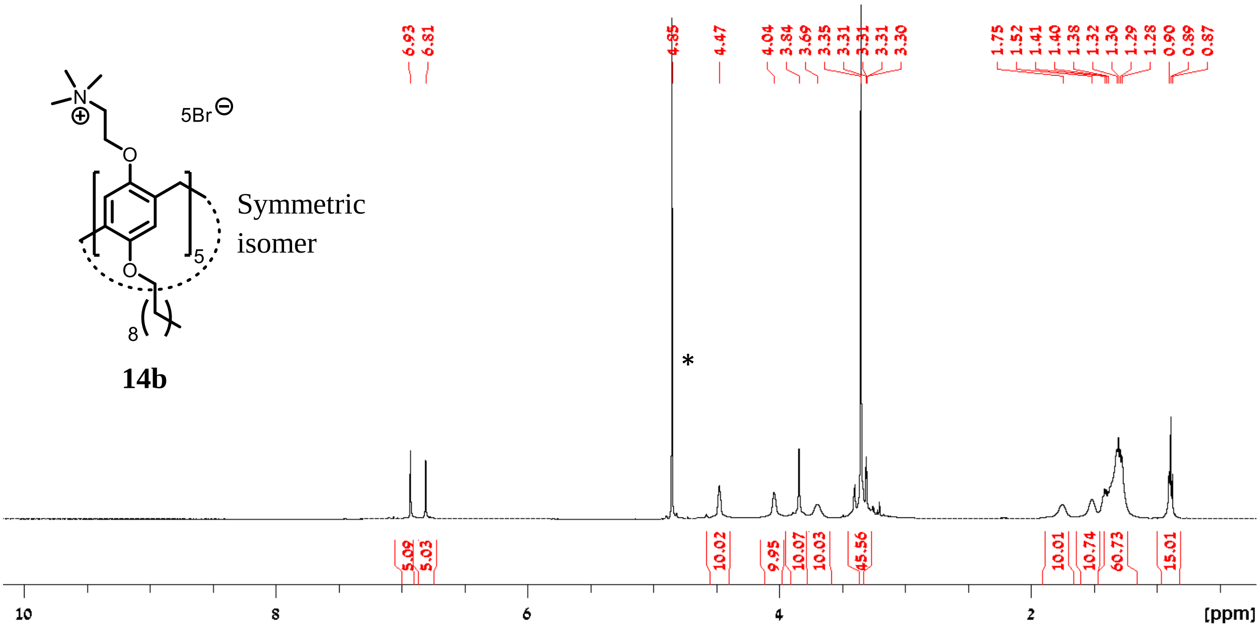

Figure S50. ^{1}H-NMR spectrum of **14b** in methanol-d$_4$ (400 MHz). (*) Represents solvent peaks.

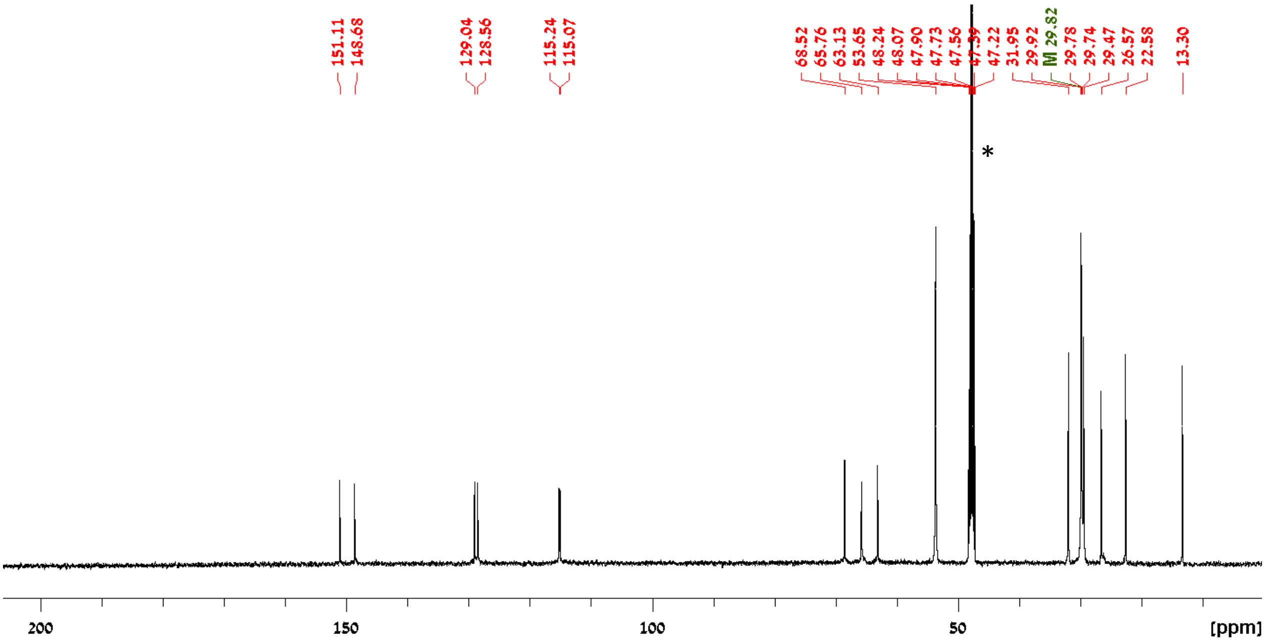

Figure S51. ^{13}C-NMR spectrum of **14b** in methanol-d$_4$ (100MHz). (*) Represents solvent peaks.

Synthesis of 15a. 1,4-diazabicyclo[2.2.2]octane (DABCO) (340 mg, 3.0 mmol) was added to a solution of 1,12-dibromododecane (1.0 g, 3.0 mmol) in ethyl acetate (6 mL) under vigorous stirring. The reaction mixture was kept at room temperature for 24 hr., followed by precipitation. The precipitate was filtered, washed with diethyl ether and dried under vacuum to afford **15a** as a white powder (1.15 g, 87%). ^{1}H NMR (400 MHz, D_2O): 3.55 (t, *J* =6.8 Hz, $\mathrm{BrC}\mathit{H}_2$, 2H), 3.41 (t, *J* = 7.4 Hz, $\mathrm{N^+(C}\mathit{H}_2\mathrm{)_3(C}\mathit{H}_2\mathrm{)_3N}$, 6H), 3.23 (m, $\mathrm{C}\mathit{H}_2\mathrm{N^+(CH_2)_3(CH_2)_3N}$ & $\mathrm{N^+(CH_2)_3(C}\mathit{H}_2\mathrm{)_3N}$, 8H), 1.87 (quin, *J* = 7.0 Hz, $\mathrm{BrCH_2C}\mathit{H}_2$, 2H), 1.77 (m, DABCO-$\mathrm{CH_2C}\mathit{H}_2$, 2H), 1.37 (m, $\mathrm{CH_2(C}\mathit{H}_2\mathrm{)_8\ CH_2Br}$, 16H) ppm. ^{13}C NMR (100 MHz, D_2O): δ 64.8, 52.4, 44.5, 35.2, 33.1, 29.8, 29.7, 29.7, 29.6, 29.2, 29.0, 28.4, 26.5, 21.9 ppm.

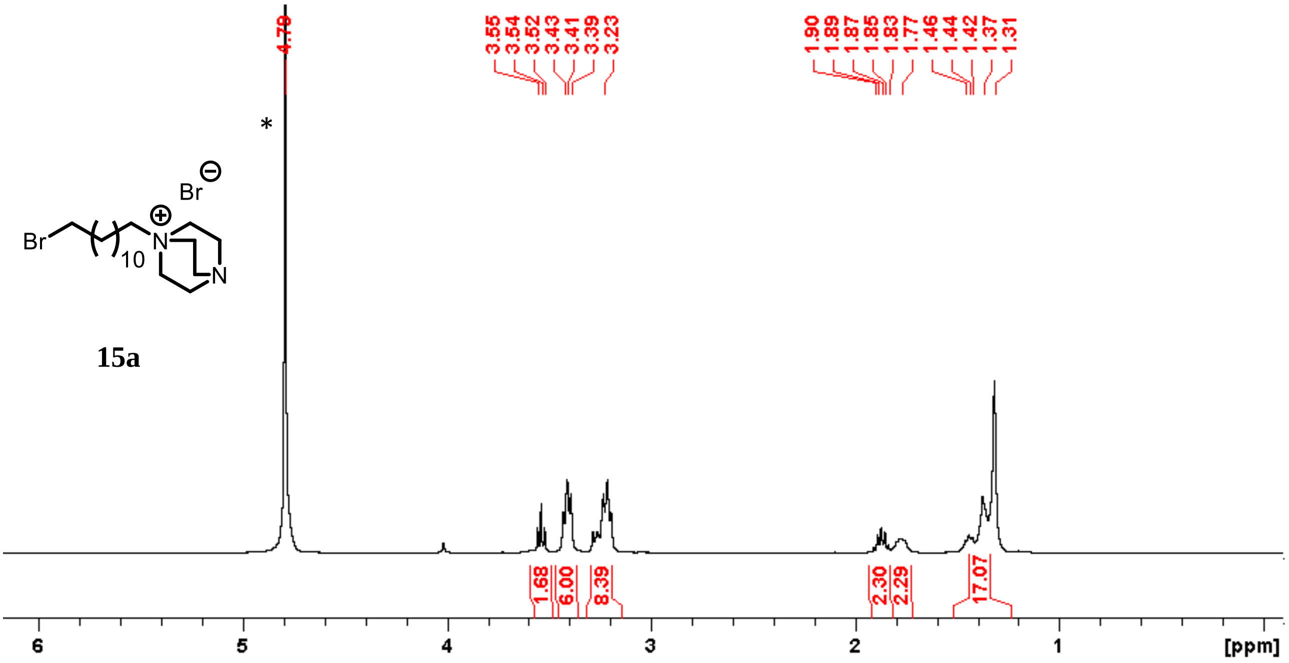

Figure S52. ^{1}H-NMR spectrum of **15a** in D_2O (400 MHz). (*) Represents solvent peaks.

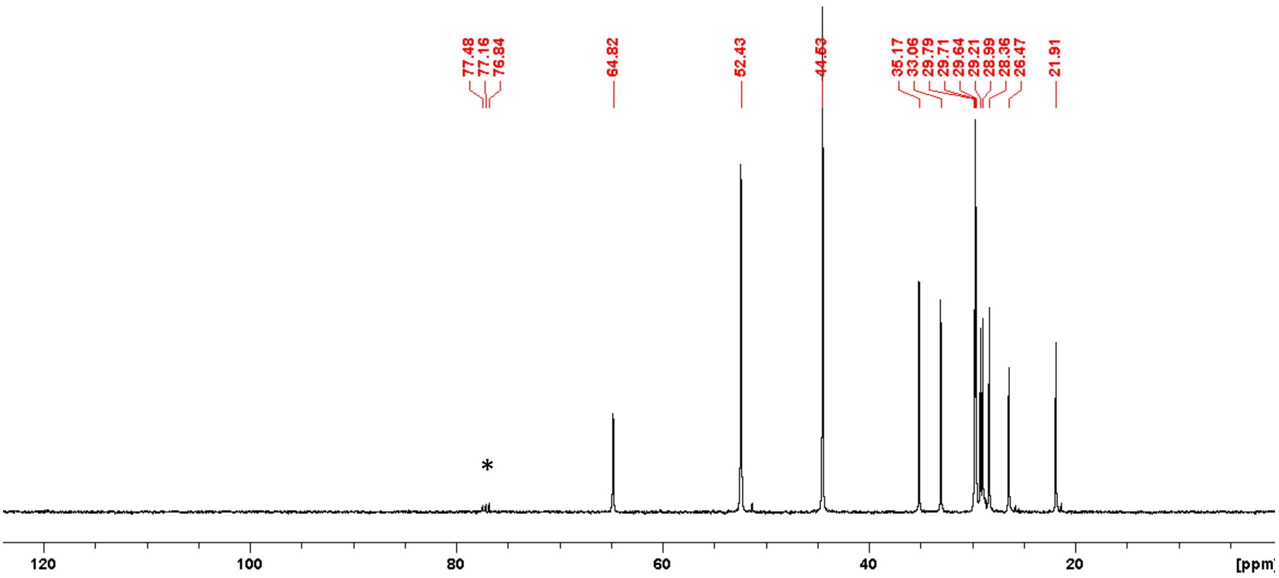

Figure S53. ^{13}C-NMR spectrum of **15a** in D_2O (100MHz). (*) Represents solvent peaks.

Synthesis of 15. To a solution of 1,4-diazabicyclo[2.2.2]octane (DABCO) (292 mg, 2.6 mmol) and **1** (300 mg, 0.13 mmol) in D_2O (3 mL) was added **15a** (290 mg, 0.65 mmol) under vigorous stirring. The addition was made in portions of 58 mg every 24 hours during 5 days. Chloroform (3mL) was added and stirring continued for 1 hour. Aqueous phase was concentrated to afford light yellow powder. Finally, the solid was dissolved in H_2O and crystallized by vapor diffusion of isopropanol, to afford a white solid (160 mg, 44%). ^{1}H NMR (500 MHz, D_2O): 7.05 (s, Ar*H*, 10H), 4.64 (br, ArOCH_2CH_2, 20H), 4.02 (br, ArOCH_2CH_2N, 20H), 3.88 (s, ArCH_2Ar, 10H), 3.34 (br, N^+(CH_2)$_3$(CH_2)$_3$N, 12H), 3.33 (s, NCH_3, 90H), 3.21 (br, C$H_2$$N^+$(C$H_2$)$_3$(C$H_2$)$_3$N & N^+(CH_2)$_3(CH_2)_3$N, 16H), 1.07 (br, N^+(CH_2)$_3$(CH_2)$_3$NCH_2CH_2, *4H),* 0.30 (br, N^+(CH_2)$_3$(CH_2)$_3$N(CH_2)$_2$(CH_2)$_2$, 8H), -0.09 (br, N^+(CH_2)$_3$(CH_2)$_3$N(CH_2)$_4$CH_2, 4H), -0.46 (br, N^+(CH_2)$_3$(CH_2)$_3$N(CH_2)$_5$CH_2, 4H) ppm. ^{13}C NMR (125 MHz, D_2O): δ 149.0, 130.0, 116.5, 65.0, 63.7, 54.5, 52.8, 44.7, 30.3, 30.0, 29.7, 29.4, 26.8. 22.3 ppm. Anal. calcd. for $C_{109}H_{198}Br_{12}N_{14}O_{10}\cdot 0.8C_3H_8O\cdot 14.3H_2O$: C, 42.76; H, 7.50; N, 6.27. Found: C, 42.73; H, 7.56; N, 6.32.

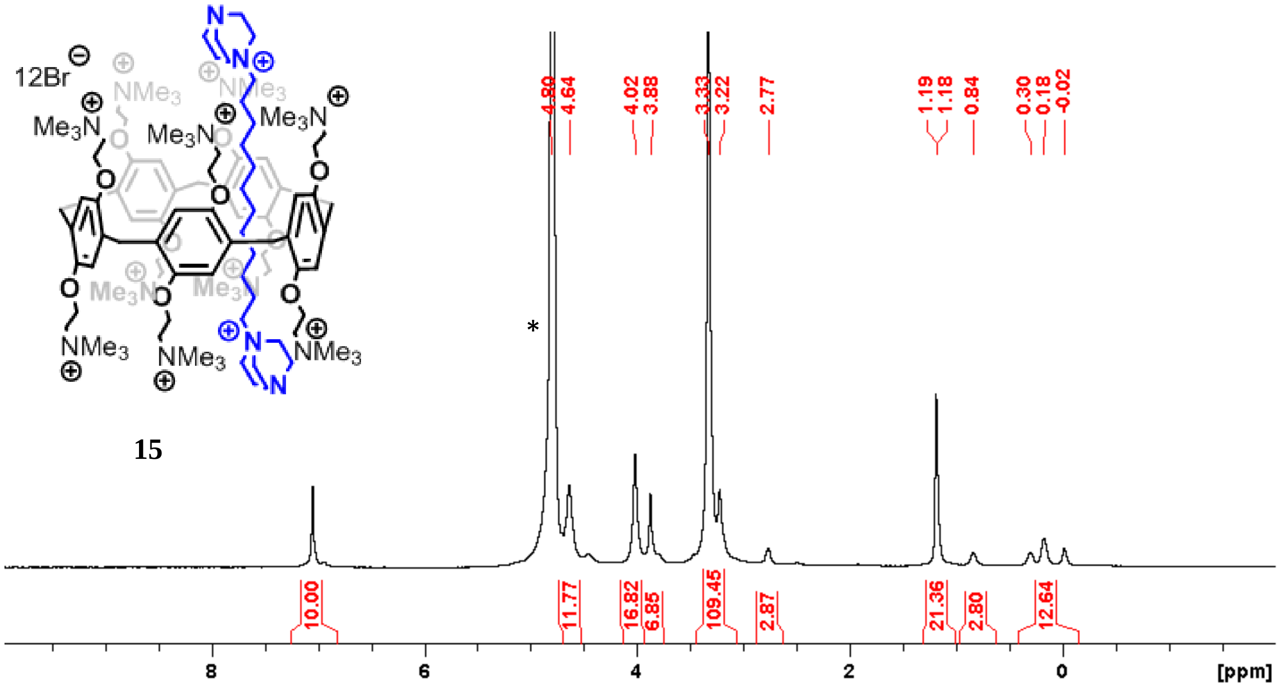

Figure S54. ^{1}H-NMR spectrum of **15** in D_2O (500 MHz). (*) Represents solvent peaks.

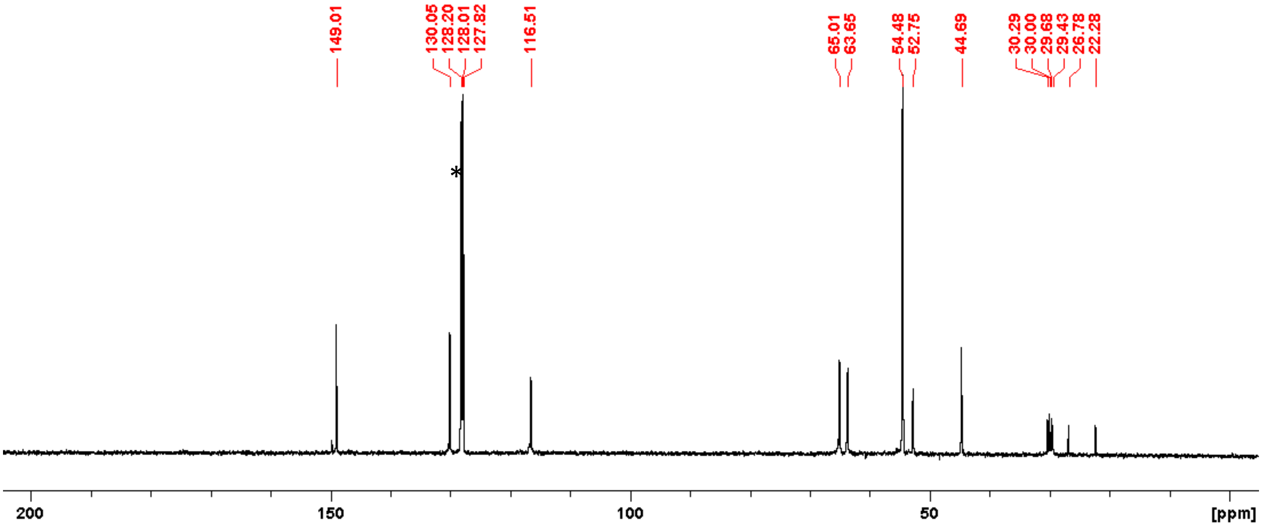

Figure S55. ^{13}C-NMR spectrum of **15** in D_2O (125 MHz). (*) Represents solvent peaks.

3. Results of selected examples from previous work.

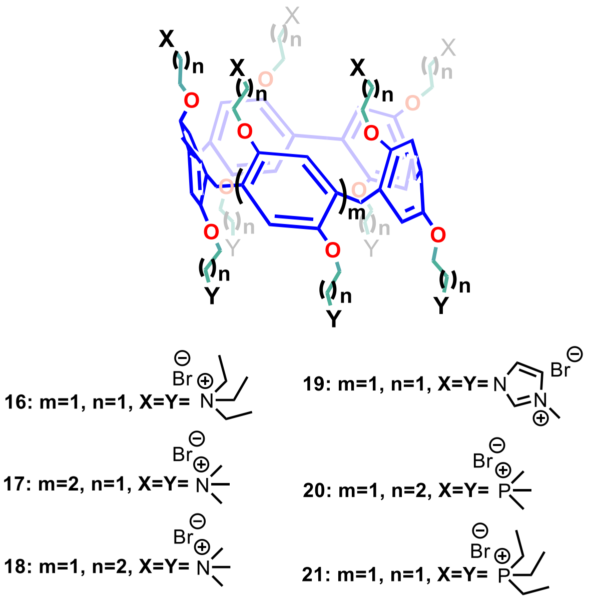

Scheme S5. The cationic and pillar[5]arene derivatives **16-21** that were examined previously[2,6]

Table S1. Biofilm inhibitory activity of cationic pillar[5,6]arene derivatives **16-21**.

Compound	$MBIC_{50}$ values in µM (µg/mL)	
	S. aureus ATCC 33592	*E. faecalis* ATCC 29212
**16**	1.5 (4)	1.5 (4)
**17**	0.4 (1)	0.4 (1)
**18**	1.66 (4)	1.66 (4)
**19**	0.8 (2)	0.4 (1)
**20**	1.55 (4)	1.55 (4)
**21**	1.33 (4)	0.67 (2)

Each $MBIC_{50}$ value is a mean of at least three independent experiments each including five replicates of each concentration

4. Biological assays

(a) Analysis of biofilm inhibition

The antibiofilm activity assay was performed as described previously[2,6,7], with minor modifications. Briefly, the tested bacterial strains were grown from frozen stocks in Brain-heart infusion medium (BHI) over night at 37 °C in 5% CO_2. Then, 100 µl of serial 1:2 dilutions of each compound in Tryptic soy broth (TSB) + 1% glucose (32, 16, 8, 4, 2, 1, and 0.5 µg/ml) were prepared in flat-bottomed 96-well microplates (Costar, Corning). Control wells with no compounds and wells without bacteria containing each tested concentration of the compounds (blanks) were also prepared. An equal volume (100 µl) of bacterial suspensions in TSB + 1% glucose was added to each well (final OD_{600} = 0.1). After incubation for 24 h at 37 °C in 5% CO_2 under aerobic conditions, spent media and free-floating bacteria were removed by turning over the plates. The wells were vigorously rinsed at least four times with doubly distilled water (DDW).

- Crystal violet assay

0.4% crystal violet (200 µl) solution was added to each well. After 45 min, wells were vigorously rinsed three times with DDW to remove unbound dye. After adding 200 µl of 33% acetic acid to each well, the plate was shaken for 15 min to release the dye. Biofilm formation was quantified by measuring the difference between absorbance of untreated and treated bacterial samples for each tested concentration of the compounds and the absorbance of appropriate blank well at 600 nm (A_{600}) using Tecan plate reader. The $MBIC_{50}$ was defined as the lowest concentration at which at least 50% reduction in biofilm formation was measured compared to untreated cells. Each concentration of compound was tested in five replicates, and at least three independent experiments were performed. Figures S45-S49 and Figures S50-S54 present the crystal violet assay for measuring the ability of the different cationic pillar[5,6]arenes synthesized to inhibit biofilm formation in methicillin resistant *S. aureus (MRSA)* and *E. faecalis*, respectively.

MRSA 33592

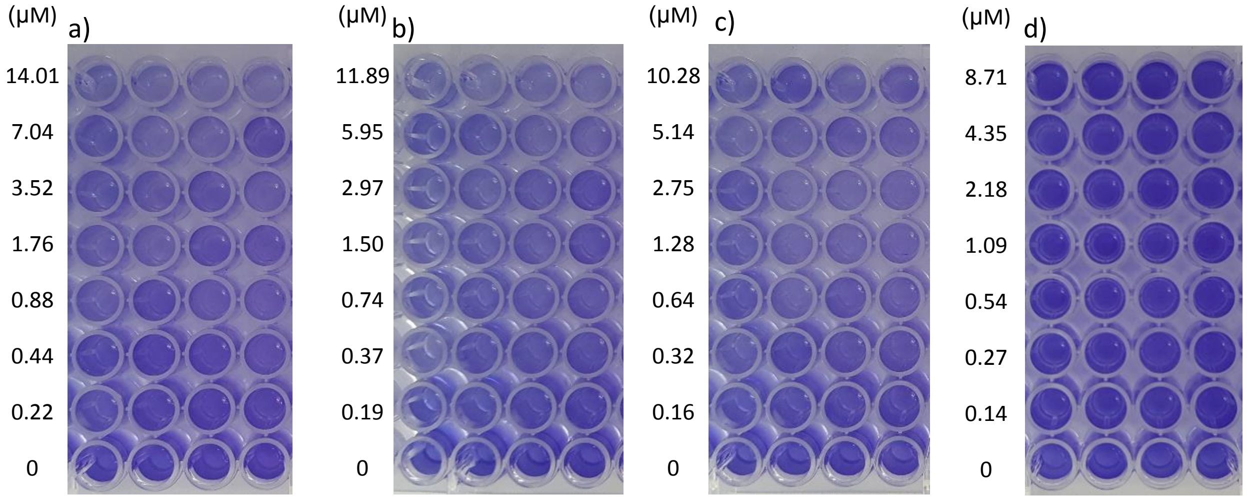

Figure S56. Biofilm formation of *S. aureus* ATCC 33592 in the presence of increasing concentrations (μM) of pillar[n]arene derivatives: (a) compound **1**, (b) compound **2**, and (c) compound **3** and (d) compound **4**. All the wells were stained with crystal violet.

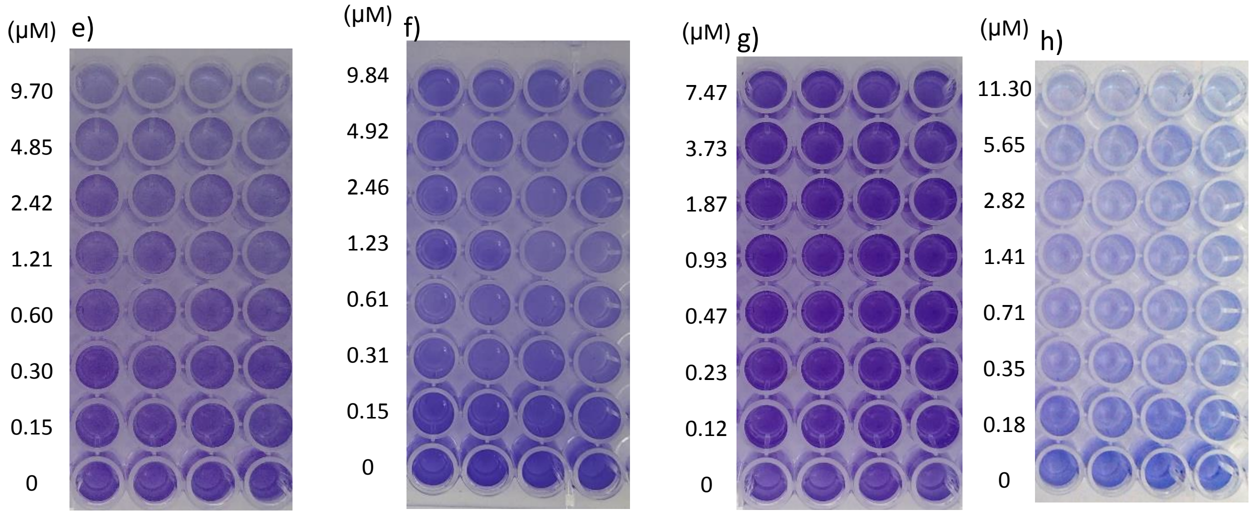

Figure S57. Biofilm formation of *S. aureus* ATCC 33592 in the presence of increasing concentrations (μM) of pillar[n]arene derivatives: (e) compound **5**, (f) compound **6**, and (g) compound **7** and (h) compound **8**. All the wells were stained with crystal violet.

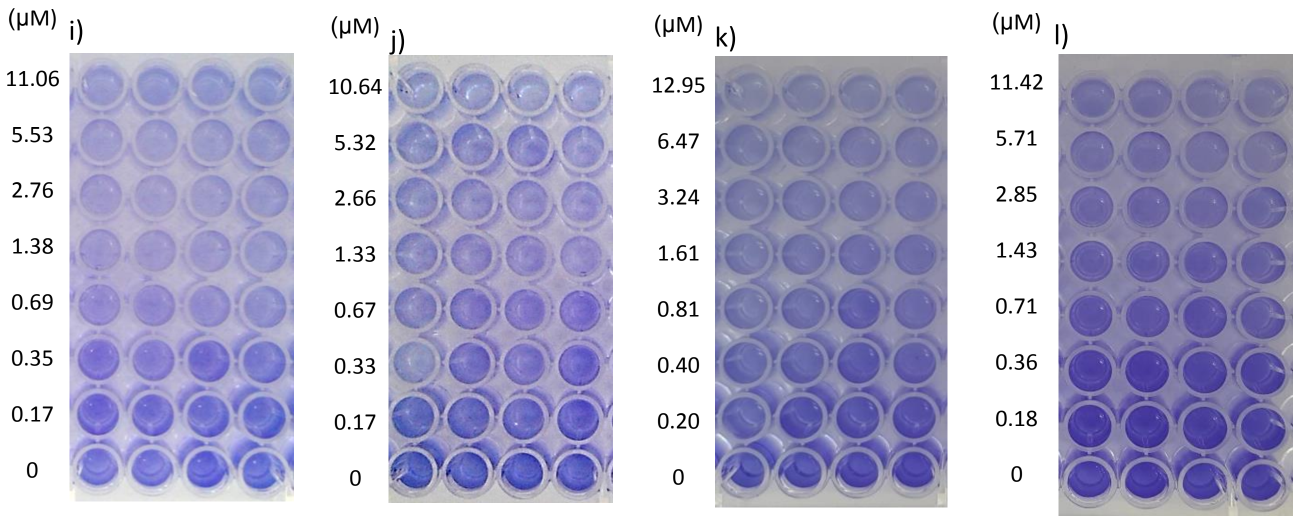

Figure S58. Biofilm formation of *S. aureus* ATCC 33592 in the presence of increasing concentrations (μM) of pillar[n]arene derivatives: (i) compound **9**, (j) compound **10**, (k) compound **11** and (l) compound **12**. All the wells were stained with crystal violet.

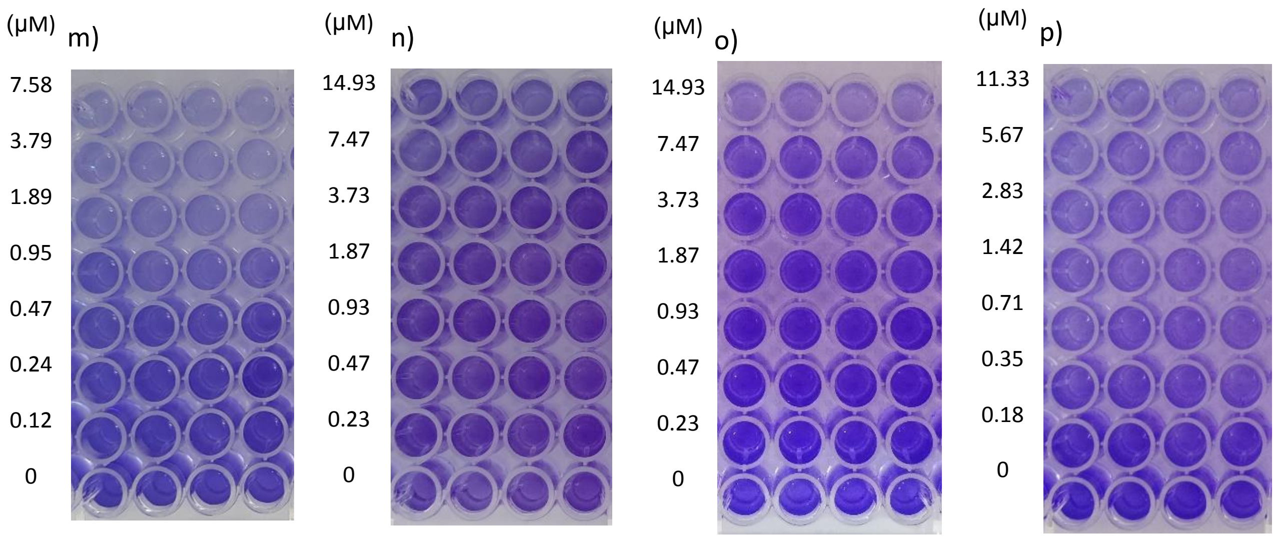

Figure S59. Biofilm formation of *S. aureus* ATCC 33592 in the presence of increasing concentrations (μM) of pillar[n]arene derivatives: (m) compound **13**, (n) compound **14a**, (n) compound **14b** and (l) compound **15**. All the wells were stained with crystal violet.

E.F. 29212

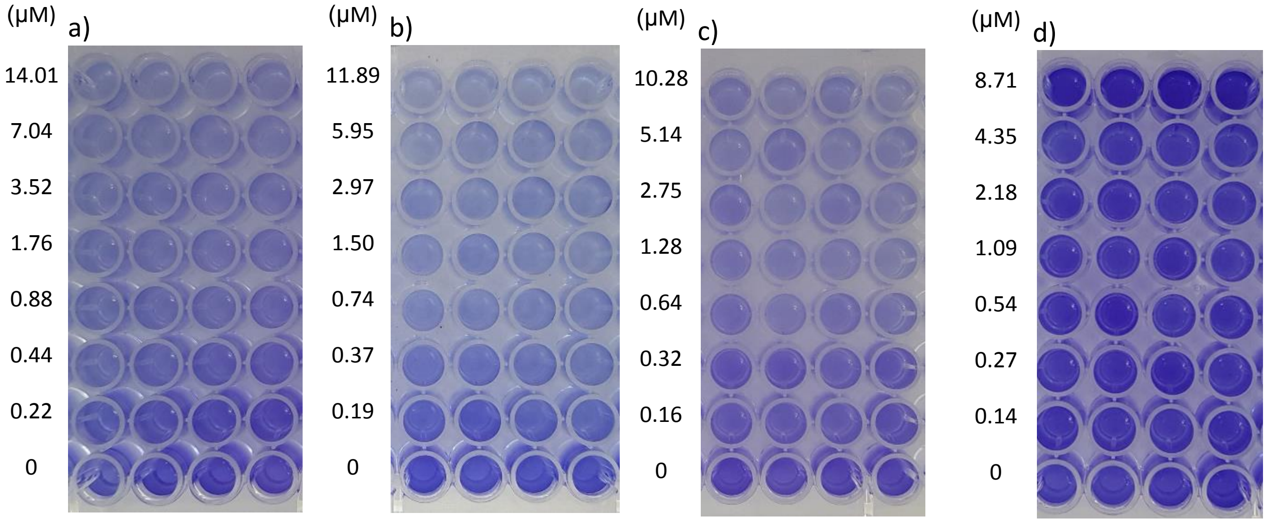

Figure S60. Biofilm formation of *E. faecalis* ATCC 29212 in the presence of increasing concentrations (μM) of pillar[n]arene derivatives: (a) compound **1**, (b) compound **2**, (c) compound **3** and (d) compound **4**. All the wells were stained with crystal violet.

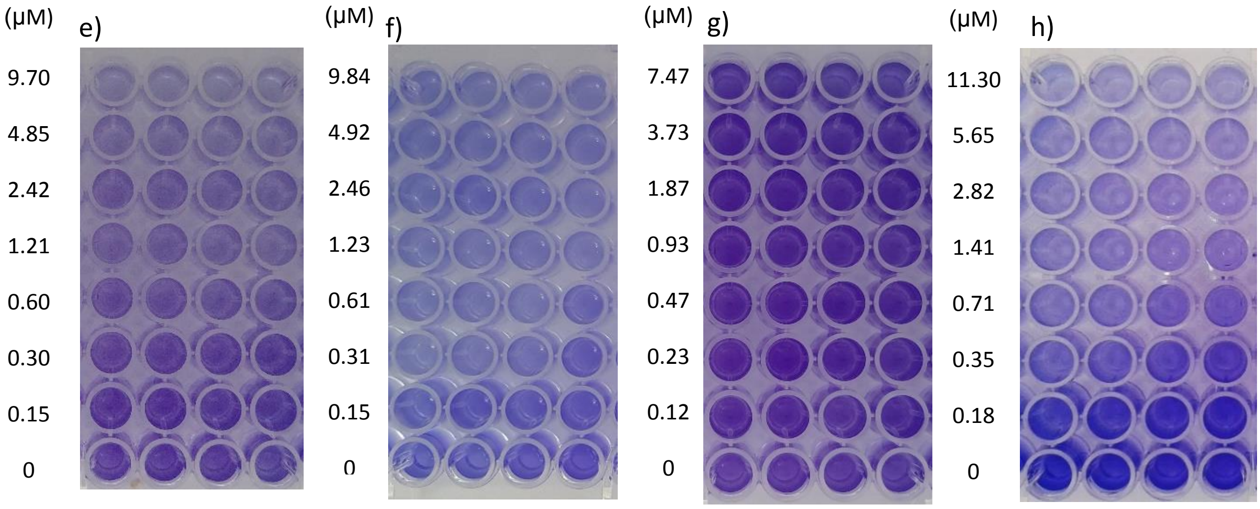

Figure S61. Biofilm formation of *E. faecalis* ATCC 29212 in the presence of increasing concentrations (μM) of pillar[n]arene derivatives: (e) compound **5**, (f) compound **6**, (g) compound **7** and (h) compound **8**. All the wells were stained with crystal violet.

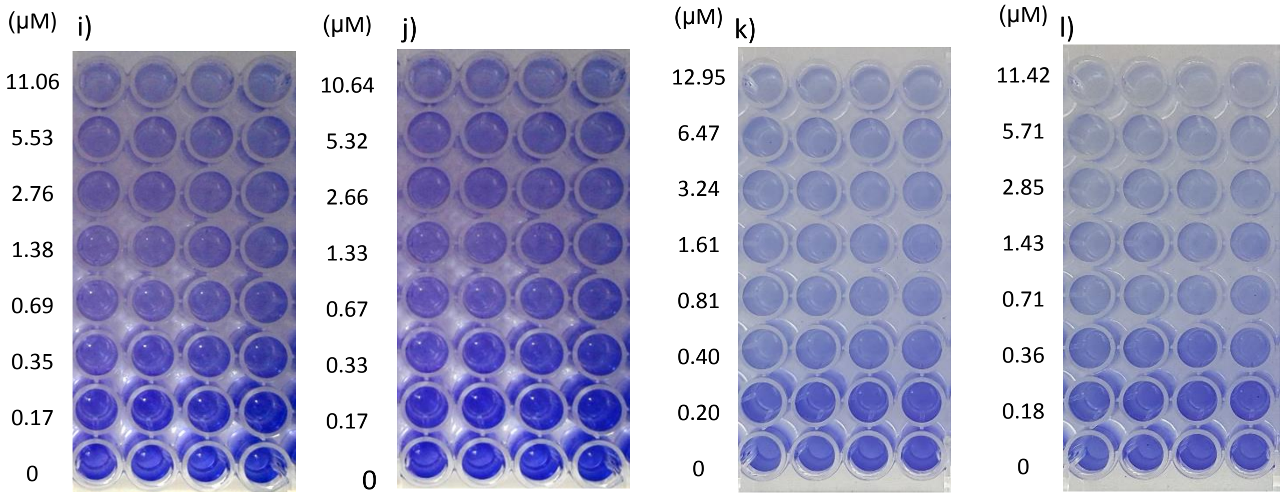

Figure S62. Biofilm formation of *E. faecalis* ATCC 29212 in the presence of increasing concentrations (µM) of pillar[n]arene derivatives: (i) compound **9**, (j) compound **10**, (k) compound **11** and (l) compound **12**. All the wells were stained with crystal violet.

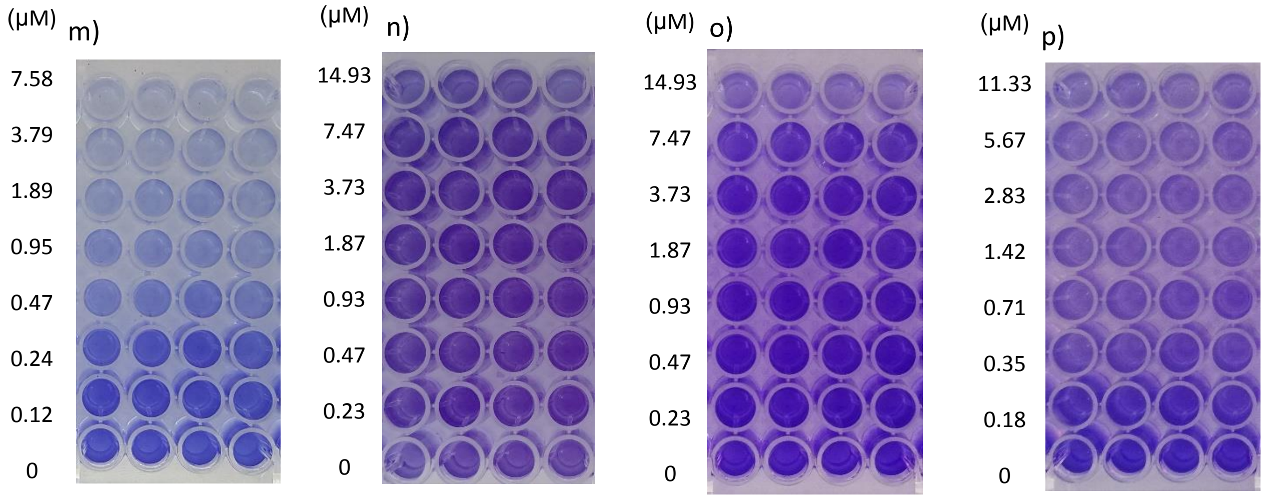

Figure S63. Biofilm formation of *E. faecalis* ATCC 29212 in the presence of increasing concentrations (µM) of pillar[n]arene derivatives: (m) compound **13**, (n) compound **14a**, (o) compound **14b** and (p) compound **12**. All the wells were stained with crystal violet.

- Biofilm formation vs. compound-concentration

a)

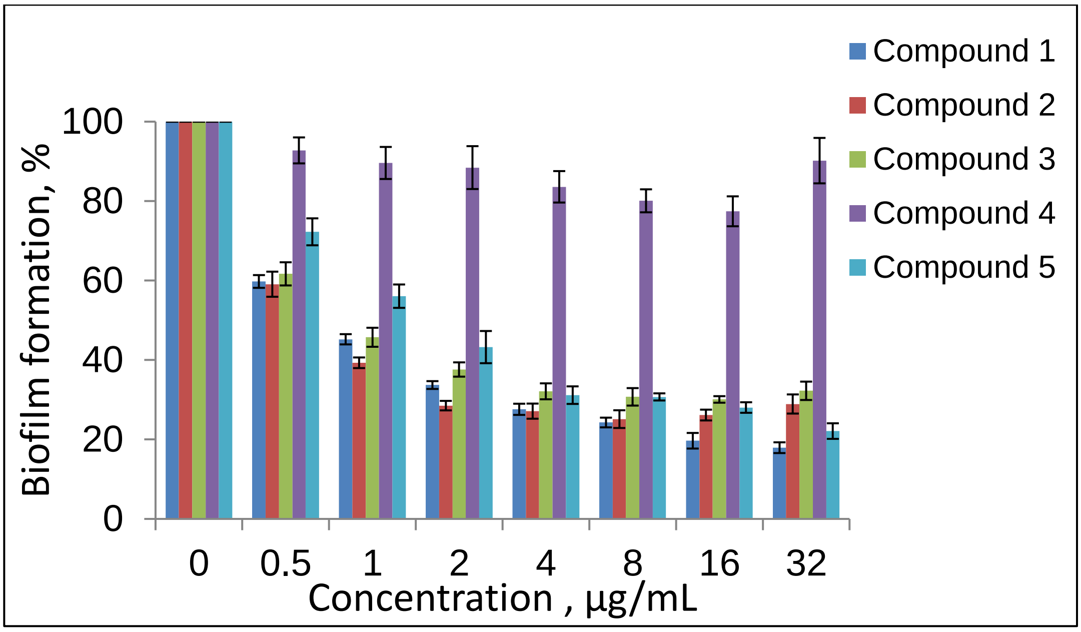

b)

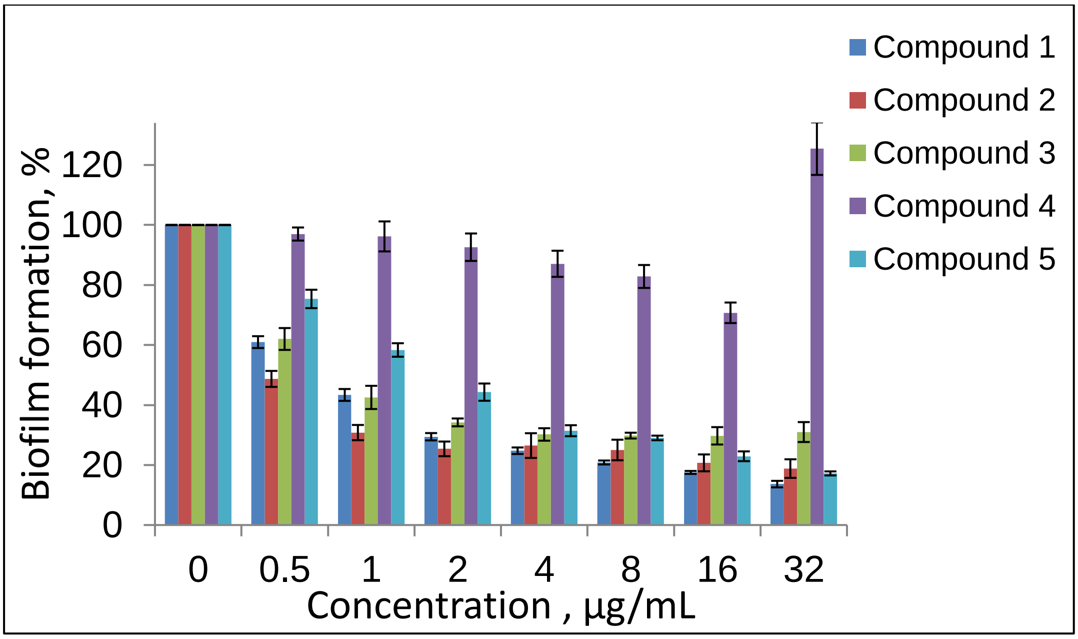

Figure S64. Biofilm formation by (a) *S. aureus* ATCC 33592 (MRSA) and (b) *E. faecalis* ATCC 29212 evaluated using the double-dilution method with starter inoculum of 1 : 100 (OD600 = 0.01) in the presence of compounds **1-5**. Values are mean ± standard error of at least 3 experiments of 5 repetition each.

a)

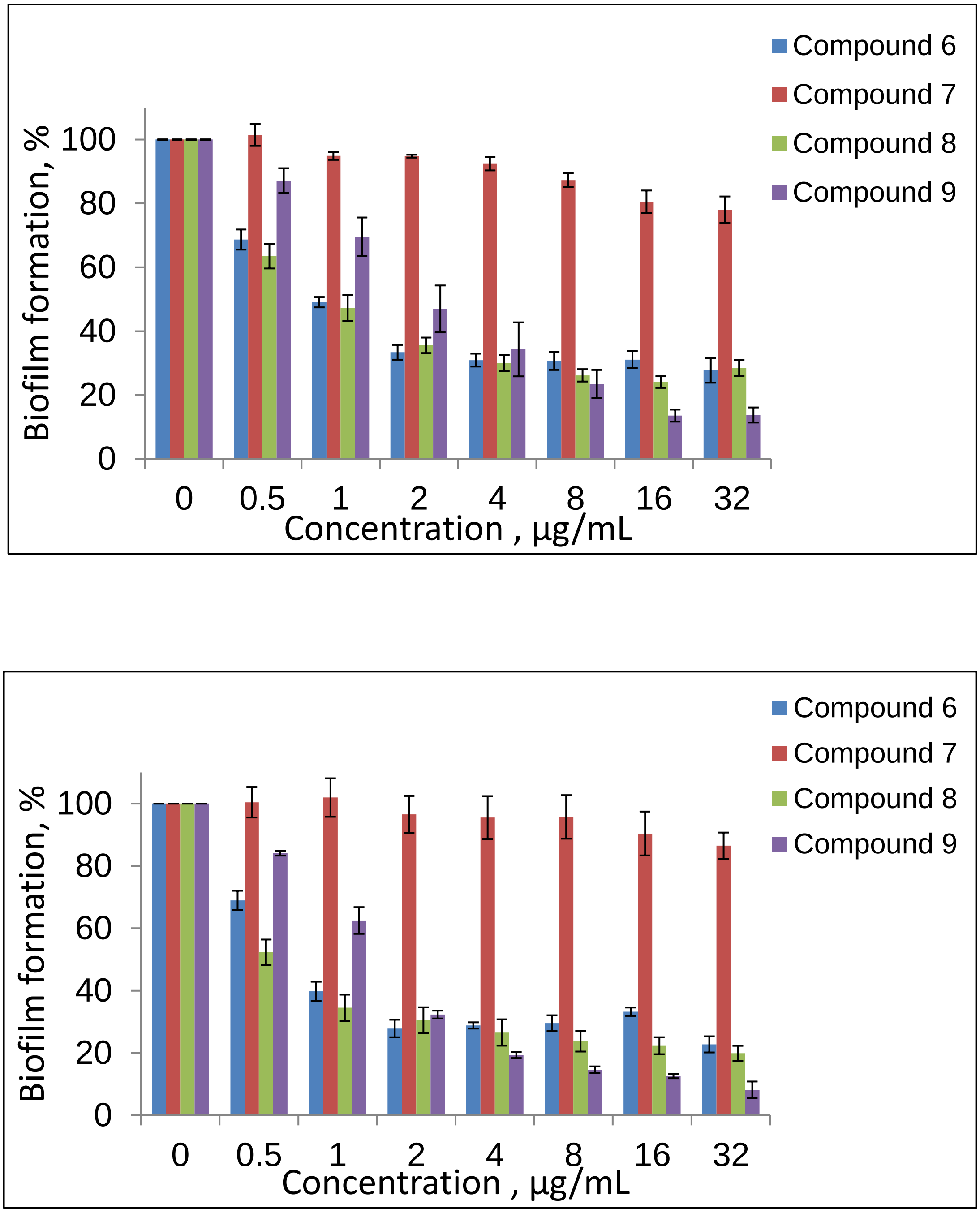

b)

Figure S65. Biofilm formation by (a) *S. aureus* ATCC 33592 (MRSA) and (b) *E. faecalis* ATCC 29212 evaluated using the double-dilution method with starter inoculum of 1 : 100 (OD600 = 0.01) in the presence of compounds **6-9**. Values are mean ± standard error of at least 3 experiments of 5 repetition each.

a)

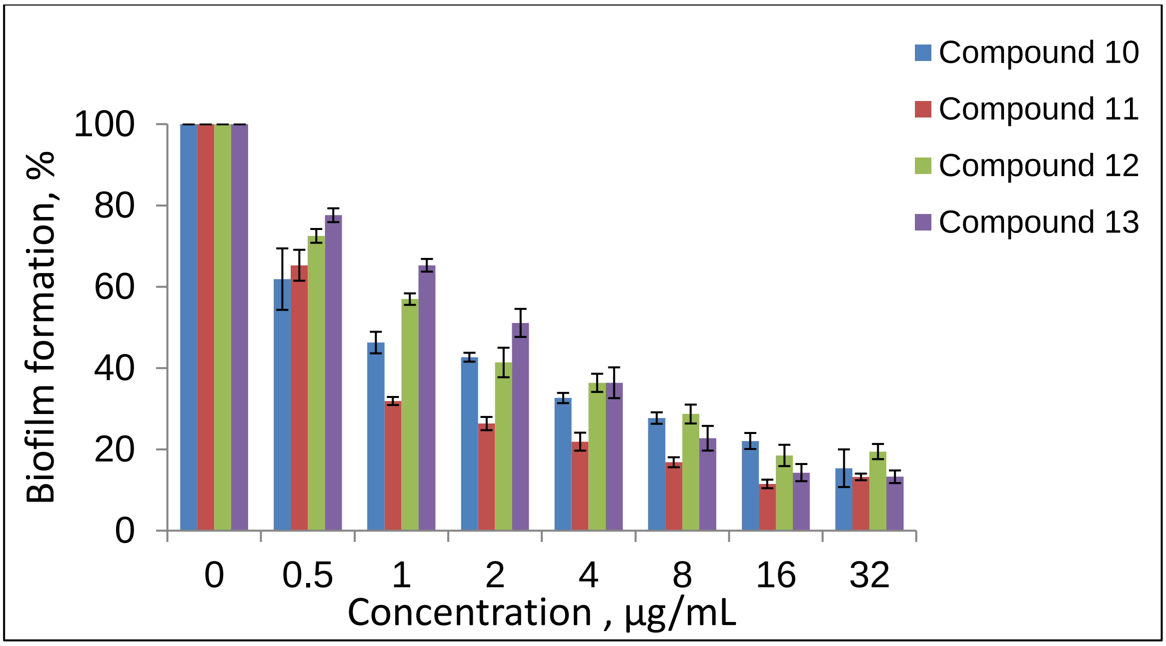

b)

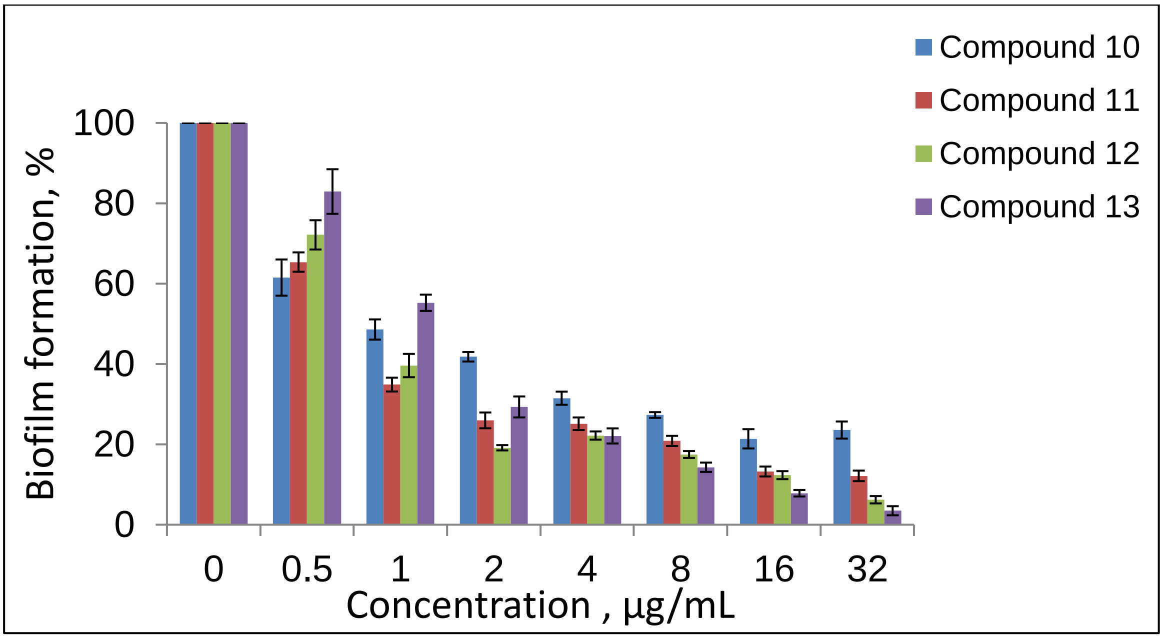

Figure S66. Biofilm formation by (a) *S. aureus* ATCC 33592 (MRSA) and (b) *E. faecalis* ATCC 29212 evaluated using the double-dilution method with starter inoculum of 1 : 100 (OD600 = 0.01) in the presence of compounds **10-13**. Values are mean ± standard error of at least 3 experiments of 5 repetition each.

a)

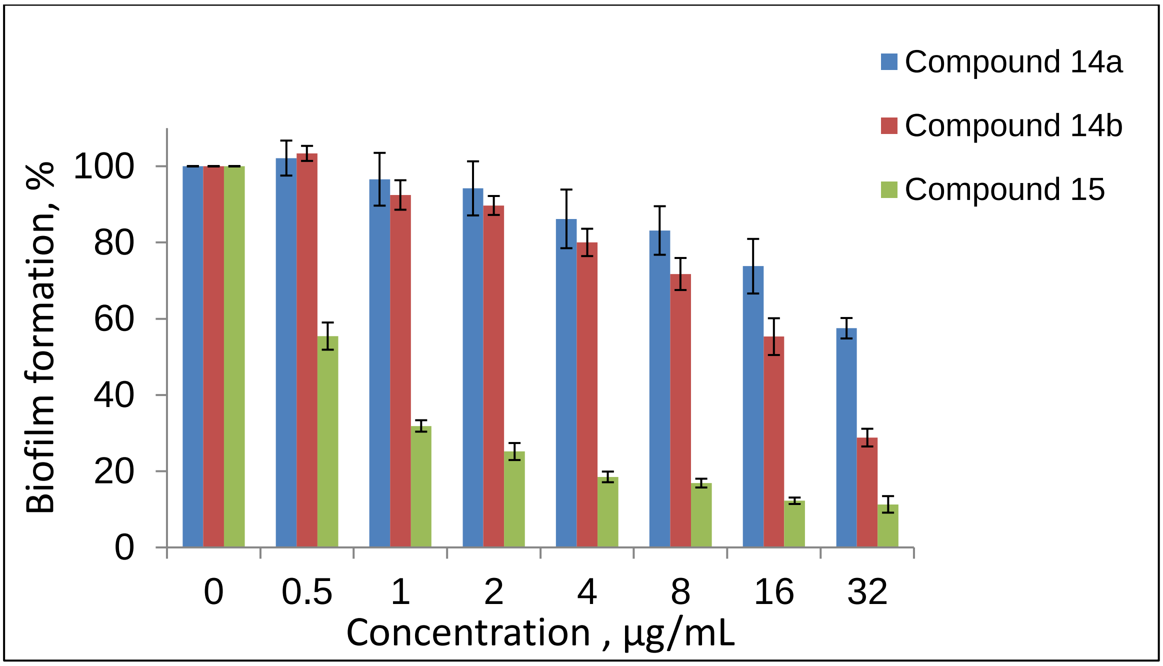

b)

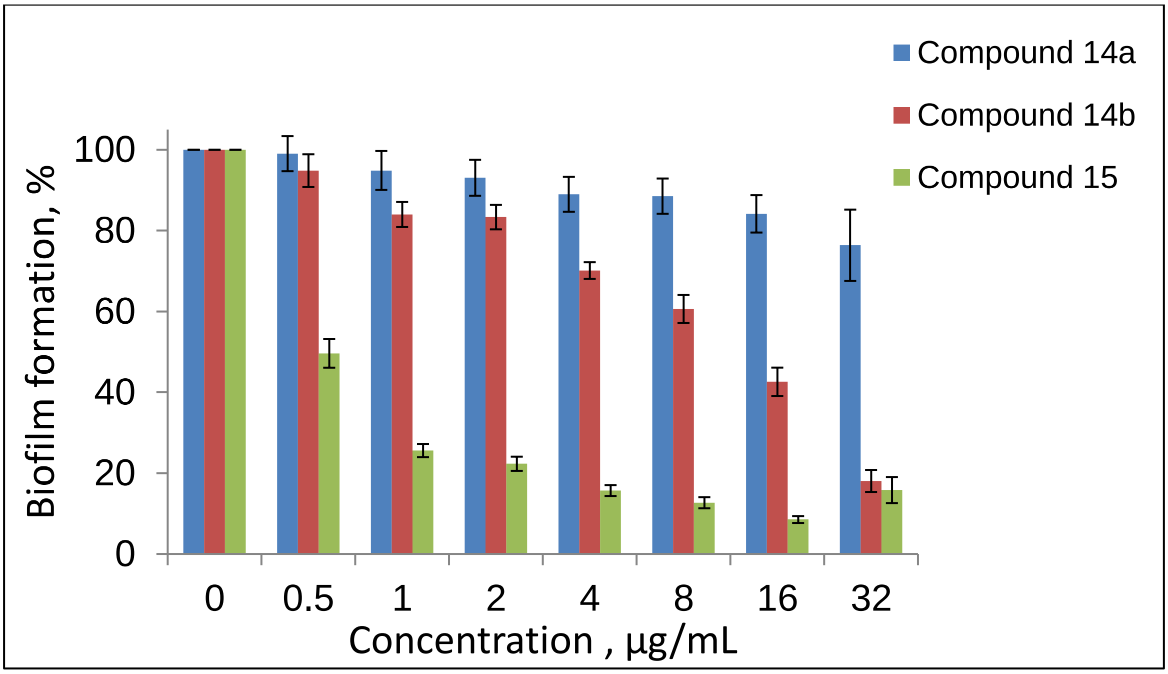

Figure S67. Biofilm formation by (a) *S. aureus* ATCC 33592 (MRSA) and (b) *E. faecalis* ATCC 29212 evaluated using the double-dilution method with starter inoculum of 1 : 100 (OD600 = 0.01) in the presence of compounds **14a, 14b** and **15**. Values are mean ± standard error of at least 3 experiments of 5 repetition each.

(b) Rat red blood cell hemolysis assay

The hemolysis was performed as previously described with minor modifications.[9] Briefly a sample of rat red blood cells (2% w/w in PBS) were incubated with each of the tested compounds (CTAB and compounds **1-8** and **10-15**) for 1 h at 37 °C in 5% CO_2 using the double dilution method starting at a concentration of 256 µg/mL. The negative control was PBS, and the positive control was 1% v/v solution of Triton X-100 (which induced 100% hemolysis). Following centrifugation (2000 rpm, 10 min, ambient temperature), the supernatant was removed and absorbance at 550 nm was measured using a microplate reader (Genios, TECAN). Graph of percentage of hemoglobin released vs. compounds' concentrations, relative to the positive control (Triton X-100), were obtained from two independent experiments performed in triplicate. The results of these experiments are summarized in Figure S68 and S69. Note that Figure S69 presents the HC_{50} values extracted from the data presented in Figure S68.

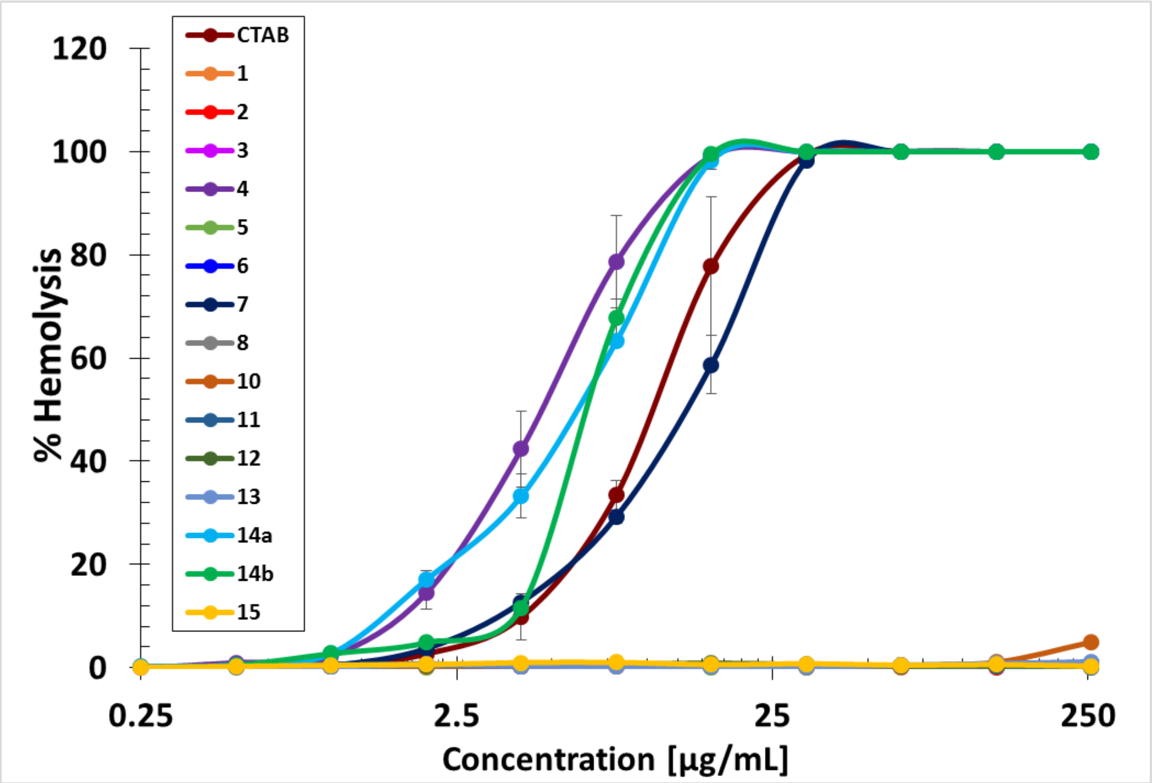

Figure S68. Hemolysis percentages caused by CTAB, compounds **1-8** and **10-15** as function of their amount (given in µg/ml) in rat RBCs. The results show that only CTAB and compounds **4**, **7**, **14a** and **14b** are hemolytic. Compound **10** shows some hemolytic effect at 256µg/ml. For compounds definition, see Scheme 1 in the paper

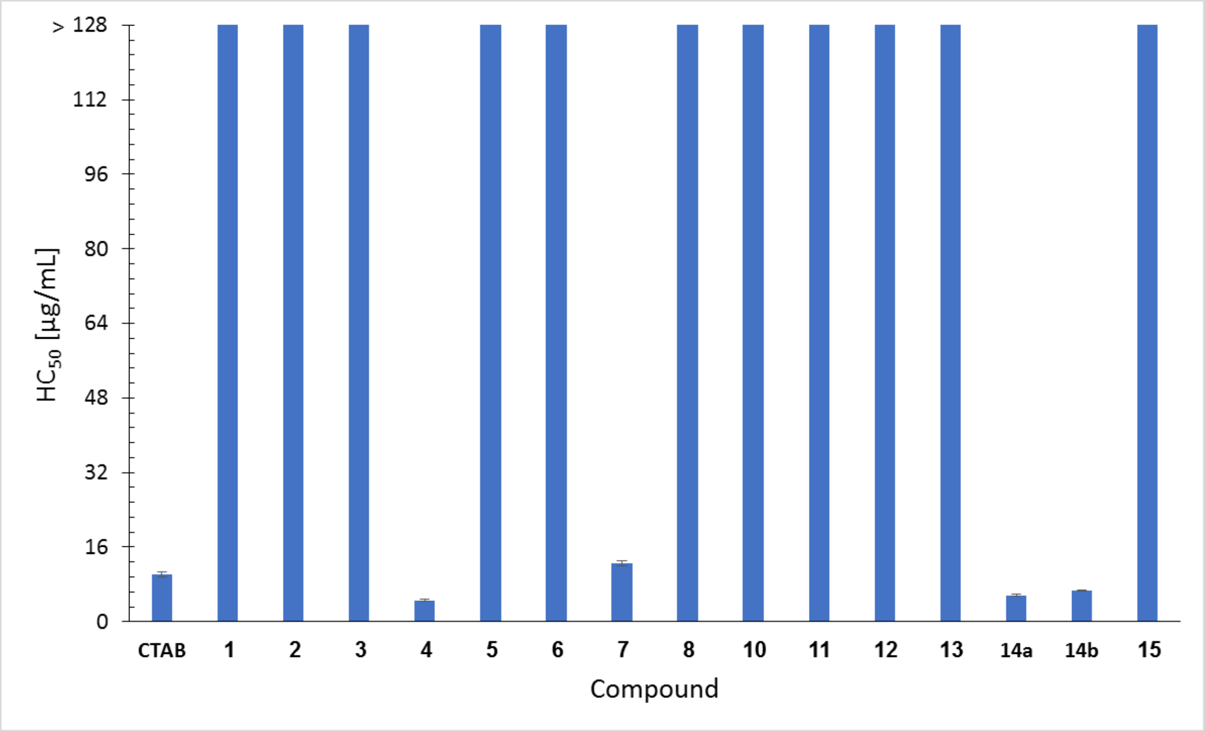

Figure S69. The HC_{50} values (given in µg/ml) extracted for CTAB and compounds **1-8** and **10-15** in rat RBCs. The results are expressed as means ± standard error. For compounds definition, see Scheme 1 in the paper.

(c) Effect on bacterial growth

The tested bacterial strains were first grown from the frozen stock in Brain Heart Infusion (BHI) broth for 24 h at 37 °C. Volumes of 100 µl of serial 1:2 dilutions (64, 32, 16, 8, 4, 2 and 1µg/ml) of the selected compounds in Tryptic Soy Broth (TSB) + 1% glucose were prepared in a flat-bottomed 96-well microplates (Corning). Next, an equal volume (100 µl) of bacterial suspension in TSB + 1% glucose was added to each well to a final OD_{600} of 0.01. Control wells with no compounds and wells without bacteria (blanks) were also prepared. During a 24 h incubation at 37 °C, growth kinetics were monitored by recording optical density at wavelength 600 nm (OD_{600}) using a Tecan plate reader. Each concentration was tested in triplicate, and the results are shown as an average of two independent experiments.

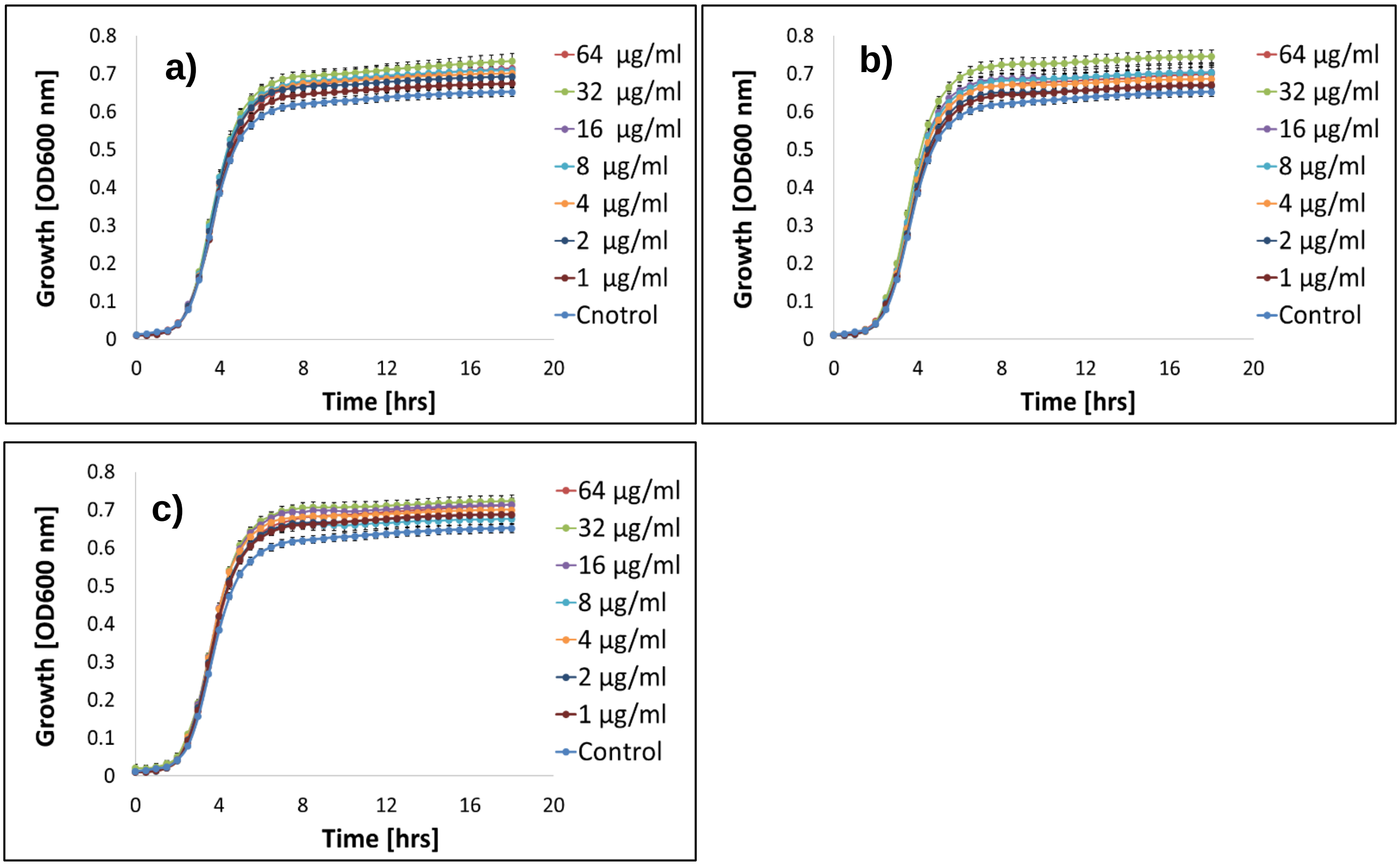

Figure S70. Growth curves of *S. aureus* ATCC 33592 (MRSA) in the presence of: (a) compound **2**, (b) compound **12** and (c) compound **15**. Bacteria were incubated with increasing concentrations of the compounds (1-64 µg/ml) for 18 h at 37 °C. All three compounds show no effect on bacterial growth.

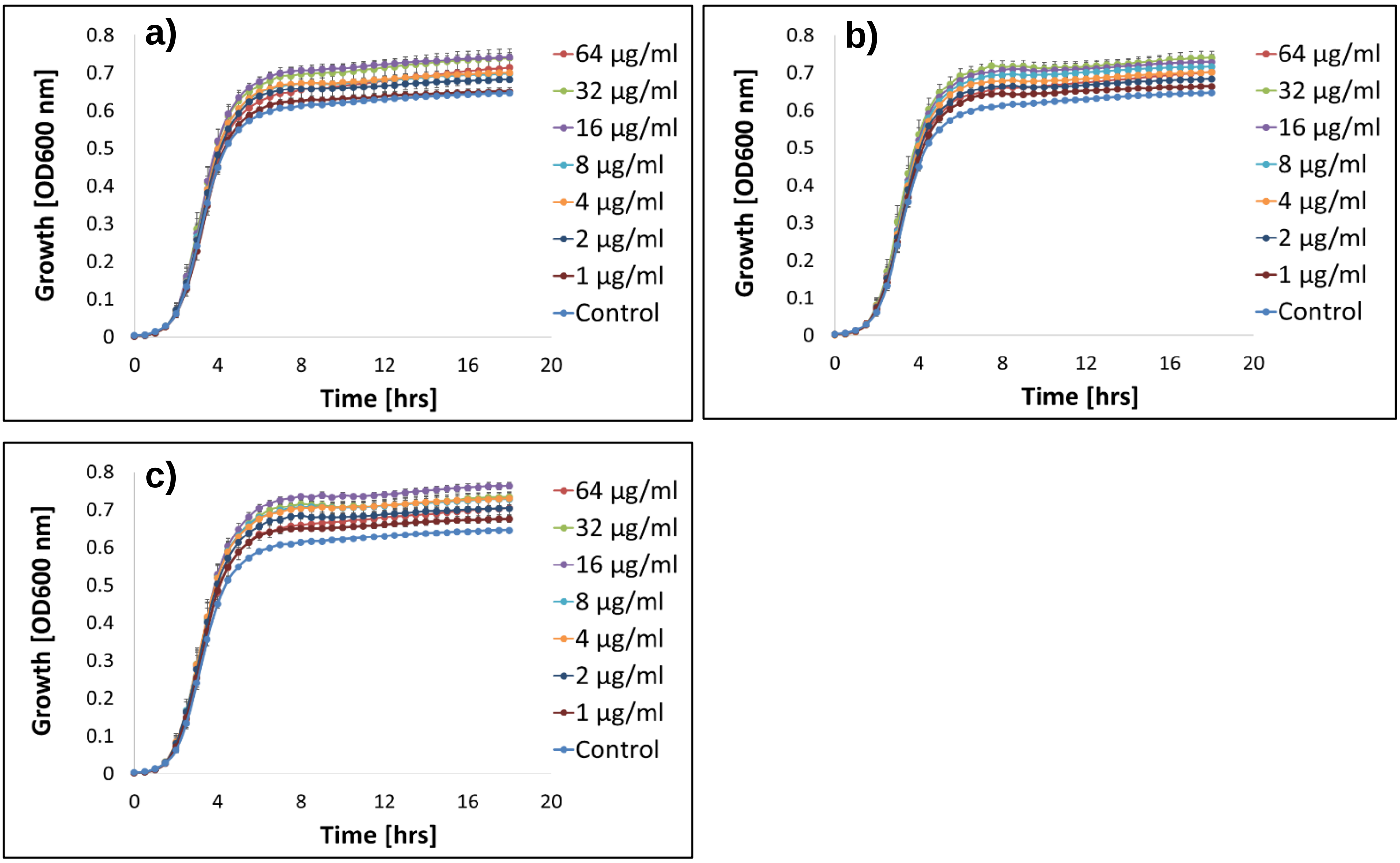

Figure S71. Growth curves of *E. faecalis ATCC 29212* in the presence of: (a) compound **2**, (b) compound **12** and (c) compound **15**. Bacteria were incubated with increasing concentrations of the compounds (1-64 µg/ml) for 18 h at 37 °C. All three compounds show no effect on bacterial growth.

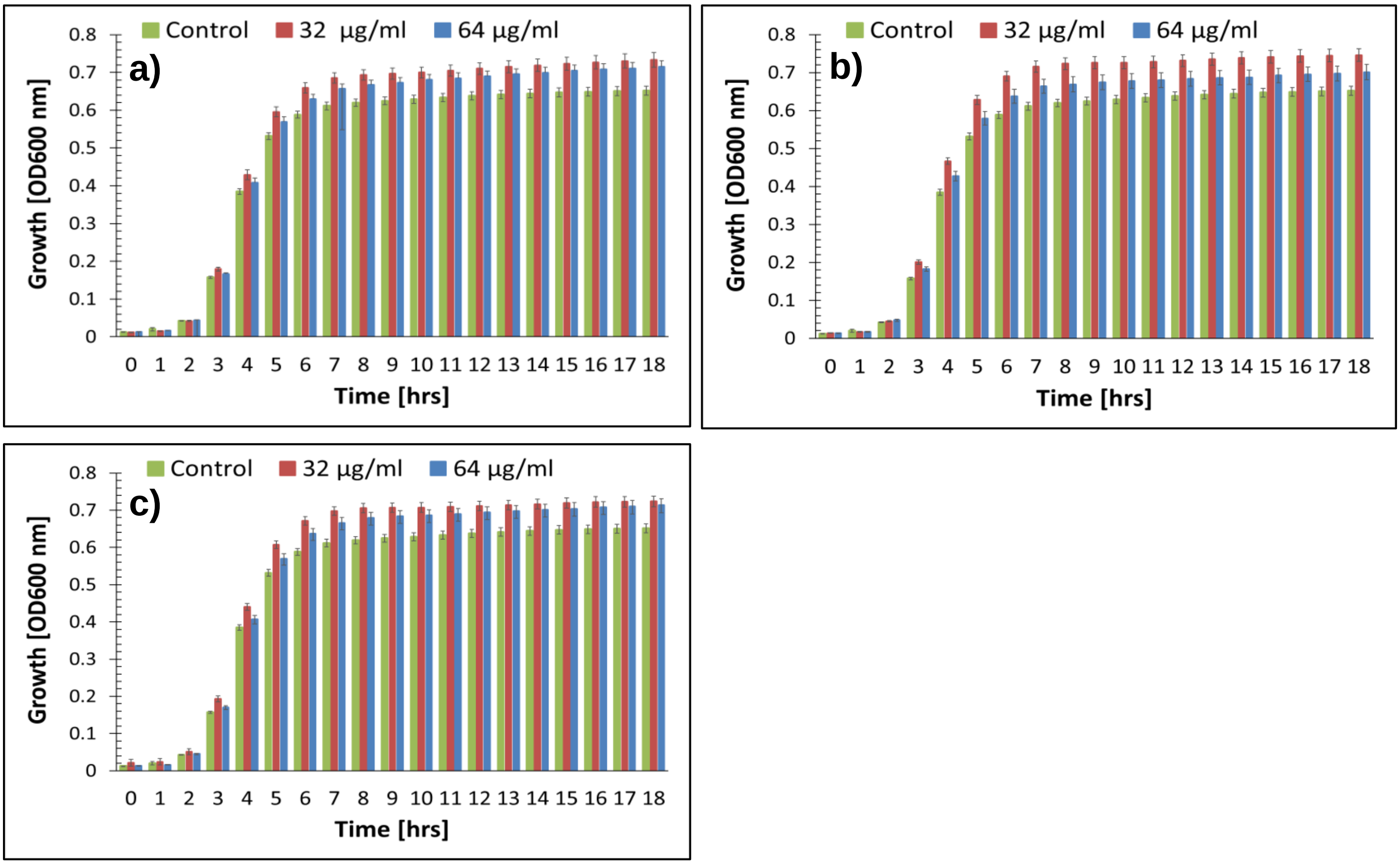

Figure S72. Growth curves of *S. aureus* ATCC 33592 (MRSA) in the presence of: (a) compound **2**, (b) compound **12** and (c) compound **15**. Bacteria were incubated with the compounds (32 and 64 µg/mL) for 18 h at 37 °C. All three compounds show no effect on bacterial growth.

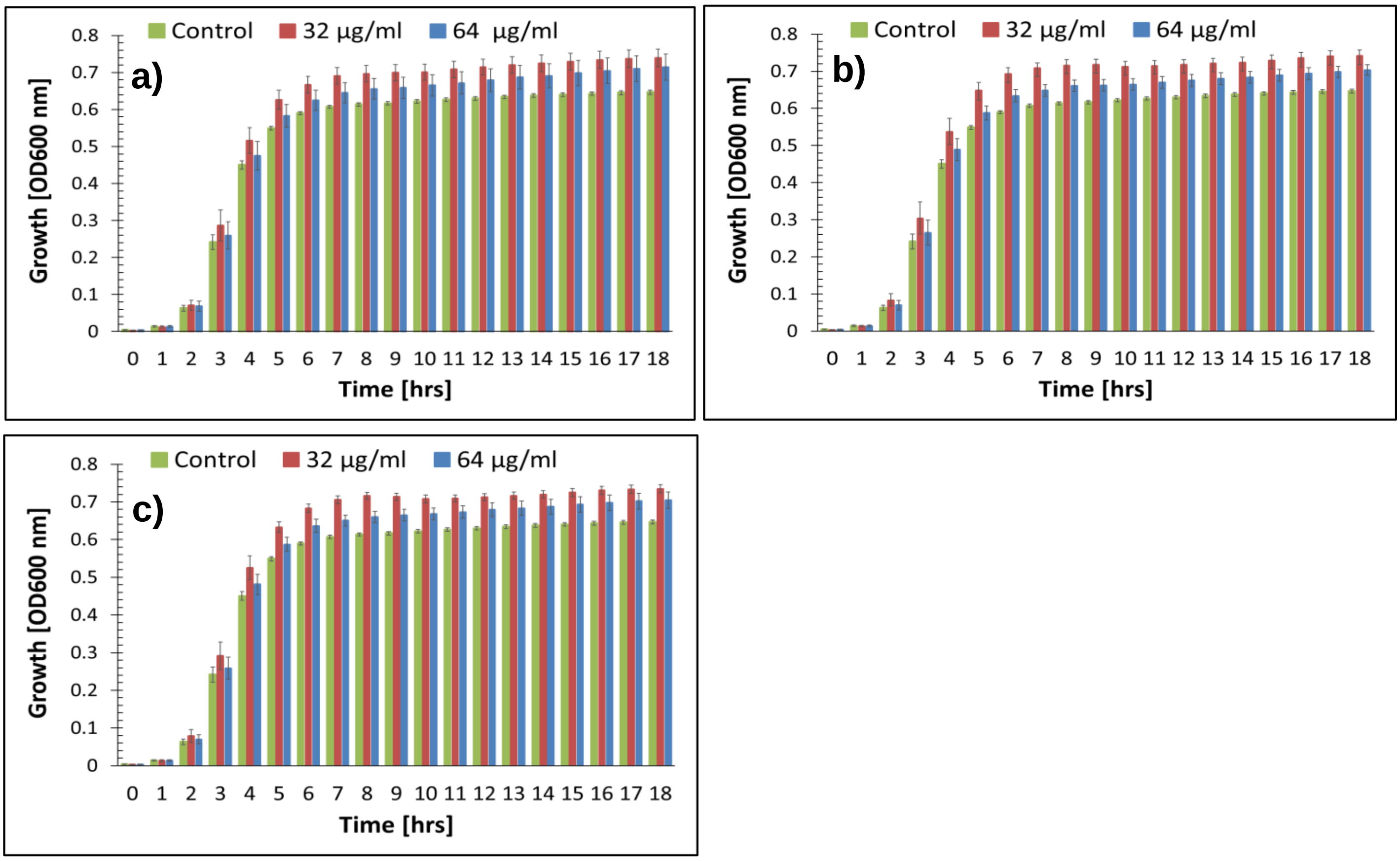

Figure S73. Growth curves of *E. faecalis ATCC 29212* in the presence of: (a) compound **2**, (b) compound **12** and (c) compound **15**. Bacteria were incubated with the compounds (32 and 64 µg/mL) for 18 h at 37 °C. All three compounds show no effect on bacterial growth.

5. Analytical HPLC chromatograms

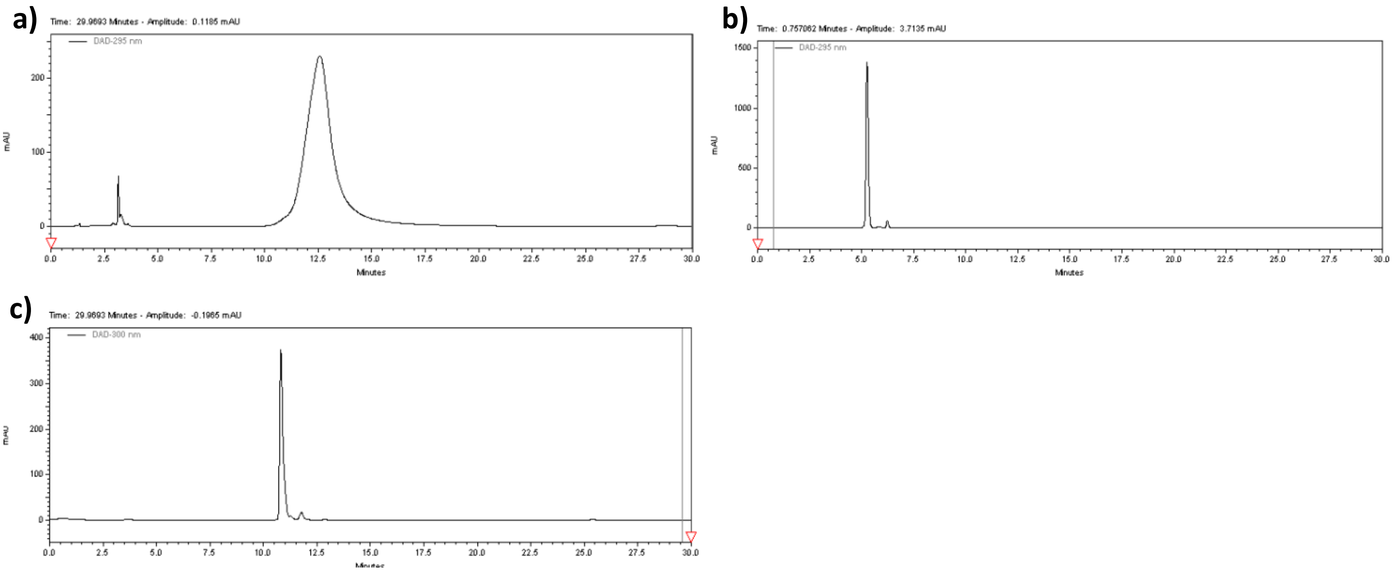

Figure S74. Analytic reversed-phase HPLC chromatograms of: a) compound **4** (30 - 100% ACN in water. Injected solution contained 9% DMSO), b) compound **8** (30 - 100% ACN in water), c) compound **11** (10 - 40% ACN in water)

6. References

1) Yao, Y., Xue, M., Chi, X., Ma, Y., He, J., Abliz, Z., Huang, F. (2012) A new water-soluble pillar[5]arene: synthesis and application in the preparation of gold nanoparticles. *Chem. Commun.*, *48*, 6505-6507, DOI: 10.1039/C2CC31962D.
2) Joseph, R., Kaizerman, D., Herzog, I. M., Hadar, M., Feldman, M., Fridman, M., Cohen, Y. (2016) Phosphonium pillar[5]arenes as a new class of efficient biofilm inhibitors: importance of charge cooperativity and the pillar platform. *Chem. Commun.*, *52*, 10656-10659, DOI: 10.1039/C6CC05170G.
3) Qin, A., Lam, J. W. Y., Jim, C. K. W., Zhang, L., Yan, J., Haaussler, M., Liu, J., Dong, Y., Liang, D., Chen, E., Jia, G., Tang, B. Z. (2008) Hyperbranched Polytriazoles: Click Polymerization, Regioisomeric Structure, Light Emission, and Fluorescent Patterning. *Macromolecules*, *41*, 3808-3822, DOI: 10.1021/ma800538m.
4) Chen, W., Zhang, Y., Li, J., Lou, X., Yu, Y., Jia, X., Li, C. (2013) Synthesis of a cationic water-soluble pillar[6]arene and its effective complexation towards naphthalenesulfonate guests. *Chem. Commun.*, *49*, 7956-7958, DOI: 10.1039/C3CC44328K.
5) Ogoshi, T., Ueshima, N., Yamagishi, T.A., Toyota, Y., Matsumi, N. (2012) Ionic liquid pillar[5]arene: its ionic conductivity and solvent-free complexation with a guest. *Chem. Commun.*, *48*, 3536-3538, DOI: 10.1039/C2CC30589E.

6) Joseph, R., Naugolny, A., Feldman, M., Herzog, I. M., Fridman, M., Cohen, Y. (2016) Cationic pillararenes potently inhibit biofilm formation without affecting bacterial growth and viability. J. Am. Chem. Soc., 138, 754-757, DOI: 10.1021/jacs.5b11834.
7) Feldman, M., Tanabe, S., Howell, A., Grenier, D. C. (2012) Cranberry proanthocyanidins inhibit the adherence properties of Candida albicans and cytokine secretion by oral epithelial cells. *BMC Complement. Altern. Med., 12*, 6, DOI: 10.1186/1472-6882-12-6
8) Hadar, M., Kaizerman-Kane, D., Zafrani, Y., Cohen, Y., (2020) Temperature-dependent and pH-responsive pillar[5]arene-based complexes and hydrogen-bonded supramolecular pentagonal boxes in water. Chem. Eur. J., 26, DOI: 10.1002/chem 202000972.
9) Steinbuch, K., Benhamou, R. I., Levin, L., Stein, R., Fridman, M. (2018) Increased Degree of Unsaturation in the Lipid of Antifungal Cationic Amphiphiles Facilitates Selective Fungal Cell Disruption. *ACS Infect. Dis*., *4,* 825-836, DOI: 10.1021/acsinfecdis.7b00272.
